# ESCRT‐Mimetic Nanodegrader Targets STING for Anti‐Inflammatory Therapy

**DOI:** 10.1002/advs.202523601

**Published:** 2026-03-24

**Authors:** Fuyuan Zhou, Qiming Zhai, Zhihao Yao, Qing Li, Yan Yang, Si Wang, Chao Huang, Liangjing Xin, Tao Chen, Jinlin Song

**Affiliations:** ^1^ Chongqing Key Laboratory of Oral Diseases Chongqing Municipal Key Laboratory of Oral Biomedical Engineering of Higher Education Chongqing Municipal Health Commission Key Laboratory of Oral Biomedical Engineering Chongqing Technology Innovation Center of Smart Dental Medical Devices Innovation and Transformation of Dental Medical Devices Engineering Research Center of Chongqing Education Commission of China The Affiliated Stomatological Hospital of Chongqing Medical University Chongqing China; ^2^ Chongqing Key Laboratory of Natural Product Synthesis and Drug Research School of Pharmaceutical Sciences Chongqing University Chongqing China

**Keywords:** autophagy‐tethering chimera, inflammation, lipid nanoparticle, macrophage targeting, targeted protein degradation

## Abstract

Excessive STING activation underlies the pathogenesis of diverse inflammatory diseases, yet conventional inhibitors often fail to restore its physiological degradation. To address this, we designed a biomimetic STING‐directed autophagy‐targeting chimera (STING‐ATTEC) that recapitulates the endogenous ESCRT‐mediated degradation pathway, with computational modeling providing preliminary guidance for molecular optimization. The optimized STING‐ATTEC was encapsulated within folate‐modified cationic lipid nanoparticles (FA‐LNP^+^), formulated with DOTAP to enhance autophagic activity and promote lysosomal trafficking. This combined strategy synergistically amplified STING degradation, leading to potent suppression of inflammatory signaling, mitigation of tissue damage, and promotion of tissue regeneration across multiple disease models. These findings illustrate a cooperative material‐based strategy that enhances autophagy and enables targeted protein degradation, positioning lysosome‐targeting degraders as a promising translational modality for immunomodulatory therapy.

## Graphical abstract

A nanoplatform‐enabled targeted protein degradation strategy is presented to regulate aberrant STING signaling. STING‐ATTEC induces selective autophagic degradation of STING via formation of a STING–ATTEC–LC3 ternary complex, while the cationic FA‐LNP^+^ system enhances LC3 generation and targeted delivery. Together, this synergistic approach efficiently suppresses STING activation and shows therapeutic potential in inflammatory diseases.

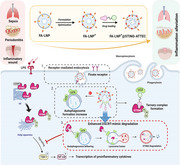

## 1 Introduction

The cGAS‐STING signaling pathway is a pivotal regulator of innate immune responses to pathogens and a central driver of inflammation in diverse diseases [1, 2]. Central to this pathway is the endoplasmic reticulum (ER) ‐resident protein STING, which, upon activation by the second messenger 2′,3′‐cyclic GMP‐AMP (cGAMP), initiates downstream signaling cascades involving TBK1/IRF3, NF‐κB, and JAK‐STAT pathways [3, 4, 5, 6]. This leads to robust production of pro‐inflammatory cytokines such as IL‐1β, TNF‐α, and GM‐CSF, orchestrating a potent innate immune defense [7, 8]. However, dysregulation of STING signaling is increasingly implicated in the sustained inflammation observed in autoimmune, metabolic, and degenerative disorders, underscoring its importance as both a key immune regulator and a therapeutic target [9].

The tightly regulated degradation of STING constitutes a critical regulatory mechanism for the termination of inflammatory signaling, and its dysregulation is directly implicated in the pathogenesis of STING‐associated inflammatory disorders [10]. Under basal conditions, STING dimerizes via transmembrane domains on the ER [11]. Upon cyclic dinucleotide (CDN) binding, it translocates through COPII‐coated vesicles to the ER‐Golgi intermediate compartment (ERGIC) and Golgi apparatus—sites essential for TBK1 recruitment and downstream signal initiation [12]. Subsequently, STING is shuttled to endolysosomal compartments for degradation—a process dependent on lysosomal acidification and sensitive to V‐ATPase inhibitors such as bafilomycin A1 and chloroquine [3, 13]. Mechanistically, STING degradation is orchestrated by the endosomal sorting complex required for transport (ESCRT), wherein the ESCRT‐0 subunit HRS recognizes ubiquitinated STING on endosomes, initiating a cascade that culminates in lysosomal degradation [14]. Disruption of this process—through HRS depletion or dominant‐negative VPS4A expression—inhibits STING degradation and amplifies inflammatory signaling [15]. Critically, STING's trafficking operates in a dynamic equilibrium; structural mutations impairing its recycling cause pathological accumulation in ERGIC/Golgi compartments, while lysosomal degradation defects prolong signaling residence [16]. Both scenarios drive excessive cytokine production and tissue inflammation, underscoring that loss of degradation homeostasis—not just activation—fuels STING‐dependent immunopathology [17]. Consequently, targeting STING degradation machinery presents promising therapeutic avenues for restraining inflammation.

Given the role of persistent STING activation in autoimmune and inflammatory diseases, STING‐targeting small molecules have gained attention as potential therapeutics [7, 18]. These inhibitors fall into two main classes: competitive CDN‐binding antagonists (e.g., compound 18, Astin C, SN‐011) and compounds inhibiting palmitoylation at cysteine residues (Cys88 and Cys91) within its C‐terminal transmembrane domain (e.g., C‐176, C‐170, H‐151, BPK‐21, NO_2_‐CLA) [19, 20]. However, such occupancy‐based inhibitors do not restore physiological degradation and may permit resistance and signaling rebound after treatment. In contrast, targeted protein degradation (TPD) strategies, particularly proteolysis‐targeting chimeras (PROTACs) leverage the UPS to eliminate target proteins via E3 ligase recruitment and proteasomal destruction [20, 21]. Unlike traditional occupancy‐driven inhibitors, this event‐driven strategy achieves therapeutic efficacy through substrate depletion rather than inhibition, offering advantages for previously “undruggable” targets [22, 23].

While PROTACs represent a breakthrough in targeted protein degradation, their therapeutic utility is constrained by two inherent limitations: (i) dependence on the ubiquitin‐proteasome system (UPS), which exhibits reduced efficacy against membrane‐associated and vesicularly trafficked proteins due to compartmental biases favoring nuclear/cytosolic substrates, (ii) vulnerability to contextual variability in E3 ligase expression and subcellular localization across cell types and pathological states, and (iii) hydrophobic‐tag‐based degradation strategies—which induce target degradation by simulating protein misfolding—suffer from limited efficiency and a narrow scope of actionable targets [24, 25]. In this context, autophagy‐tethering chimera (ATTECs), which leverage the autophagic pathway to degrade a broad spectrum of substrates, have garnered increasing interest [26, 27]. ATTECs are chimeric molecules consisting of three modules: a ligand for the target protein, a linker, and an LC3‐binding moiety. By physically tethering the protein of interest (POI) to LC3—a key autophagy protein—ATTECs facilitate POI degradation via the autophagosome‐lysosome pathway [28]. In contrast to PROTACs, ATTECs operate independently of ubiquitination, representing a more direct degradation strategy [29]. Notably, ATTECs are capable of degrading not only proteins but also non‐protein biomolecules such as lipids [30, 31]. Given that STING is physiologically cleared via the ESCRT pathway, the ATTEC strategy provides a biomimetic means to redirect STING toward lysosomal degradation. However, despite this conceptual alignment, STING‐directed ATTECs have not yet been reported.

**SCHEME 1:**
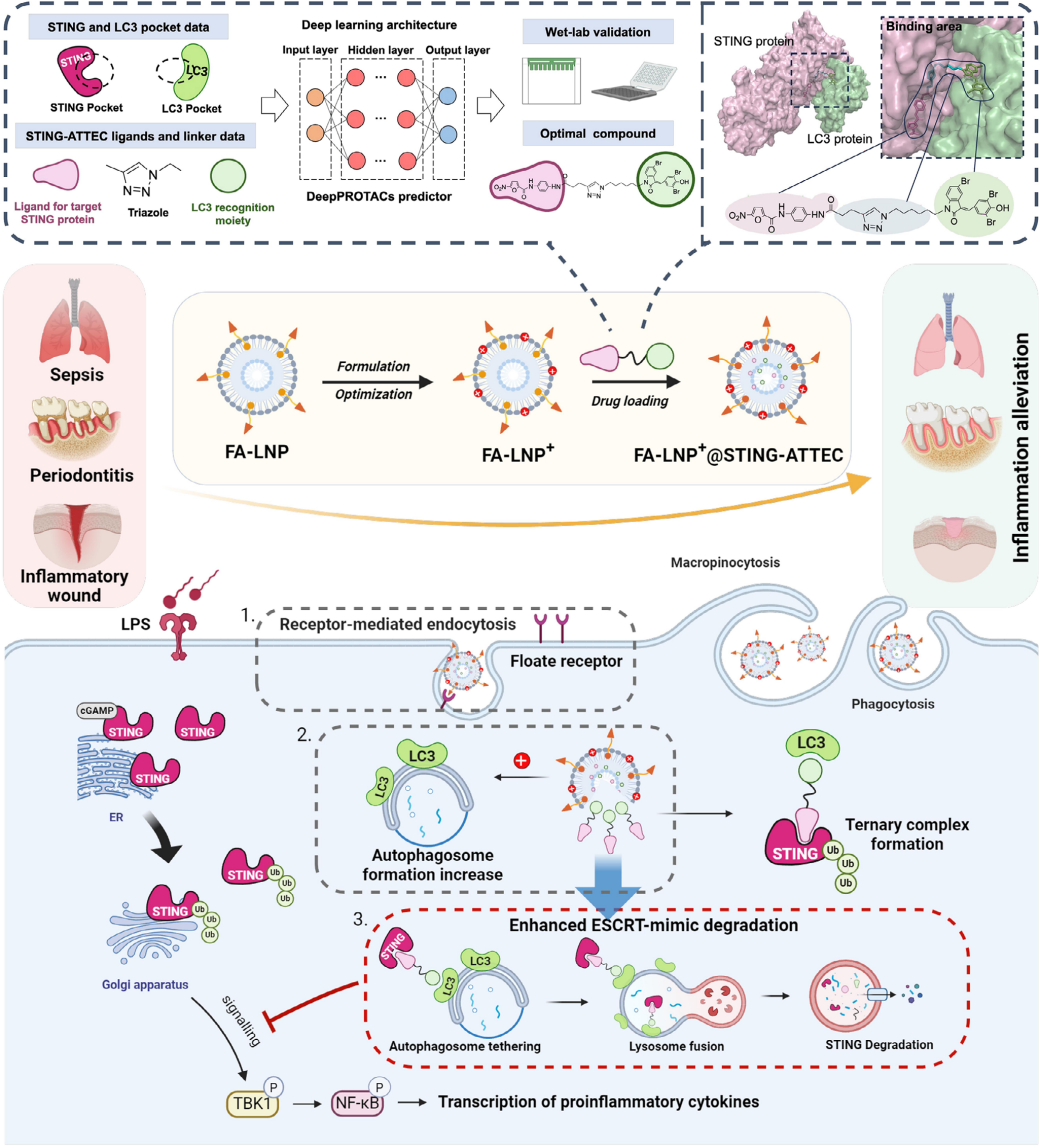
Schematic illustration of the rational design and therapeutic mechanism of FA‐LNP^+^@STING‐ATTEC for targeted STING degradation in inflammatory diseases. A STING‐directed c‐targeting chimera (STING‐ATTEC) was designed using a structure‐guided strategy, linking the STING‐binding molecule C170 to the LC3‐interacting compound GW5074 through diverse chemical linkers. The triazole‐linked construct demonstrated superior degradation efficiency, as predicted by *DeepPROTACs* computational modeling and validated via Western blot analysis. The optimized STING‐ATTEC was encapsulated into folate‐modified cationic lipid nanoparticles (FA‐LNP^+^), engineered to enhance macrophage‐specific targeting through folate receptor mediation and potentiate autophagic activity via DOTAP incorporation. This integrated nanoplatform facilitates targeted STING degradation through biomimetic activation of the autophagy–lysosome pathway, effectively attenuating inflammatory signaling and promoting tissue homeostasis in multiple disease models.

Herein, for the first time, we designed STING‐directed autophagy‐targeting chimeras (STING‐ATTECs), inspired by the physiological ESCRT‐mediated lysosomal degradation pathway. Deep learning‐guided molecular optimization to achieve potent and selective degradation of the STING protein. The degradation efficacy of these computationally engineered STING‐ATTECs was rigorously validated across both in vitro cellular assays and in vivo inflammatory models. To further improve macrophage delivery and enhance autophagic activity, the STING‐ATTEC compounds were encapsulated within DOTAP‐containing lipid nanoparticles (LNPs), exploiting the cationic lipid's dual capacity to facilitate intracellular uptake and intrinsically induce autophagosome formation. The resulting nanoplatform not only facilitated efficient cytosolic delivery but also synergistically augmented STING degradation through increased autophagic clearance, markedly attenuating inflammatory responses. Together, these efforts yield a rational nanodegrader system that directs STING to autolysosomal degradation, mirroring its natural turnover pathway while overcoming limitations of UPS‐based approaches. By sequentially combining deep learning‐accelerated degrader design with a pro‐autophagic nanoencapsulation strategy, this work provides a transformative platform for targeted degradation of membrane proteins and opens new avenues for precise immunomodulatory therapy (Scheme 1).

## 2 Results

### 2.1 STING Correlates with Inflammatory Diseases in Patients

**FIGURE 1:**
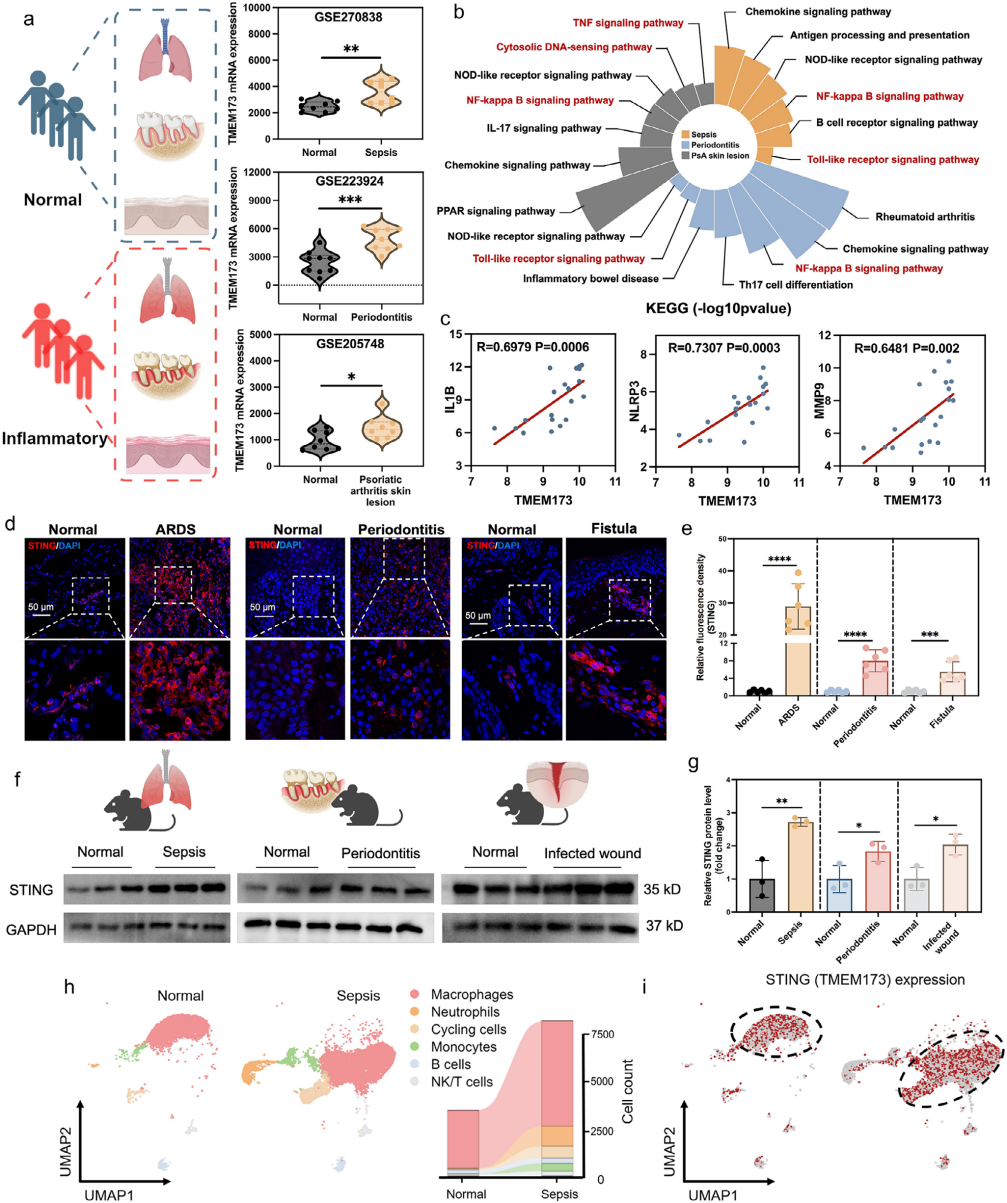
STING is upregulated in inflammatory diseases and enriched in macrophages. (a) The expression of TMEM173 in the samples of patients diagnosed with sepsis, periodontitis, and psoriatic skin lesions from datasets GSE270838, GSE223924, and GSE205748. (b) KEGG pathway analysis of DEGs from datasets GSE270838, GSE223924, and GSE205748. (c) The correlations between the expression of TMEM173 and IL‐1β, NLRP3, and MMP9 from dataset GSE205748. (d,e) Comparative immunofluorescence analysis of STING protein expression in clinical samples (ARDS lung tissues, periodontal granulation tissue, and skin fistula tissues) vs. normal control tissues, with semi‐quantitative assessment (n = 6, per group). (f,g) Western blot analysis and quantitative assessment of STING protein expression in tissues from mouse models of sepsis, periodontitis, and inflammatory skin wound healing (n = 3 per group). (h) UMAP analysis and corresponding alluvial diagram of immune cell populations in the normal and sepsis groups from the GSE190856 dataset. (i) UMAP visualization of STING expression in all clusters. Statistical significance: *p < 0.05, **p < 0.01, ***p < 0.001, ****p < 0.0001.

To investigate the potential role of STING in inflammatory pathogenesis, we analyzed transcriptomic data from three independent RNA‐seq datasets representing distinct human inflammatory conditions: sepsis‐induced lung injury (GSE270838), periodontitis (GSE223924), and psoriatic skin lesions (GSE205748) [32, 33, 34]. Notably, *TMEM173* (encoding STING) exhibited consistently elevated mRNA expression in diseased tissues compared to healthy controls (Figure 1a), underscoring its widespread significance in the pathogenesis of inflammatory disorders. Pathway enrichment analysis of upregulated genes across these datasets revealed marked activation of key inflammatory signaling cascades—including the NF‐κB pathway, cytosolic DNA‐sensing pathway, TNF signaling, and Toll‐like receptor (TLR) pathways—all of which were consistently and significantly enriched (Figure 1b). Further supporting this connection, correlation analysis (Figure 1c) identified a strong positive association between *TMEM173* expression and pro‐inflammatory mediators such as IL‐1β, NLRP3, and MMP9. Together, these findings suggest that elevated STING expression may drive both innate immune hyperactivation and inflammatory tissue damage through these effector molecules.

We next sought to validate these findings at the protein level using immunofluorescence staining of clinical specimens. Due to the limited availability of matched public datasets and clinical specimens for exactly the same disease entities, clinical validation was performed using samples from acute respiratory distress syndrome (ARDS), periodontitis, and chronic skin fistulas, which represent comparable inflammatory contexts. Notably, all three disease tissues exhibited markedly enhanced STING fluorescence signals compared to healthy counterparts (Figure 1d). Semi‐quantitative analysis confirmed a significant increase in STING protein levels under each pathological condition (Figure 1e), corroborating the transcriptomic data and supporting a role for STING protein in the pathology of inflammatory lesions.

To further evaluate STING protein expression under inflammatory conditions, we assessed STING protein levels in mouse models of sepsis, periodontitis, and inflammatory skin wound healing. Mirroring our clinical observations, inflamed tissues from each model showed elevated STING levels compared to healthy controls (Figure 1f). Densitometric quantification confirmed consistent upregulation of STING protein across all three pathological contexts (Figure 1g), underscoring the translational relevance of our human tissue findings and establishing STING hyperactivation as a conserved hallmark of inflammation from bedside to bench.

To pinpoint the cellular source of STING within the inflammatory milieu, we analyzed single‐cell RNA sequencing data (GSE190856) from the sepsis context [35]. Among CD45^+^ leukocytes, macrophages emerged as the predominant immune subset, significantly outnumbering neutrophils, cycling progenitors, monocytes, B cells, and T/NK cells (Figure 1h). Notably, macrophage abundance increased markedly following induction of sepsis, highlighting their pivotal role in the inflammatory cascade and positioning them as a prime cellular target for potential STING‐directed therapeutic strategies. Consistently, UMAP visualization of STING expression across all clusters (Figure 1i) revealed that macrophages exhibited the highest STING levels compared with other immune cell types. This observation was further corroborated by immunofluorescence co‐staining of STING and the macrophage marker F4/80 (Figure S1), solidifying macrophages as the dominant STING‐expressing population under inflammatory conditions.

### 2.2 Inhibition of STING Alleviates Inflammation and Disease Progression

**FIGURE 2:**
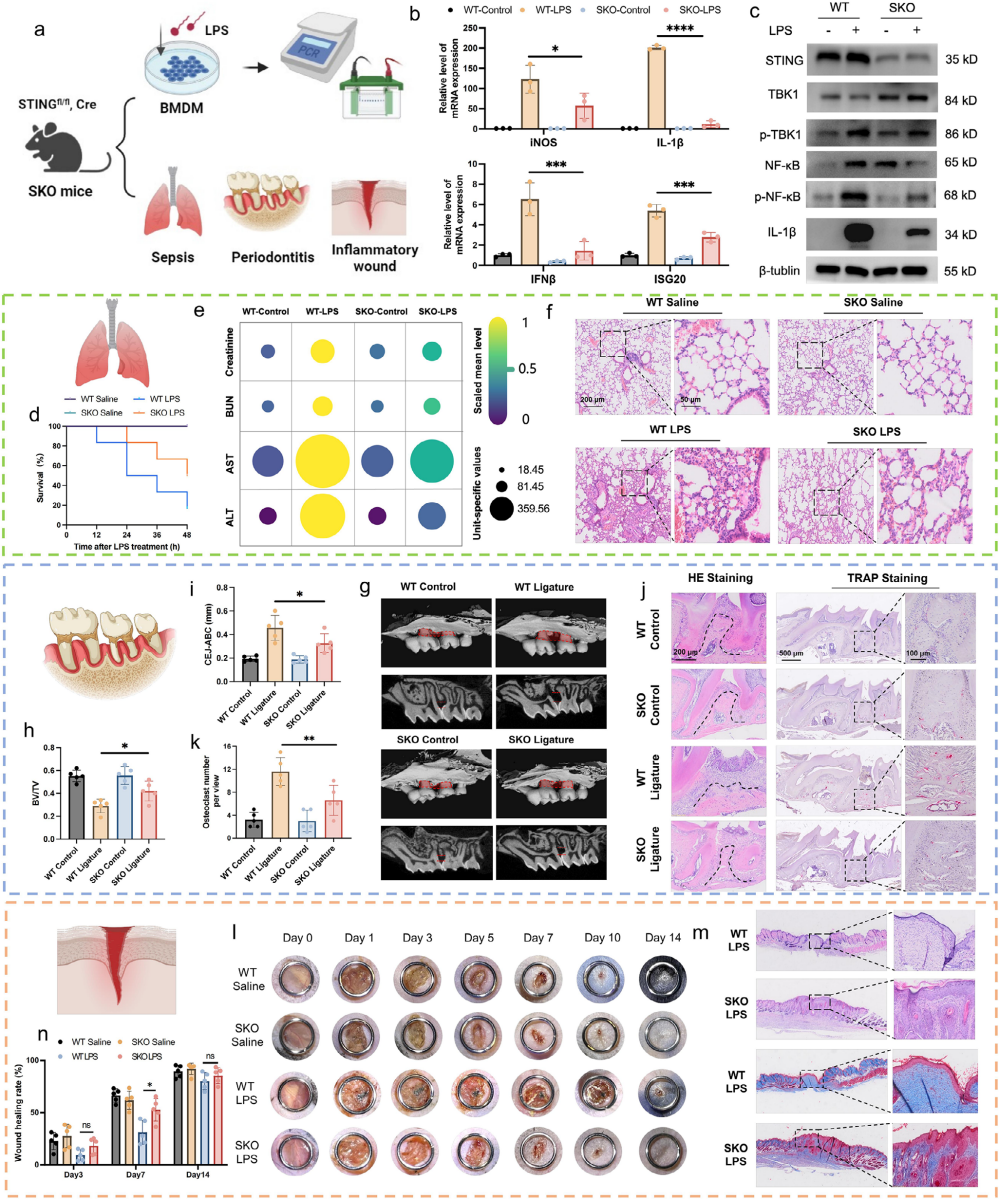
STING inhibition attenuates inflammatory disease phenotypes. (a) Schematic diagram of STING knockout (Cre/loxP‐mediated in mice) and subsequent isolation of BMDMs for in vitro experiments, as well as the use of STING‐KO mice in sepsis, periodontitis, and inflammatory wound models for in vivo analysis. (b) qRT‐PCR analysis of IFNβ, ISG20, IL‐1β, and iNOS mRNA levels in BMDMs isolated from STING‐KO mice (n = 3, per group). (c) Western blot analysis showing protein expression of TBK1, p‐TBK1, NF‐κB, p‐NF‐κB, and IL‐1β in STING‐KO‐derived BMDMs. (d) Survival curves of WT and STING‐ KO mice after LPS‐induced septic shock. (e) Heatmap of serum biochemical markers (creatinine, BUN, AST, ALT) in WT and STING‐KO mice post‐LPS challenge. (f) H&E staining of lung tissues from LPS‐treated WT and STING‐KO mice. (g) Micro‐CT images of alveolar bone resorption in WT and STING‐KO mice with ligature‐induced periodontitis. (h) Quantification of bone volume/total volume (BV/TV) in ligature‐treated mice (n = 5, per group). (i) Measurement of cementoenamel junction (CEJ) to alveolar bone crest (ABC) distance (n = 5, per group). (j) H&E and TRAP staining of alveolar bone in ligature‐induced periodontitis models. (k) Quantification of TRAP‐positive osteoclasts in alveolar bone (n = 5, per group). (l) Macroscopic images of skin wound healing over 14 days in WT and STING‐KO mice. (m) H&E and Masson's trichrome staining of skin tissues during wound healing. (n) Quantitative data of the relative wound area at different time points (n = 5, per group). Statistical significance: *p < 0.05, **p < 0.01, ***p < 0.001, ****p < 0.0001.

To functionally assess the crucial role of STING under inflammatory conditions, we combined ex vivo analyses of BMDMs isolated from STING‐knockout (SKO) mice with in vivo knockout models (Figure 2a). In STING‐deficient BMDMs, LPS stimulation resulted in markedly attenuated induction of interferon‐stimulated genes (e.g., IFNβ and ISG20) and pro‐inflammatory mediators (including IL‐1β and iNOS) at the mRNA level (Figure 2b). This was corroborated at the protein level by reduced phosphorylation of TBK1 and NF‐κB p65, along with diminished IL‐1β secretion (Figure 2c), confirming the indispensable role of STING in orchestrating core inflammatory signaling.

Notably, SKO mice exhibited profound survival benefits in the LPS‐induced sepsis model (Figure 2d), accompanied by reduced serum levels of organ injury markers (creatinine, BUN, AST, ALT; Figure 2e). Histological examination revealed substantial protection against LPS‐induced lung injury, including attenuated alveolar wall thickening, edema, and inflammatory infiltration (Figure 2f). Beyond acute inflammation, SKO mice were also protected against inflammation‐driven bone loss in an experimental periodontitis model. Micro‐CT analysis unveiled robust preservation of alveolar bone volume (BV/TV) and architecture, cementoenamel junction to alveolar bone crest (CEJ–ABC) distance vs. wild‐type controls (Figure 2g–i). Histological staining of periodontal tissues further strengthened this observation, with Hematoxylin and eosin (H&E) staining showing reduced inflammatory infiltration and TRAP staining indicating fewer osteoclasts in SKO gingival tissues (Figure 2j,k), suggesting suppressed osteoclastogenesis under inflammatory conditions. Furthermore, STING deficiency promoted tissue repair in an inflammatory skin wound model. Macroscopic imaging revealed visibly expedited wound closure over 14 days in SKO mice (Figure 2l), while histological analysis confirmed enhanced re‐epithelialization and collagen deposition (Figure 2m). Quantitative evaluation revealed significantly improved wound resolution (Figure 2n), indicating that STING block may facilitate healing by reducing inflammation and promoting matrix remodeling.

Together, these results establish STING as a master regulator of pathological inflammation across multiple tissue types, and suggest its targeted inhibition may offer a promising therapeutic strategy not only to mitigate tissue damage but also to foster regeneration in inflammatory disease settings.

### 2.3 STING Undergoes ESCRT‐Mediated Degradation Following Activation in Inflammatory Macrophages

**FIGURE 3:**
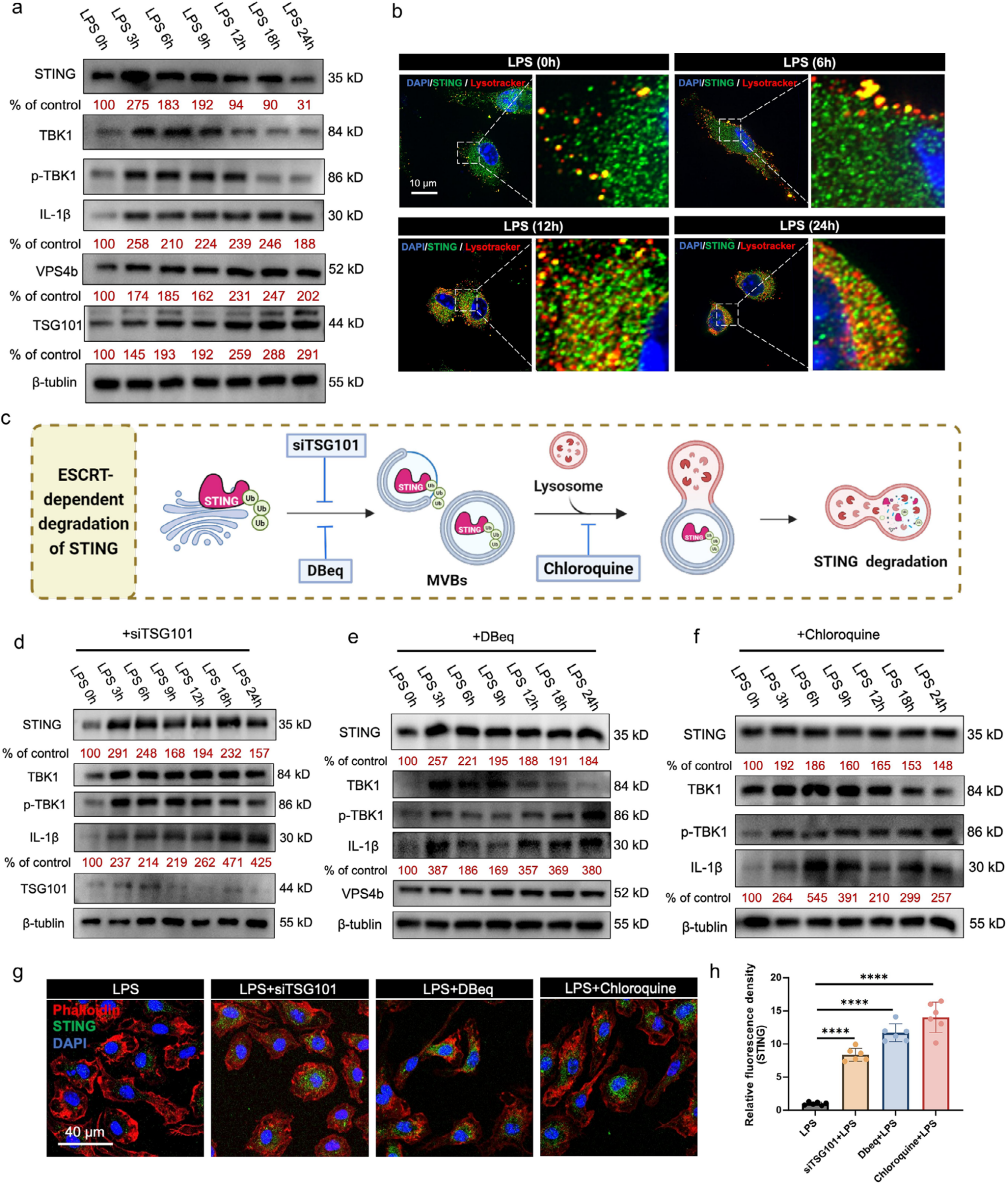
ESCRT‐mediated degradation pathway of STING and its regulatory mechanisms in inflammation. (a) Western blot analysis of STING, TBK1, p‐TBK1, IL‐1β, VPS4b, and TSG101 protein levels in BMDMs at different time points following LPS stimulation. (b) Representative immunofluorescence images of STING and Lysotracker in BMDMs at given time points following LPS stimulation. (c) Schematic illustration of the ESCRT‐dependent degradation mechanism of STING. (d–f) Western blot analysis of STING, TBK1, p‐TBK1, and IL‐1β protein levels in BMDMs following LPS stimulation and treatment with si‐TSG101 (d), DBeq (e), or chloroquine (f); TSG101 and VPS4b levels were additionally assessed in si‐TSG101‐ and DBeq‐treated groups, respectively. (g,h) Immunofluorescence images and corresponding quantification of STING expression in BMDMs after 24 h of LPS stimulation under different treatments (n = 6, per group). Statistical significance: *p < 0.05, **p < 0.01, ***p < 0.001, ****p < 0.0001.

In inflammatory macrophages, STING degradation following activation is mediated by the ESCRT machinery and subject to spatiotemporal control via organellar trafficking [15]. Western blot analysis unveiled a time‐dependent biphasic regulation: STING protein levels increased at 3–9 h post‐stimulation, concurrent with TBK1 phosphorylation and IL‐1β upregulation, and subsequently declined from 12–24 h, coinciding with elevated expression of ESCRT components, including TSG101 and VPS4b (Figure 3a). The persistence of IL‐1β during the degradation phase implies impaired ESCRT‐mediated clearance under inflammatory conditions, leading to prolonged downstream signaling. Immunofluorescence analysis corroborated these dynamics, showing peak STING expression at 6 h post‐LPS stimulation and subsequent attenuation at 12 and 24 h, accompanied by increased lysosomal colocalization (Figure 3b; Figure S2). Critically, both genetic (TSG101 knockdown) and pharmacological disruption (DBeQ‐mediated ESCRT inhibition; chloroquine‐induced lysosomal neutralization) abrogated late‐phase STING degradation, resulting in sustained TBK1 activation and elevated IL‐1β expression (Figure 3c–f). Consistent with these observations, immunofluorescence at 24 h post‐LPS revealed intensified STING signal across all intervention groups compared to LPS treatment alone (Figure 3g,h). Collectively, these findings demonstrate that efficient ESCRT‐mediated lysosomal degradation of STING is essential for the resolution of inflammation, and that compromised ESCRT function under inflammatory stress sustains innate immune activation. Therefore, strategies designed to enhance ESCRT activity represent a biomimetic and therapeutically promising approach to restore timely STING clearance and mitigate pathological inflammation.

### 2.4 Deep Learning‐Guided Design and Mechanistic Validation of an Autophagy‐Lysosome‐Targeted STING Degrader

**FIGURE 4:**
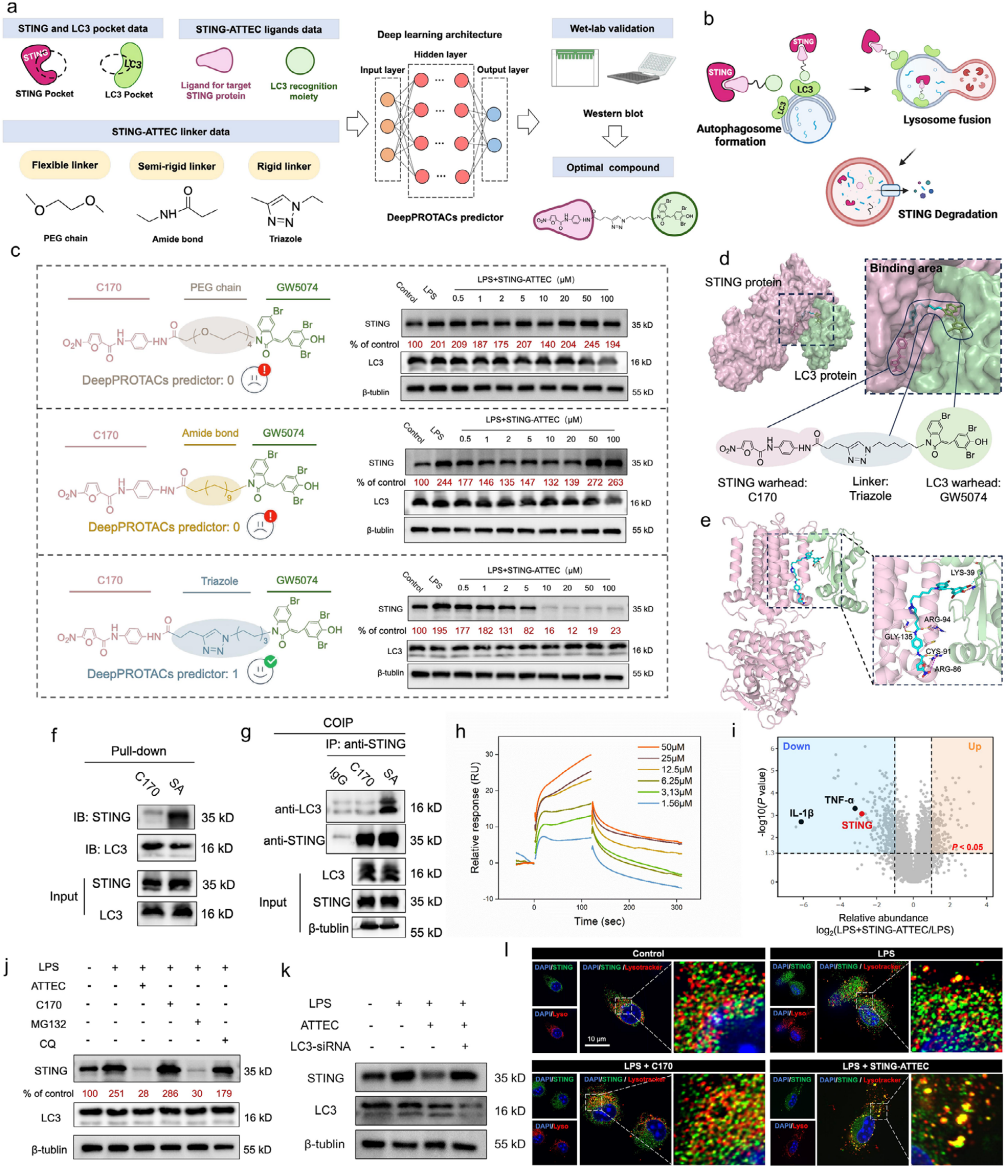
Computational design and mechanistic validation of an autophagy‐lysosome‐targeting STING degrader (STING‐ATTEC). (a) Schematic workflow for STING‐ATTEC design based on *DeepPROTACs Predictor*, using fixed STING and LC3 pocket/ligand data and varying linker types (PEG, amide bond, and triazole). (b) Proposed mechanism of STING degradation via autophagosome tethering and lysosomal fusion mediated by STING‐ATTEC. (c) Chemical structures of three STING‐ATTEC variants with their corresponding *DeepPROTACs predictor* scores (0 = poor degradation potential, DC_50_ > 100 nM, Dmax < 80%; 1 = favorable degradation potential, DC_50_ ≤ 100 nM, Dmax ≥ 80%). Western blot analysis under LPS stimulation further validated the differential degradation efficacy of these variants. (d) Molecular docking of optimized STING‐ATTEC with STING and LC3 proteins; magnified view shows compound structure. (e) Structural mapping of STING and LC3 proteins and key amino acid residues potentially mediating interactions with the optimized STING‐ATTEC compound. (f) In vitro pull‐down assay. (g) Co‐immunoprecipitation assay in BMDMs. (h) SPR sensorgrams showing concentration‐dependent binding of STING‐ATTEC to recombinant STING protein (1.56–50 µM). (i) Volcano plot of proteomic profiling comparing LPS+STING‐ATTEC vs. LPS. (j) Western blot analysis of STING and LC3 levels under lysosomal or proteasomal inhibition. (k) Western blot showing STING and LC3 levels after LC3 knockdown. (l) Immunofluorescence staining of STING and lysosomes in BMDMs under the indicated treatments after LPS stimulation for 6 h.

Impaired ESCRT functionality is mechanistically linked to the pathogenesis of diverse inflammatory disorders, highlighting the therapeutic potential of strategies that restore or emulate this endogenous degradation pathway. Inspired by the physiological ESCRT‐mediated turnover of STING, we designed a structured degrader molecule, STING‐ATTEC, that binds specific STING motifs and redirects the protein toward lysosomal clearance. This rational design incorporates dual warheads targeting STING and LC3: C170, a validated STING‐binding agent retaining downstream degradation capacity, and GW5074 conjugated to LC3 without compromising overall autophagy function [20, 36, 37]. These pharmacophores are conjugated via a linker to form the final degrader. The linker length was initially selected based on structural considerations to allow simultaneous engagement of the target protein and LC3, followed by empirical optimization through synthesis and evaluation of several linker variants. Using these anchors, three linker chemotypes—a flexible PEG chain, a semi‐rigid amide bond, and a rigid triazole ring—were evaluated for their degradation potential via the *DeepPROTACs Predictor* (Figure 4a,b) [38]. The *DeepPROTACs predictor*, originally developed for PROTAC systems, was used here only as a preliminary computational reference to explore potential structural features associated with degradation efficiency. Notably, while PEG and amide linkers showed no predicted degradation activity (score = 0), the triazole‐linked construct achieved a degradation score of 1.0 (Figure 4a,c), indicating a high probability of STING degradation via this linkage. To validate *DeepPROTACs* predictions, we synthesized three structurally distinct STING‐ATTEC variants with different linkers. Western blot analysis confirmed that only the triazole‐linked variant induced dose‐dependent STING degradation in LPS‐stimulated macrophages (Figure 4c), consistent with computational modeling. Based on these findings, the triazole‐linked STING‐ATTEC was advanced to downstream mechanistic investigations. To determine whether STING‐ATTEC globally affects autophagic flux, the levels of LC3‐I/II were also examined. No significant changes were observed following treatment, suggesting that STING‐ATTEC does not induce general autophagy activation but instead promotes selective degradation of STING.

To elucidate the structural basis by which the optimized STING‐ATTEC facilitates ternary complex formation, we first docked STING and LC3 using ZDOCK, which revealed stable protein–protein interactions mediated by multiple hydrogen bonds, including those between LC3 residues Gln116, Glu102, Ser101, Glu97 and STING residues Tyr104(A), Arg94(A), Arg86, and Trp82 (Figure S4). Subsequent induced‐fit docking of the small molecule into the pre‐assembled STING–LC3 complex demonstrated a binding mode consistent with reported STING warhead orientations: the nitro group and adjacent carbonyl formed a salt bridge and hydrogen bond with Arg86(A), while the central linker established hydrogen bonds with Arg94(A) and Gly135(B) (Figure 4d,e; Figure S5). The saturated alkyl linker further engaged in extensive hydrophobic contacts with STING residues Leu51(A), Leu98(A), Leu130(B), and LC3 Val124. On the LC3‐facing end, the brominated indole moiety of the LC3 warhead was inserted into a hydrophobic pocket formed by STING Leu101(A), Phe105(A) and LC3 Leu82, Phe119, Leu122, and Val124, while the terminal phenol engaged in hydrogen bonding, salt bridge, and halogen bonding interactions with LC3 Lys39. This cooperative interaction pattern, in which both warhead modules are anchored by complementary electrostatic and hydrophobic contacts, resulted in a highly favorable docking score of –11.257, underscoring the strong theoretical basis for efficient ternary complex stabilization.

To investigate whether STING‐ATTEC promotes the formation of a ternary complex between STING and LC3, we first performed in vitro pull‐down assays using purified His‐LC3B protein (Figure 4f). Compared with the control compound C170, STING‐ATTEC markedly increased the amount of STING protein pulled down by His‐LC3B, indicating that the compound enhances the interaction between STING and LC3 in vitro. To further verify this interaction in cells, co‐immunoprecipitation (Co‐IP) experiments were conducted using endogenous proteins (Figure 4g). Immunoprecipitation with anti‐STING antibodies revealed that LC3 association with STING was significantly increased in cells treated with STING‐ATTEC compared with the C170 control. These findings are consistent with the proposed mechanism that STING‐ATTEC acts as a molecular bridge to recruit LC3 to STING, thereby facilitating the formation of a STING–ATTEC–LC3 ternary complex and promoting autophagy‐dependent degradation of STING.

Surface plasmon resonance (SPR) analysis further confirmed the direct interaction between STING‐ATTEC and the STING protein. The sensorgrams exhibited a clear concentration‐dependent binding response, yielding a dissociation constant (KD) of 1.78 × 10^−^^5^ M (Figure 4h). Notably, a slightly larger response increase was observed when the ligand concentration increased from 25 to 50 µM compared with the increase from 12.5 to 25 µM. This deviation from an ideal 1:1 binding trend may be attributed to weak non‐specific interactions with the sensor surface or minor aggregation of the small‐molecule ligand at higher concentrations, phenomena occasionally observed in SPR measurements of small molecules. Nevertheless, the overall dose‐responsive binding profile and reproducible sensorgrams support the specific interaction between STING‐ATTEC and the STING protein. In addition, circular dichroism (CD) spectroscopy revealed that the presence of STING‐ATTEC induced conformational alterations in the STING protein, suggesting changes in its secondary structure (Figure S6). Collectively, these results provide biophysical evidence supporting the direct binding of STING‐ATTEC to STING.

Next, we investigated the temporal dynamics of STING‐ATTEC–mediated degradation. A marked reduction in STING protein was observed as early as 6 h post‐treatment (Figure S7). Control experiments further demonstrated that the individual ligands (C170 and GW5074) exerted no effect on STING protein abundance (Figure S8). In addition, STING‐ATTEC treatment did not alter TMEM173 mRNA levels, supporting that its activity specifically occurs at the protein level (Figure S9). The biocompatibility of STING‐ATTEC was further evaluated using CCK8 assays, which revealed no appreciable cytotoxicity in RAW264.7 and L929 cells across a concentration range of 1–50 µM (Figure S10). Proteomic profiling revealed broad changes in the inflammatory proteome upon STING‐ATTEC treatment, with STING among the most significantly downregulated proteins, accompanied by marked reductions in other key inflammatory mediators such as TNFα and IL‐1β (Figure 4i). To determine the degradation route, lysosomal and proteasomal inhibitors were employed. While chloroquine (a lysosomal inhibitor) effectively blocked STING‐ATTEC–mediated degradation, MG132 (a proteasome inhibitor) had no effect, confirming a lysosome‐dependent degradation mechanism (Figure 4j). Furthermore, to investigate whether ubiquitination is required for this process, cells were treated with TAK‐243, a selective inhibitor of the ubiquitin‐activating enzyme E1 that blocks cellular ubiquitination (Figure S11). Notably, inhibition of E1 did not impair STING degradation induced by STING‐ATTEC, indicating that the degradation process occurs independently of ubiquitination. These results further support that STING‐ATTEC promotes STING degradation through a ubiquitin‐independent, lysosome‐dependent pathway. Furthermore, LC3 knockdown via siRNA attenuated STING degradation, establishing the necessity of autophagosomal engagement (Figure 4k). Finally, immunofluorescence imaging indicated that STING‐ATTEC not only reduced STING accumulation in LPS‐stimulated cells but also enhanced its colocalization with lysosomal compartments, further supporting autophagy‐lysosomal targeting (Figure 4l; Figure S12). Together, these data establish STING‐ATTEC as a rationally designed, autophagy‐lysosome‐dependent degrader that effectively eliminates STING under inflammatory conditions, functionally recapitulating ESCRT‐mediated clearance.

### 2.5 FA‐LNP^+^@STING‐ATTEC Facilitates LC3‐Dependent Autophagy to Amplify STING Clearance and Suppress Inflammatory Responses

**FIGURE 5:**
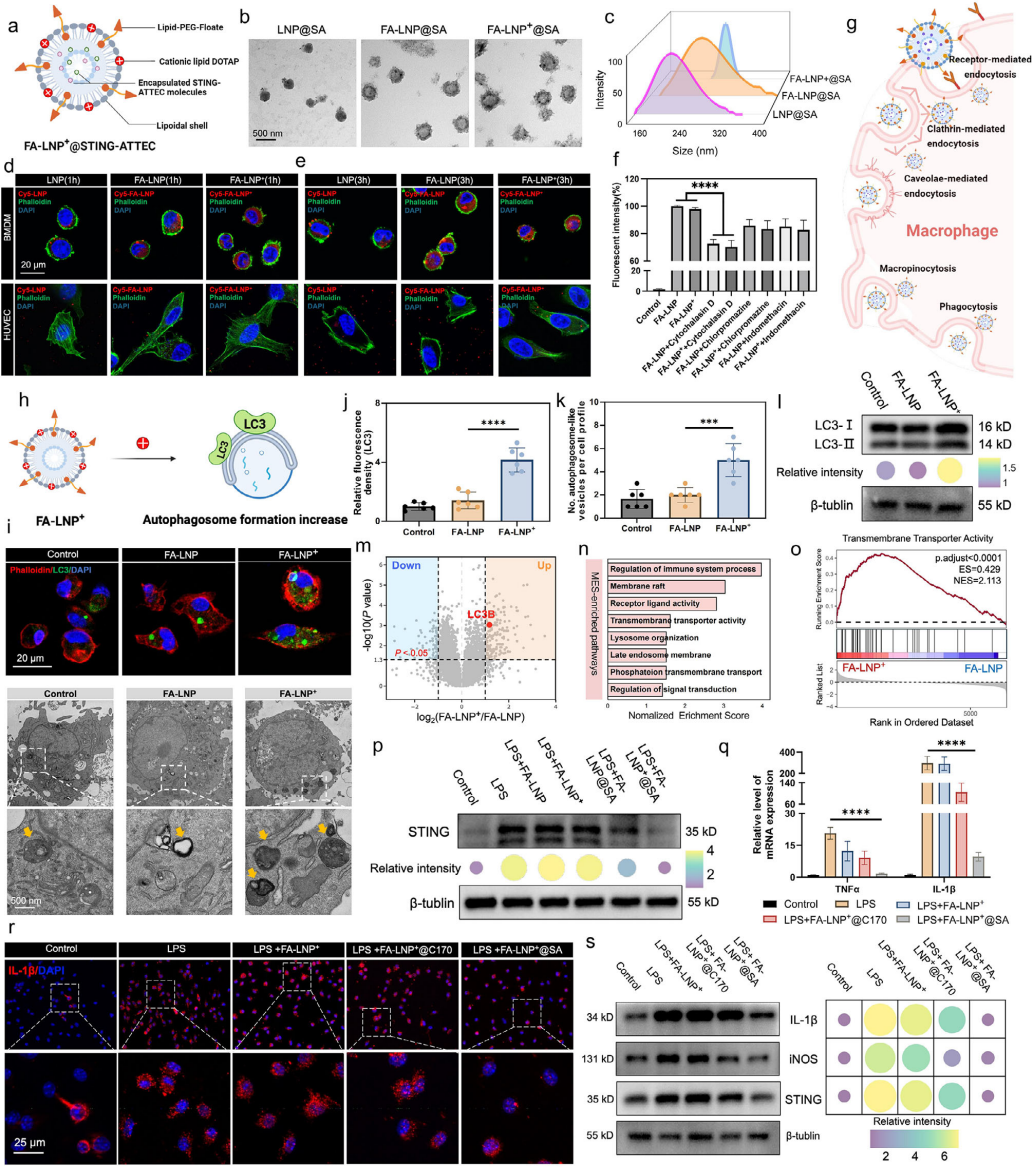
FA‐LNP^+^@STING‐ATTEC promotes LC3 expression and autophagic activity to synergistically enhance STING protein degradation and anti‐inflammatory effects. (a) Schematic diagram of FA‐LNP^+^@STING‐ATTEC composition and structure. (b) TEM image of nanoparticles. (c) Size distribution of nanoparticles measured by DLS. (d,e) Cellular uptake of Cy5‐labeled LNP and FA‐LNP in BMDMs and HUVECs by immunofluorescence. (f) Flow cytometry quantification of FA‐LNP and FA‐LNP^+^ uptake in cells treated with different endocytosis inhibitors (n = 3, per group). (g) Proposed endocytic pathways for FA‐LNP internalization in macrophages. (h) Schematic illustration of FA‐LNP^+^‐enhanced autophagosome formation. (i) Images of LC3 immunofluorescence staining (green puncta) and biological TEM (yellow arrows) illustrating autophagosome formation in the indicated groups after 3 h incubation with the nanoparticles. (j,k) Quantification of LC3 puncta (j) and autophagosomes (k) from (i) (n = 6, per group). (l) Western blot analysis of LC3 protein expression. (m) Volcano plot of proteomic profiling comparing FA‐LNP^+^ vs. FA‐LNP. (n) GEO analysis showing enriched pathways in the FA‐LNP^+^ group compared with the FA‐LNP group. (o) GSEA analysis shows transmembrane transporter activity pathway enrichment in the FA‐LNP^+^ group. (p) Western blot analysis of STING protein expression under the indicated treatments. (q) qRT‐PCR analysis of IL‐1β and TNF‐α mRNA levels under different treatments (n = 3, per group). (r) Immunofluorescence staining of IL‐1β expression. (s) Western blot analysis of STING, iNOS, and IL‐1β protein expression. Statistical significance: *p < 0.05, **p < 0.01, ***p < 0.001, ****p < 0.0001.

While ATTECs represent a promising strategy for TPD via autophagy, their clinical translation has been limited by pharmacological challenges such as poor membrane permeability, low bioavailability, and potential off‐target effects [27, 39]. To overcome these limitations, we engineered a folate‐modified cationic lipid nanoparticle (FA‐LNP^+^) for macrophage‐targeted delivery of STING‐ATTEC, aiming to enable both macrophage‐selective uptake and enhanced autophagosome formation through surface charge modulation [21, 40, 41]. As schematized in Figure 5a, FA‐LNP^+^@STING‐ATTEC integrates three functional components: (1) a lipid bilayer encapsulating the STING‐ATTEC molecule, (2) FA as a macrophage‐targeting ligand, and (3) the incorporation of DOTAP, a cationic lipid, to shift the surface charge from mildly negative to positively charged, thereby enhancing intracellular interactions related to autophagy activation. Transmission electron microscopy (TEM) revealed well‐dispersed, spherical particles across all tested formulations (Figure 5b). Among them, FA‐LNP^+^@STING‐ATTEC exhibited the largest particle size (∼320 nm), followed by FA‐LNP@STING‐ATTEC (∼300 nm) and unmodified LNP@STING‐ATTEC (∼210 nm), indicating structural modulation by FA conjugation and DOTAP enrichment. Consistently, dynamic light scattering (DLS) analysis confirmed the increased hydrodynamic diameter of the FA‐modified and charge‐engineered formulations. All nanoparticles exhibited relatively low PDI values (0.17 for LNP@STING‐ATTEC, 0.20 for FA‐LNP@STING‐ATTEC, and 0.11 for FA‐LNP^+^@STING‐ATTEC), indicating acceptable uniformity for lipid nanoparticle formulations (Figure 5c; Figure S13).

The drug encapsulation efficiency and drug loading capacity of the three nanoparticle formulations were first quantified. As shown in Figure S14, the encapsulation efficiency of LNP@STING‐ATTEC, FA‐LNP@STING‐ATTEC, and FA‐LNP^+^@STING‐ATTEC was 84.0%, 81.1%, and 76.2%, respectively, indicating that STING‐ATTEC could be efficiently incorporated into all nanoparticle formulations. The corresponding drug loading capacities were 6.49%, 6.31%, and 5.52%, respectively, suggesting that folic acid modification and the introduction of cationic lipids had minimal impact on the loading efficiency of STING‐ATTEC.

The release behavior of FA‐LNP^+^@STING‐ATTEC was further investigated under different pH conditions to mimic physiological and intracellular environments. Specifically, pH 7.4 represents the physiological condition of blood circulation, pH 6.5 mimics the mildly acidic inflammatory microenvironment, and pH 5.5 reflects the acidic conditions of endosomes and lysosomes after cellular internalization. As shown in Figure S15, the cumulative release of STING‐ATTEC after 24 h was 13.4% at pH 7.4, 36.3% at pH 6.5, and 69.9% at pH 5.5, indicating a pronounced pH‐responsive release behavior. The relatively low release at physiological pH suggests good nanoparticle stability during circulation, while the significantly enhanced release under acidic conditions is favorable for intracellular drug release following endosomal/lysosomal trafficking.

To further evaluate the physicochemical stability of FA‐LNP^+^ nanoparticles under physiological‐like conditions, their hydrodynamic size and PDI were monitored over 48 h in PBS and 10% FBS. FA‐LNP^+^ nanoparticles exhibited good colloidal stability in PBS, with minimal changes in particle size and PDI during the 48 h incubation period (Figure S16). In contrast, a slight increase in particle size was observed in 10% FBS, where the average diameter increased from approximately 317 to 344 nm after 48 h, likely due to interactions between serum proteins and the positively charged nanoparticle surface. Despite this modest increase, the nanoparticles maintained a relatively narrow size distribution. In addition, the stability of drug encapsulation was evaluated over the same period (Figure S17). The encapsulation efficiency gradually decreased from 76.2% to 59.7% after 48 h incubation, suggesting a moderate level of drug release under physiological conditions. Together, these results indicate that FA‐LNP^+^ nanoparticles maintain acceptable colloidal stability and drug retention within 48 h in physiologically relevant environments.

To evaluate cell‐type selectivity, Cy5‐labeled LNP and FA‐LNP were incubated with BMDMs and HUVECs. BMDMs were chosen as representative inflammatory macrophages that play key roles in innate immune responses and express folate receptors associated with macrophage targeting. In contrast, HUVECs were used as a non‐immune endothelial control cell type with relatively low folate receptor expression to assess non‐specific nanoparticle uptake. Immunofluorescence analysis (Figure 5d,e) demonstrated that LNP formulations were internalized by BMDMs, reflecting the innate phagocytic capacity of macrophages toward lipid‐based nanoparticles. FA‐LNP and FA‐LNP^+^ exhibited significantly enhanced uptake in BMDMs at 3 h, indicating folate receptor‐mediated endocytosis. In contrast, HUVECs, which express low levels of folate receptor, exhibited minimal nanoparticle uptake regardless of FA modification, confirming the macrophage‐targeting specificity conferred by folate functionalization. Flow cytometry revealed significant inhibition of Cy5‐labeled FA‐LNP and FA‐LNP^+^ internalization by multiple pharmacological agents, indicating involvement of diverse active transport mechanisms (Figure 5f,g; Figure S18). Cytochalasin D, which disrupts actin polymerization and receptor‐mediated endocytosis, produced the strongest inhibition. Other inhibitors targeting clathrin‐mediated (chlorpromazine, caveolar (indomethacin), macropinocytic (colchicine), and phagocytic pathways (wortmannin) showed significant but milder effects [42, 43, 44]. Collectively, these data indicate that folate‐conjugated nanoparticles exploit both receptor‐dependent uptake and intrinsic macrophage endocytic programs for efficient and targeted intracellular delivery, and the incorporation of DOTAP does not compromise the folate‐mediated targeting ability.

Building on the cationic surface potential imparted by DOTAP incorporation (Figure S19), we hypothesized that FA‐LNP^+^ could enhance autophagic activity in macrophages (Figure 5 h) [41]. Immunofluorescence staining confirmed enhanced LC3 puncta formation in macrophages treated with FA‐LNP^+^ (Figure 5i,j), indicating activation of autophagic processes. Ultrastructural examination by TEM further confirmed the presence of increased double‐membraned autophagosomes in FA‐LNP^+^‐treated cells relative to controls (Figure 5i,k). Western blotting demonstrated significantly elevated conversion to LC3‐II, a canonical autophagosome marker, in macrophages exposed to FA‐LNP^+^ compared to both untreated cells and the DOTAP‐free FA‐LNP formulation (Figure 5l). Consistently, proteomic profiling showed that LC3B protein levels were markedly increased in the FA‐LNP^+^ group compared to the FA‐LNP group (Figure 5m). Moreover, GO enrichment analysis revealed that FA‐LNP^+^ treatment prominently engaged processes related to membrane raft and endosome‐lysosome organization (Figure 5n). Consistently, GSEA further demonstrated significant upregulation of transmembrane transporter activity in FA‐LNP^+^ cells compared to FA‐LNP (Figure 5o), suggesting that the incorporation of DOTAP promotes membrane dynamics and function, thereby facilitating autophagosome formation.

To determine whether enhanced autophagic activity improved STING degradation, we quantified protein levels in LPS‐stimulated macrophages across LNP formulations (Figure 5p). Despite equivalent STING‐ATTEC loading, the FA‐LNP^+^ formulation resulted in more pronounced STING reduction compared to FA‐LNP, suggesting a synergistic effect. This enhancement is likely driven by the autophagy‐promoting properties of cationic FA‐LNP^+^, which facilitates lysosomal trafficking and degradation of STING. These findings support a strategic approach of coupling surface charge modulation with targeted degradation to amplify intracellular efficacy.

To validate the anti‐inflammatory efficacy, LPS‐stimulated macrophages were treated with various LNP formulations. qRT‐PCR analysis first revealed a marked reduction in IL‐1β and TNF‐α mRNA upon FA‐LNP^+^@STING‐ATTEC treatment compared with controls (Figure 5q). In line with these transcriptional changes, immunofluorescence staining of IL‐1β confirmed a pronounced decrease in fluorescence intensity in the FA‐LNP^+^@STING‐ATTEC group relative to other treatment groups (Figure 5r). Consistently, Western blot analysis further demonstrated that FA‐LNP^+^@STING‐ATTEC effectively suppressed the expression of STING, iNOS, and IL‐1β, outperforming both the unloaded FA‐LNP^+^ and the STING‐targeting control (FA‐LNP^+^@C170) (Figure 5s). These results demonstrate that coupling STING degradation with DOTAP‐enhanced autophagy leads to synergistic attenuation of inflammatory signaling. Collectively, our multimodal data establish FA‐LNP^+^@STING‐ATTEC as a potent anti‐inflammatory nanoplatform that targets STING for degradation and effectively dampens pro‐inflammatory cytokine cascades.

### 2.6 In Vivo Validation of FA‐LNP^+^@STING‐ATTEC Reveals Broad Therapeutic Efficacy in Inflammatory Diseases

**FIGURE 6:**
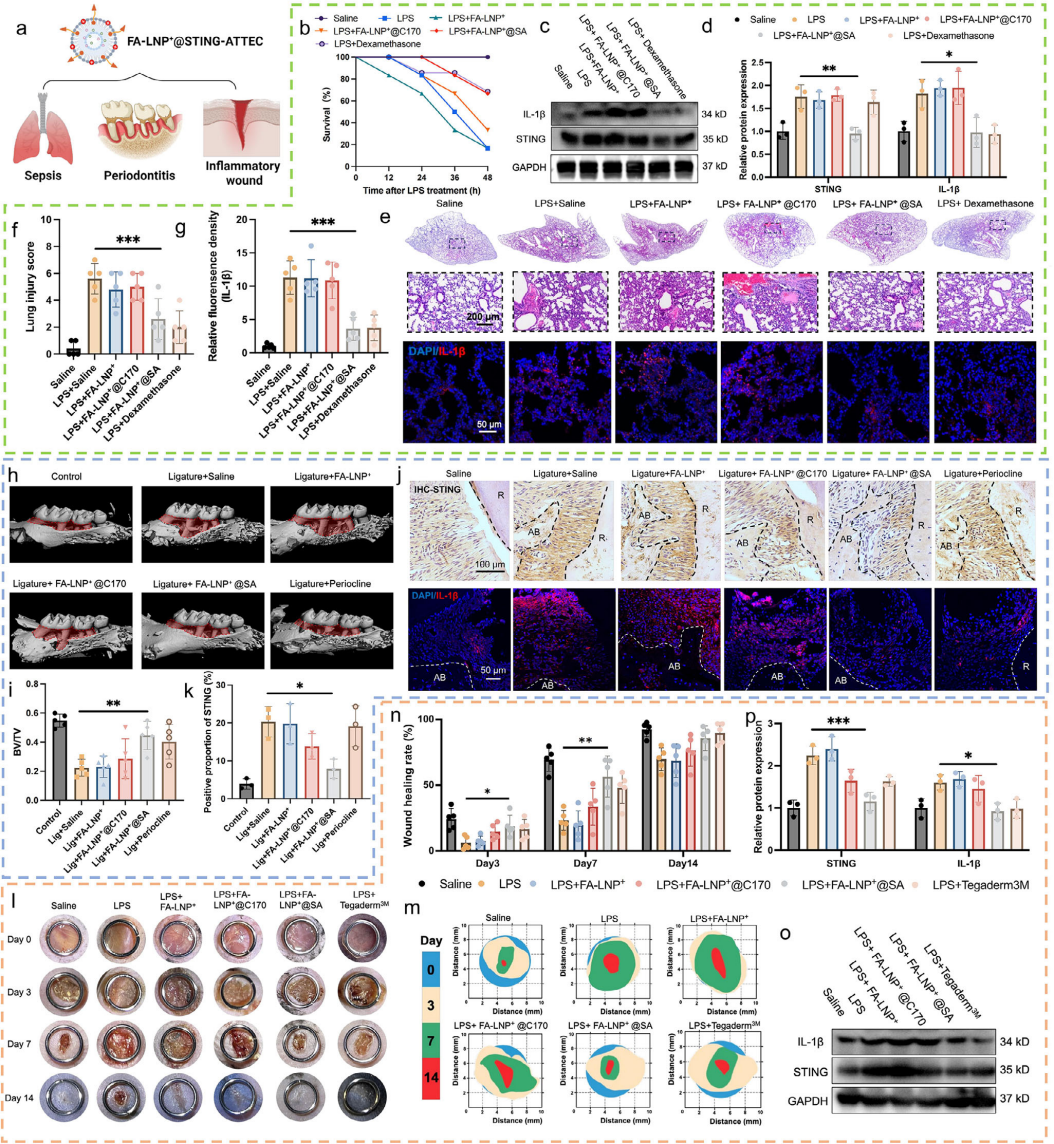
Therapeutic efficacy of FA‐LNP^+^@STING‐ATTEC in inflammatory disease models. (a) Schematic illustration of FA‐LNP^+^@STING‐ATTEC administration in sepsis, periodontitis, and inflammatory wound healing models. (b) Survival curves of mice with LPS‐induced sepsis following treatment. (c,d) Western blot analysis and quantification of STING and IL‐1β protein expression in lung tissues (n = 3, per group). (e) H&E staining and IL‐1β immunofluorescence staining. (f) Quantification of lung injury scores (n = 5, per group). (g) Quantification of IL‐1β immunofluorescence density (n = 5, per group). (h) Micro‐CT images of alveolar bone resorption in periodontitis models. (i) Quantification of bone volume/total volume (BV/TV) ratio (n = 5, per group). (j) Immunohistochemical staining of STING and immunofluorescence staining of IL‐1β in alveolar bone. (k) Quantification of immunohistochemical staining intensity (n = 5, per group). (l) Photographs of the wounds. (m) Schematic diagram illustrating the progress of wound healing over the course of 14 days under different treatment conditions. (n) Quantitative data of the relative wound area at different time points (n = 5, per group). (o,p) Western blot analysis and quantification of STING and IL‐1β in skin tissues (n = 3, per group). Statistical significance: *p < 0.05, **p < 0.01, ***p < 0.001, ****p < 0.0001. (FA‐LNP^+^@SA denotes FA‐LNP^+^@STING‐ATTEC throughout the figure).

To validate the in vivo therapeutic efficacy of FA‐LNP^+^@STING‐ATTEC, we employed multiple inflammatory disease models (Figure 6a). In the sepsis model, FA‐LNP^+^@STING‐ATTEC treatment conferred significant survival benefits comparable to dexamethasone and superior to FA‐LNP^+^ or FA‐LNP^+^@C170 alone (Figure 6b). Western blot analysis of lung tissues revealed that FA‐LNP^+^@STING‐ATTEC uniquely reduced STING protein expression, consistent with its mechanism of targeted degradation, while both FA‐LNP^+^@STING‐ATTEC and dexamethasone effectively suppressed IL‐1β (Figure 6c–d). This distinction highlights the mechanistic precision of FA‐LNP^+^@STING‐ATTEC in disrupting upstream signaling cascades, contrasting with the broad immunosuppressive action of dexamethasone. Histological analysis further revealed attenuated lung injury in both treatment groups, with FA‐LNP^+^@STING‐ATTEC–treated mice showing preserved alveolar architecture and reduced inflammatory infiltration on H&E staining, as well as decreased IL‐1β signal in immunofluorescence (Figure 6e). Quantification of lung injury scores and IL‐1β expression indicated that FA‐LNP^+^@STING‐ATTEC produced similar anti‐inflammatory effects to dexamethasone in the sepsis model (Figure 6f,g). In addition, systemic safety was evaluated by measuring serum biochemical indicators, including ALT, AST, BUN, and creatinine. No significant differences were observed between the FA‐LNP^+^@STING‐ATTEC and dexamethasone‐treated groups, indicating comparable systemic safety profiles under the tested conditions (Figure S20).

We next assessed FA‐LNP^+^@STING‐ATTEC in ligature‐induced periodontitis, a model of chronic inflammatory bone loss. Micro‐CT analysis demonstrated significant alveolar bone preservation with FA‐LNP^+^@STING‐ATTEC, blunting bone loss to levels comparable to Periocline (antibiotic control) (Figure 6h). Histological examination confirmed reduced structural degradation (Figure S21). Quantitative analysis of BV/TV and CEJ–ABC distance showed that FA‐LNP^+^@STING‐ATTEC achieved a level of bone preservation equivalent to that of Periocline, and markedly superior to vehicle or single‐component controls (Figure 6i; Figure S22). Immunohistochemical/immunofluorescence profiling of the local inflammatory microenvironment showed the exclusive suppression of STING expression by FA‐LNP^+^@STING‐ATTEC and equivalent IL‐1β inhibition vs. Periocline (Figure 6j,k).

Finally, we established a standardized full‐thickness cutaneous wound model under inflammatory conditions to evaluate tissue regeneration. Throughout the 14‐day observation period, the FA‐LNP^+^@STING‐ATTEC treatment group demonstrated markedly enhanced wound closure kinetics, achieving comparable efficacy to the clinically approved Tegaderm^3M^ dressing (Figure 6l,m). Quantitative analysis revealed statistically significant differences in wound area reduction at day 7, with the FA‐LNP^+^@STING‐ATTEC group exhibiting superior healing rates relative to all control groups, including the Tegaderm^3M^ benchmark (Figure 6n). Western blot analysis of wound tissues demonstrated that FA‐LNP^+^@STING‐ATTEC specifically downregulated both STING signaling and IL‐1β expression, whereas conventional treatments predominantly modulated downstream inflammatory mediators (Figure 6o,p). Histological staining corroborated these observations. H&E staining demonstrated improved tissue architecture, while Masson's trichrome staining indicated significantly increased collagen deposition in the FA‐LNP^+^@STING‐ATTEC cohort, evidencing robust extracellular matrix remodeling (Figure S23). These findings collectively establish that targeted autophagic degradation of STING via FA‐LNP^+^@STING‐ATTEC not only attenuates inflammatory responses but also promotes functionally competent tissue repair through dual modulation of immune signaling and matrix reorganization.

## 3 Discussion

Our study identifies dysregulated degradation of STING as a conserved driver of pathological inflammation and demonstrates that targeted restoration of this process through a biomimetic nanodegrader offers a rational therapeutic strategy. By systematically interrogating human specimens, murine models, and single‐cell datasets, we confirmed that STING is upregulated across diverse inflammatory contexts and is primarily enriched in macrophages. Functional knockout experiments further established STING as an indispensable regulator of cytokine release, tissue injury, and impaired repair, underscoring its potential as a central therapeutic target. Mechanistically, we revealed that physiological STING degradation depends on ESCRT‐mediated lysosomal trafficking, yet this process is compromised under inflammatory stress, thereby prolonging signaling. This provides a mechanistic explanation for sustained cytokine release in chronic inflammatory states and validates degradation restoration as a rational intervention point.

Targeted degradation of STING represents an alternative strategy to conventional pharmacological inhibition. While small‐molecule inhibitors typically suppress signaling by blocking enzymatic activity or ligand binding, protein degradation eliminates the entire protein from the cellular system, which may provide a more sustained suppression of STING signaling and potentially overcome limitations associated with reversible inhibition [45]. Recently, targeted protein degradation strategies based on ATTECs have emerged as an effective approach for selectively degrading disease‐associated proteins through recruitment to LC3 and the autophagy machinery [46]. Previous studies have demonstrated the feasibility of this strategy in degrading aggregation‐prone or pathogenic proteins [47, 48]. Beyond ATTEC‐mediated autophagic degradation, other targeted protein degradation strategies are also being explored and may provide complementary opportunities for modulating disease‐relevant signaling pathways. For example, the recently reported STING‐directed molecular glue NVS‐STG2 demonstrates that smaller molecules can engage STING and its interacting partners with high specificity [49]. Together, these emerging approaches highlight the potential of targeted degradation as a versatile strategy for modulating STING signaling.

Building on this emerging framework of targeted protein degradation, we designed a triazole‐linked STING‐ATTEC that mimics the ESCRT pathway by redirecting STING toward the autophagy–lysosome axis. This design concept leverages the natural crosstalk between membrane trafficking pathways and autophagic degradation to achieve selective elimination of STING. Deep learning–assisted molecular design and structural docking enabled optimization of ternary complex stability between STING, LC3, and the degrader, ultimately yielding a compound with high specificity and degradation potency. The resulting molecule effectively promotes the formation of a STING–ATTEC–LC3 ternary complex, thereby facilitating the recruitment of STING to the autophagic machinery. Importantly, STING‐ATTEC overcame limitations of classical UPS‐based degraders, achieving lysosomal clearance of a membrane‐associated protein. By harnessing the autophagy–lysosome system rather than the ubiquitin–proteasome pathway, this strategy provides a feasible approach for degrading membrane‐associated or compartmentalized proteins that are typically less accessible to proteasome‐dependent degradation mechanisms.

To further explore the cellular consequences of STING degradation, we performed proteomic profiling, which revealed broad remodeling of the inflammatory proteome following STING‐ATTEC treatment. STING was among the most significantly downregulated proteins, accompanied by marked reductions in key inflammatory mediators such as TNFα and IL‐1β. In addition to these canonical inflammatory factors, several other proteins were also differentially regulated. Notably, heme oxygenase‐1 (HO‐1) was markedly upregulated following STING‐ATTEC treatment. HO‐1 is a well‐established cytoprotective enzyme and a downstream target of the Nrf2 antioxidant pathway, suggesting that attenuation of STING signaling may help restore cellular redox homeostasis under inflammatory stress [50]. In contrast, several inflammation‐associated proteins, including Toll‐like receptor 2 (TLR2) and prostaglandin G/H synthase 2 (PTGS2/COX‐2), were significantly downregulated, consistent with the suppression of pro‐inflammatory signaling. In addition to these STING‐related changes, some proteomic alterations may arise from secondary cellular responses, such as reduced inflammatory stress or modulation of the autophagy–lysosome system. Together, these results support the notion that targeted degradation of STING not only suppresses core inflammatory signaling but also reshapes broader cellular stress‐response pathways. However, given the complexity of cellular proteomic responses, further studies are required to rigorously distinguish STING‐dependent effects from potential off‐target changes. To this end, future studies could perform comparative proteomic analyses in STING^−/−^ cells treated with STING‐ATTEC. Proteins whose changes are abolished in STING^−/−^ cells would likely represent STING‐dependent effects, whereas those that persist may indicate off‐target responses. In addition, rescue experiments with STING re‐expression could further validate the specificity of STING‐dependent proteomic changes.

In order to translate this degradation strategy into an effective therapeutic platform, we further integrated STING‐ATTEC with a folate‐modified cationic lipid nanoparticle delivery system (FA‐LNP^+^). This formulation provides two key advantages: selective macrophage uptake via folate receptor–mediated endocytosis and intrinsic autophagy stimulation associated with DOTAP‐containing cationic lipids. Through this dual mechanism, FA‐LNP^+^ not only enhances intracellular delivery of STING‐ATTEC but also promotes LC3 generation, thereby synergistically amplifying STING degradation. Consistent with this mechanism, FA‐LNP^+^@STING‐ATTEC effectively suppressed inflammatory cascades and alleviated tissue injury across multiple inflammatory models, including sepsis, periodontitis, and wound healing. Importantly, this strategy represents a mechanistically distinct therapeutic approach compared with conventional anti‐inflammatory drugs. Unlike dexamethasone, which exerts broad immunosuppressive effects, STING‐ATTEC selectively induces autophagy‐mediated degradation of STING, while folate‐modified nanoparticles enhance macrophage‐targeted delivery. In addition, compared with clinically used local treatments such as Periocline or Tegaderm, which mainly provide antimicrobial or protective effects, FA‐LNP^+^@STING‐ATTEC directly modulates the underlying inflammatory signaling pathway. Although the overall anti‐inflammatory efficacy was comparable to these treatments in our models, the mechanism of action is fundamentally distinct, highlighting a targeted degradation strategy for regulating STING‐driven inflammation.

Taken together, these findings establish targeted autophagic degradation of STING as a versatile therapeutic strategy for inflammation‐driven diseases. Conceptually, our work highlights a generalizable framework in which degraders inspired by endogenous trafficking mechanisms can be rationally engineered, computationally optimized, and nanotechnologically delivered to overcome pharmacological barriers. While this study focused on STING, the strategy may be broadly extensible to other membrane‐bound or vesicular proteins that are traditionally less accessible to UPS‐dependent degraders.

Despite these promising findings, several limitations should be acknowledged. The current STING‐ATTEC molecule is a heterobifunctional degrader with a relatively high molecular weight, a characteristic common to chimeric degraders that may restrict oral bioavailability and membrane permeability. Future optimization will therefore be required to improve physicochemical properties while preserving degradation efficiency. In addition, further studies are needed to evaluate the long‐term safety, immunogenicity, and pharmacokinetics of FA‐LNP^+^@STING‐ATTEC in clinically relevant models. Addressing these questions will also help clarify the broader applicability of ATTEC‐based degraders, the safety of sustained autophagy modulation, and opportunities for combinatorial use with existing anti‐inflammatory or regenerative therapies.

Together, this work expands the translational landscape of targeted protein degradation and underscores lysosome‐directed degraders as a promising therapeutic paradigm for inflammatory disorders.

## 4 Methods

Detailed methods are provided in the Supplementary Information.

## Conflicts of Interest

The authors declare the following competing interests: T.C., L.X., F.Z., J.S., Q.Z. and S.W. have submitted a patent application (202511247049.1) related to this study. The other authors declare no competing interests.

## Supporting information




**Supporting File**: advs74919‐sup‐0001‐SuppMat.docx.

## Data Availability

The data that support the findings of this study are available in the Supplementary Material of this article.

## References

[1] P. T. Pham , D. Fukuda , S. Nishimoto , et al., “STING, a Cytosolic DNA Sensor, Plays a Critical Role in Atherogenesis: A Link Between Innate Immunity and Chronic Inflammation Caused by Lifestyle‐Related Diseases,” European Heart Journal 42, no. 42 (2021): 4336–4348, 10.1093/eurheartj/ehab249. 34226923

[2] I. K. Vila , H. Chamma , A. Steer , et al., “STING Orchestrates the Crosstalk Between Polyunsaturated Fatty Acid Metabolism and Inflammatory Responses,” Cell Metabolism 34, no. 1 (2022): 125–139.e8, 10.1016/j.cmet.2021.12.007. 34986331 PMC8733004

[3] K. R. Balka , R. Venkatraman , T. L. Saunders , et al., “Termination of STING Responses is Mediated via ESCRT ‐Dependent Degradation,” The EMBO Journal 42, no. 12 (2023): 112712, 10.15252/embj.2022112712. PMC10267698 37139896

[4] Y. Cao , X. Chen , Z. Zhu , et al., “STING Contributes to Lipopolysaccharide‐Induced Tubular Cell Inflammation and Pyroptosis by Activating Endoplasmic Reticulum Stress in Acute Kidney Injury,” Cell Death & Disease 15, no. 3 (2024): 217, 10.1038/s41419-024-06600-1. 38485717 PMC10940292

[5] Y. Guo , Y. You , F.‐F. Shang , et al., “iNOS Aggravates Pressure Overload‐Induced Cardiac Dysfunction via Activation of the Cytosolic‐mtDNA‐Mediated cGAS‐STING Pathway,” Theranostics 13, no. 12 (2023): 4229–4246, 10.7150/thno.84049. 37554263 PMC10405855

[6] B. Zhang , A. Pedersen , L. S. Reinert , et al., “STING Signals to NF‐κB From Late Endolysosomal Compartments Using IRF3 as an Adaptor,” Nature Immunology 26 (2025): 1916–1930, 10.1038/s41590-025-02283-8.40973797

[7] S. Zhang , R. Zheng , Y. Pan , and H. Sun , “Potential Therapeutic Value of the STING Inhibitors,” Molecules (Basel, Switzerland) 28, no. 7 (2023): 3127, 10.3390/molecules28073127. 37049889 PMC10096477

[8] X. Sun , L. Liu , J. Wang , et al., “Targeting STING in Dendritic Cells Alleviates Psoriatic Inflammation by Suppressing IL‐17A Production,” Cellular & Molecular Immunology 21, no. 7 (2024): 738–751, 10.1038/s41423-024-01160-y. 38806624 PMC11214627

[9] F. Chang , C. Gunderstofte , N. Colussi , et al., “Development of Nitroalkene‐Based Inhibitors to Target STING‐Dependent Inflammation,” Redox Biology 74 (2024): 103202, 10.1016/j.redox.2024.103202. 38865901 PMC11215336

[10] F. Guo , J. Zhang , Y. Gao , et al., “Discovery and Total Synthesis of Anhydrotuberosin as a STING Antagonist for Treating Autoimmune Diseases,” Angewandte Chemie International Edition 64, no. 1 (2025): 202407641, 10.1002/anie.202407641. 39471366

[11] X. Li , Z. Yu , Q. Fang , et al., “The Transmembrane Endoplasmic Reticulum–Associated E3 Ubiquitin Ligase TRIM13 Restrains the Pathogenic‐DNA–Triggered Inflammatory Response,” Science Advances 8, no. 4 (2022): abh0496, 10.1126/sciadv.abh0496. PMC8791621 35080984

[12] V. K. Gonugunta , T. Sakai , V. Pokatayev , et al., “Trafficking‐Mediated STING Degradation Requires Sorting to Acidified Endolysosomes and Can Be Targeted to Enhance Anti‐Tumor Response,” Cell Reports 21, no. 11 (2017): 3234–3242, 10.1016/j.celrep.2017.11.061. 29241549 PMC5905341

[13] M. Gentili , B. Liu , M. Papanastasiou , et al., “ESCRT‐Dependent STING Degradation Inhibits Steady‐State and cGAMP‐Induced Signalling,” Nature Communications 14, no. 1 (2023): 611, 10.1038/s41467-023-36132-9. PMC9899276 36739287

[14] Y. Zhao , C. Zhou , W. Tian , et al., “A Specific Module of ESCRT Regulates STING Activity Termination by Controlling STING Degradation,” Science Bulletin 69, no. 8 (2024): 1000–1005, 10.1016/j.scib.2024.01.005. 38272732

[15] Y. Kuchitsu , K. Mukai , R. Uematsu , et al., “STING Signalling Is Terminated Through ESCRT‐Dependent Microautophagy of Vesicles Originating From Recycling Endosomes,” Nature Cell Biology 25, no. 3 (2023): 453–466, 10.1038/s41556-023-01098-9. 36918692 PMC10014584

[16] M. Gentili , B. Liu , M. Papanastasiou , et al., “ESCRT‐Dependent STING Degradation Inhibits Steady‐State and cGAMP‐Induced Signalling,” Nature Communications 14, no. 1 (2023): 611, 10.1038/s41467-023-36132-9. PMC9899276 36739287

[17] X. Li , X. Chen , L. Zheng , et al., “Non‐Canonical STING–PERK Pathway Dependent Epigenetic Regulation of Vascular Endothelial Dysfunction via Integrating IRF3 and NF‐κB in Inflammatory Response,” Acta Pharmaceutica Sinica B 13, no. 12 (2023): 4765–4784, 10.1016/j.apsb.2023.08.015. 38045042 PMC10692388

[18] H. Mao , L. Zhou , J. Li , Y. Wen , Z. Chen , and L. Zhang , “STING Inhibition Alleviates Bone Resorption in Apical Periodontitis,” International Endodontic Journal 57 (2024): 951–965, 10.1111/iej.14057. 38411951

[19] S. Skopelja‐Gardner , J. An , and K. B. Elkon , “Role of the cGAS–STING Pathway in Systemic and Organ‐Specific Diseases,” Nature Reviews Nephrology 18, no. 9 (2022): 558–572, 10.1038/s41581-022-00589-6. 35732833 PMC9214686

[20] J. Liu , L. Yuan , Y. Ruan , et al., “Novel CRBN‐Recruiting Proteolysis‐Targeting Chimeras as Degraders of Stimulator of Interferon Genes with in Vivo Anti‐Inflammatory Efficacy,” Journal of Medicinal Chemistry 65, no. 9 (2022): 6593–6611, 10.1021/acs.jmedchem.1c01948. 35452223

[21] X. Wang , Y. Zhao , X. Li , et al., “Liposomal STAT3‐Degrading PROTAC Prodrugs Promote Anti‐Hepatocellular Carcinoma Immunity via Chemically Reprogramming Cancer Stem Cells,” Nano Letters 24 (2024): 4858–4868, 10.1021/acs.nanolett.4c00201. 38598369

[22] X. Cheng , S. Hu , and K. Cheng , “Microneedle Patch Delivery of PROTACs for Anti‐Cancer Therapy,” ACS Nano 17, no. 12 (2023): 11855–11868, 10.1021/acsnano.3c03166. 37294705 PMC11393661

[23] J. Xu , X. Zhao , X. Liang , et al., “Development of miRNA‐Based PROTACs Targeting Lin28 for Breast Cancer Therapy,” Science Advances 10, no. 38 (2024): adp0334, 10.1126/sciadv.adp0334. PMC11409961 39292784

[24] F. Yang , Q. Luo , Y. Wang , et al., “Targeted Biomolecule Regulation Platform: A Split‐and‐Mix PROTAC Approach,” Journal of the American Chemical Society 145, no. 14 (2023): 7879–7887, 10.1021/jacs.2c12824. 37001133

[25] Y. Chen , S. Pal , W. Li , F. Liu , S. Yuan , and Q. Hu , “Engineered Platelets as Targeted Protein Degraders and Application to Breast Cancer Models,” Nature Biotechnology 43 (2024): 1800–1812, 10.1038/s41587-024-02494-8. PMC12130346 39627511

[26] Z. Li , C. Wang , Z. Wang , et al., “Allele‐Selective Lowering of Mutant HTT Protein by HTT–LC3 Linker Compounds,” Nature 575, no. 7781 (2019): 203–209, 10.1038/s41586-019-1722-1. 31666698

[27] X. Li , Q. Liu , X. Xie , et al., “Application of Novel Degraders Employing Autophagy for Expediting Medicinal Research,” Journal of Medicinal Chemistry 66, no. 3 (2023): 1700–1711, 10.1021/acs.jmedchem.2c01712. 36716420

[28] S. Tan , D. Wang , Y. Fu , H. Zheng , Y. Liu , and B. Lu , “Targeted Clearance of Mitochondria by an Autophagy‐Tethering Compound (ATTEC) and Its Potential Therapeutic Effects,” Science Bulletin 68, no. 23 (2023): 3013–3026, 10.1016/j.scib.2023.10.021. 37940449

[29] K. Yin , Z. Zhang , Y. Mo , et al., “Discovery of Autophagy‐Tethering Compounds as Potent NLRP3 Degraders for IBD Immunotherapy,” European Journal of Medicinal Chemistry 275 (2024): 116581, 10.1016/j.ejmech.2024.116581. 38870831

[30] Y. Fu and B. Lu , “Targeting Lipid Droplets for Autophagic Degradation by ATTEC,” Autophagy 17, no. 12 (2021): 4486–4488, 10.1080/15548627.2021.1967616. 34436975 PMC8726627

[31] Y. Zhang , J. Huang , Y. Liang , et al., “Clearance of Lipid Droplets by Chimeric Autophagy‐Tethering Compound Ameliorates the Age‐Related Macular Degeneration Phenotype in Mice Lacking APOE,” Autophagy 19, no. 10 (2023): 2668–2681, 10.1080/15548627.2023.2220540. 37266932 PMC10472852

[32] T. Roh , S. Ju , S. Y. Park , et al., “Fucosylated Haptoglobin Promotes Inflammation via Mincle in Sepsis: An Observational Study,” Nature Communications 16, no. 1 (2025): 1342, 10.1038/s41467-025-56524-3. PMC11794430 39904983

[33] J. Oh , Y. Kim , H. Son , Y. H. Kim , and H. Kim , “Comparative Transcriptome Analysis of Periodontitis and Peri‐Implantitis in Human Subjects,” Journal of Periodontology 95, no. 4 (2024): 337–349, 10.1002/JPER.23-0289. 37789641

[34] H. Johnsson , J. Cole , S. Siebert , I. B. McInnes , and G. Graham , “Cutaneous Lesions in Psoriatic Arthritis Are Enriched in Chemokine Transcriptomic Pathways,” Arthritis Research & Therapy 25, no. 1 (2023): 73, 10.1186/s13075-023-03034-6. 37131254 PMC10152590

[35] K. Zhang , Y. Wang , S. Chen , et al., “TREM2^hi^ Resident Macrophages Protect the Septic Heart by Maintaining Cardiomyocyte Homeostasis,” Nature Metabolism 5, no. 1 (2023): 129–146, 10.1038/s42255-022-00715-5. PMC9886554 36635449

[36] Y. Fu , N. Chen , Z. Wang , S. Luo , Y. Ding , and B. Lu , “Degradation of Lipid Droplets by Chimeric Autophagy‐Tethering Compounds,” Cell Research 31, no. 9 (2021): 965–979, 10.1038/s41422-021-00532-7. 34239073 PMC8410765

[37] R. Zang , L. Xue , M. Zhang , et al., “Design and Syntheses of a Bimolecular STING Agonist Based on the Covalent STING Antagonist,” European Journal of Medicinal Chemistry 250 (2023): 115184, 10.1016/j.ejmech.2023.115184. 36758305

[38] F. Li , Q. Hu , X. Zhang , et al., “DeepPROTACs Is a Deep Learning‐Based Targeted Degradation Predictor for PROTACs,” Nature Communications 13, no. 1 (2022): 7133, 10.1038/s41467-022-34807-3. PMC9681730 36414666

[39] W. Zhao , Y. Jiang , X. Li , and H. Wang , “Nanotechnology‐Enabled Targeted Protein Degradation for Cancer Therapeutics,” WIREs Nanomedicine and Nanobiotechnology 16, no. 6 (2024): 2020, 10.1002/wnan.2020. 39663650

[40] Y. Yu , L. Kong , R. Guo , et al., “Engineered Panax Notoginseng Polysaccharide Micelles Inhibit Macrophage Polarization and Delay the Progression of Rheumatoid Arthritis via JAK2‐STAT3 Signaling Pathway,” Journal of Nanobiotechnology 23, no. 1 (2025): 509, 10.1186/s12951-025-03576-8. 40660236 PMC12261558

[41] X. Huang , Z. Cao , J. Qian , et al., “Nanoreceptors Promote Mutant P53 Protein Degradation by Mimicking Selective Autophagy Receptors,” Nature Nanotechnology 19, no. 4 (2024): 545–553, 10.1038/s41565-023-01562-5. 38216684

[42] Y. Liu , R. Liu , J. Dong , et al., “Targeted Protein Degradation via Cellular Trafficking of Nanoparticles,” Nature Nanotechnology 20 (2024): 296–302, 10.1038/s41565-024-01801-3. 39468359

[43] J. Gilleron , W. Querbes , A. Zeigerer , et al., “Image‐Based Analysis of Lipid Nanoparticle–Mediated siRNA Delivery, Intracellular Trafficking and Endosomal Escape,” Nature Biotechnology 31, no. 7 (2013): 638–646, 10.1038/nbt.2612. 23792630

[44] W. He , C. Chen , J. Zheng , et al., “Targeted Degradation of Cell Surface Proteins Through Endocytosis Triggered by Cell‐Penetrating Peptide‐Small Molecule Conjugates,” Nature Communications 16, no. 1 (2025): 7575, 10.1038/s41467-025-62776-w. PMC12354712 40813571

[45] L. Xue , R. Liu , L. Zhang , et al., “Discovery of Novel Nitrofuran PROTAC‐Like Compounds as Dual Inhibitors and Degraders Targeting STING,” European Journal of Medicinal Chemistry 279 (2024): 116883, 10.1016/j.ejmech.2024.116883. 39303513

[46] Z. Li , C. Wang , Z. Wang , et al., “Allele‐Selective Lowering of Mutant HTT Protein by HTT–LC3 Linker Compounds,” Nature 575, no. 7781 (2019): 203–209, 10.1038/s41586-019-1722-1. 31666698

[47] S. Tan , D. Wang , Y. Fu , H. Zheng , Y. Liu , and B. Lu , “Targeted Clearance of Mitochondria by an Autophagy‐Tethering Compound (ATTEC) and its Potential Therapeutic Effects,” Science Bulletin 68, no. 23 (2023): 3013–3026, 10.1016/j.scib.2023.10.021. 37940449

[48] Y. Fu , N. Chen , Z. Wang , S. Luo , Y. Ding , and B. Lu , “Degradation of Lipid Droplets by Chimeric Autophagy‐Tethering Compounds,” Cell Research 31, no. 9 (2021): 965–979, 10.1038/s41422-021-00532-7. 34239073 PMC8410765

[49] J. Li , S. M. Canham , H. Wu , et al., “Activation of Human STING by a Molecular Glue‐Like Compound,” Nature Chemical Biology 20, no. 3 (2024): 365–372, 10.1038/s41589-023-01434-y. 37828400 PMC10907298

[50] L. Xin , F. Zhou , C. Zhang , et al., “Four‐Octyl Itaconate Ameliorates Periodontal Destruction via Nrf2‐Dependent Antioxidant System,” International Journal of Oral Science 14, no. 1 (2022): 27, 10.1038/s41368-022-00177-1. 35637195 PMC9151820

