# Supplementary material for: ESCRT‐Mimetic Nanodegrader Targets STING for Anti‐Inflammatory Therapy
Source: Adv Sci (Weinh). 2026 Mar 24;13(36):e23601. doi: 10.1002/advs.202523601 (PMC13317561; doi:10.1002/advs.202523601)
Supplement: Supplementary file 1 — Supporting File: advs74919‐sup‐0001‐SuppMat.docx. [file ADVS-13-e23601-s001.docx]

**Supplementary Information**

**ESCRT-mimetic Nanodegrader Targets STING for Anti-inflammatory Therapy**

Fuyuan Zhou^1,#^, Qiming Zhai^1,#^, Zhihao Yao^1^, Qing Li^2^, Yan Yang^2^, Si Wang^1^, Chao Huang****^2^, Liangjing Xin***^1^, Tao Chen**^1^, Jinlin Song*^1^

^1^The Affiliated Stomatological Hospital of Chongqing Medical University, Chongqing Key Laboratory of Oral Diseases, Chongqing Municipal Key laboratory of Oral Biomedical Engineering of Higher Education, Chongqing Municipal Health Commission Key Laboratory of Oral Biomedical Engineering, Chongqing Technology Innovation Center of Smart Dental Medical Devices, Innovation and Transformation of Dental Medical Devices Engineering Research Center of Chongqing Education Commission of China, Chongqing, China.

^2^Chongqing Key Laboratory of Natural Product Synthesis and Drug Research, School of Pharmaceutical Sciences, Chongqing University, Chongqing, China.

^#^These authors contributed equally; *Corresponding authors

**Table of Contents Supplementary**

Supplementary Synthesis and Characterization of STING-ATTEC Variants ....................3

Supplementary Methods ....................................................................................................11

Supplementary Figures.......................................................................................................20

Supplementary Reference ..................................................................................................26

**Supplementary Synthesis and Characterization of STING-ATTEC Variants**

**General methods**

All reactions were carried out under a nitrogen atmosphere unless otherwise specified. Solvents were dried and purified according to standard procedures. Reaction progress was monitored by thin-layer chromatography (TLC). Final products were purified by column chromatography on silica gel. Structural confirmation was performed by ^1^H NMR, ^13^C NMR, and high-resolution mass spectrometry (HRMS).

**Synthesis of compound I-1.**

Compound 13 (216 mg, 0.3 mmol) was dissolved in 8 mL dichloromethane (DCM), followed by addition of trifluoroacetic acid (TFA, 2 mL). The mixture was stirred at room temperature for 1 h to remove the Boc protecting group, and the solvent was evaporated. The residue was dissolved in 10 mL DMF and activated with HATU (215 mg, 1.3 mmol, 2.0 equiv.) and DIPEA (109 mg, 0.8 mmol, 3.0 equiv.) for 30 min. A DMF solution of compound 5 (70 mg, 0.3 mmol) was added, and the mixture was stirred for 12 h at room temperature. After aqueous workup and extraction with ethyl acetate, the organic phase was washed with brine, dried over Na₂SO₄, and concentrated. Purification by column chromatography (PE/EA) afforded compound I-1 as a yellow solid.

**Synthesis of compound II-1.** Compound 16 (489 mg, 0.7 mmol) was dissolved in 8 mL DCM, and TFA (2 mL) was added. The reaction proceeded at room temperature for 1 h, followed by solvent evaporation. The residue was dissolved in 10 mL DMF, and HATU (510 mg, 1.3 mmol, 2.0 equiv.) and DIPEA (260 mg, 2.0 mmol, 3.0 equiv.) were introduced. After 30 min activation, a DMF solution of compound 5 (183 mg, 0.74 mmol) was added. The mixture was stirred at room temperature for 12 h. After aqueous workup and column purification, compound II-1 was isolated as a yellow solid.

**Synthesis of compound III-1.** Compound 5 (66 mg, 0.2 mmol) was dissolved in DMF (10 mL) under N₂ atmosphere. Aqueous solutions of CuSO₄ (8 mg, 0.1 mmol) and sodium ascorbate (261 mg, 1.32 mmol) were sequentially added, and the mixture was stirred for 1 h. Compound 19 (133 mg, 0.2 mmol) in DMF (5 mL) was then introduced, and the reaction was maintained at room temperature for 12 h. The crude product was extracted, dried, and purified by column chromatography (DCM/MeOH = 50:1) to yield compound III-1 as a yellow solid.

**^1^H NMR (400 MHz, CDCl_3_)** δ 8.92 (d, *J* = 15.0 Hz, 1H), 8.53 (d, *J* = 11.0 Hz, 2H), 7.71 (d, *J* = 9.7 Hz, 1H), 7.63 (d, *J* = 13.9 Hz, 4H), 7.45 – 7.30 (m, 3H), 6.85 (dd, *J* = 12.5, 8.3 Hz, 1H), 4.11 (d, *J* = 9.1 Hz, 2H), 3.93 (dd, *J* = 12.0, 6.1 Hz, 2H), 3.69 (h, *J* = 9.6, 7.8 Hz, 8H), 3.63 – 3.54 (m, 6H).

**^13^C NMR (600MHz, d-DMSO)** δ 67.85, 69.53, 69.57, 69.71, 69.75, 69.82, 69.84, 70.16, 70.36, 110.08, 113.01, 113.53, 114.44, 114.45, 116.39, 120.02, 121.11, 127.47, 128.05, 133.44, 134.46, 134.94, 137.69, 138.40, 138.72, 148.08, 151.76, 154.38, 165.48, 168.17.

^1^H NMR of compound **I-1** (400 MHz, CDCl_3_)

^13^C NMR of compound **I-1** (600MHz, d-DMSO)


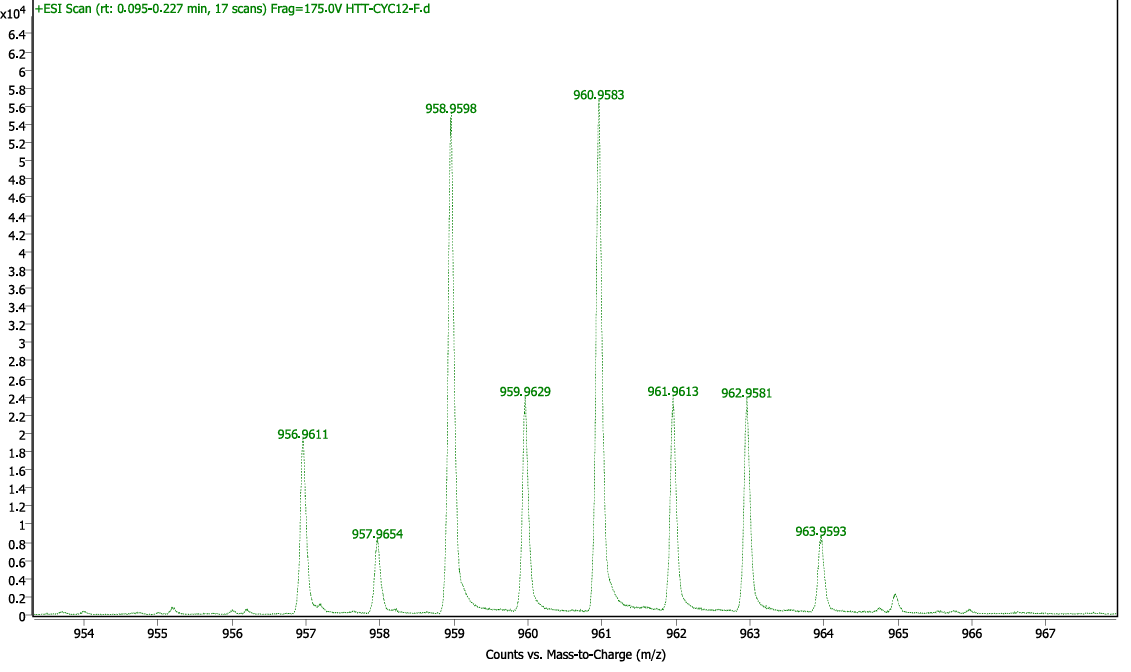


HRMS spectrum of compound **I-1**

**^1^H NMR (400 MHz, d-DMSO)** δ 10.55 (s, 1H), 9.86 (s, 1H), 8.56 (d, *J* = 202.1 Hz, 2H), 7.97 – 7.90 (m, 1H), 7.87 (s, 1H), 7.80 (d, *J* = 3.9 Hz, 1H), 7.61 (q, *J* = 8.9 Hz, 5H), 7.43 (dd, *J* = 8.3, 1.9 Hz, 1H), 7.00 (d, *J* = 8.4 Hz, 1H), 3.71 (t, *J* = 7.2 Hz, 2H), 2.27 (s, 1H), 1.62 – 1.52 (m, 4H), 1.24 (m, *J* = 17.6 Hz, 14H).

**^13^C NMR (400 MHz, d-DMSO)** δ 25.24, 26.36, 27.06, 28.75, 28.85, 28.99, 36.47, 110.59, 111.23, 113.59, 113.81, 116.40, 119.47, 121.28, 122.31, 132.91, 136.12, 136.35, 136.66, 148.17, 151.81, 154.46, 165.10, 171.38.


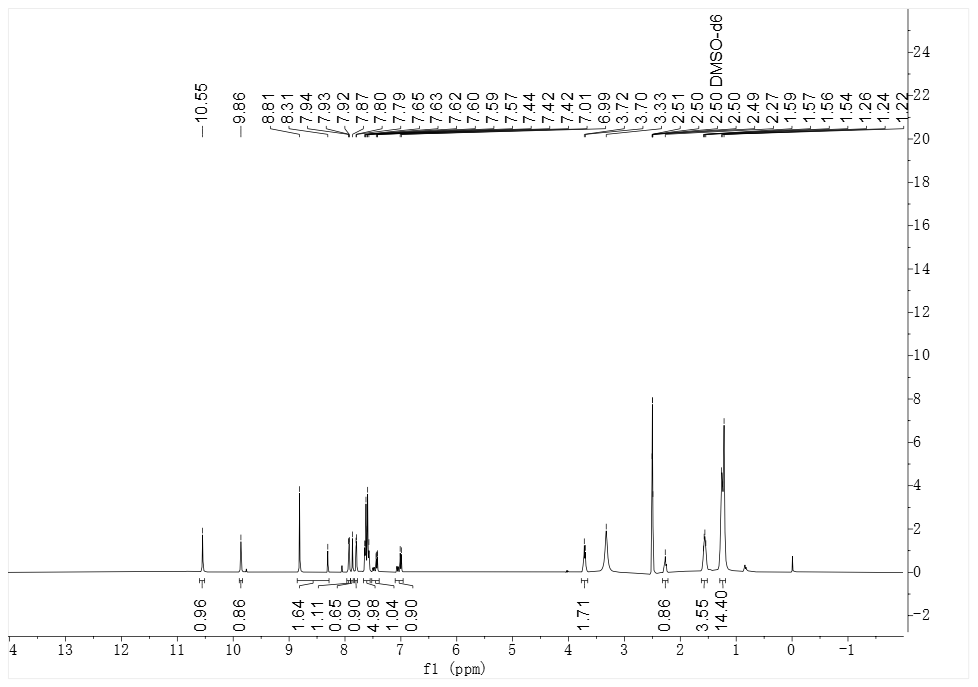


^1^H NMR of compound **II-1** (400 MHz, d-DMSO)


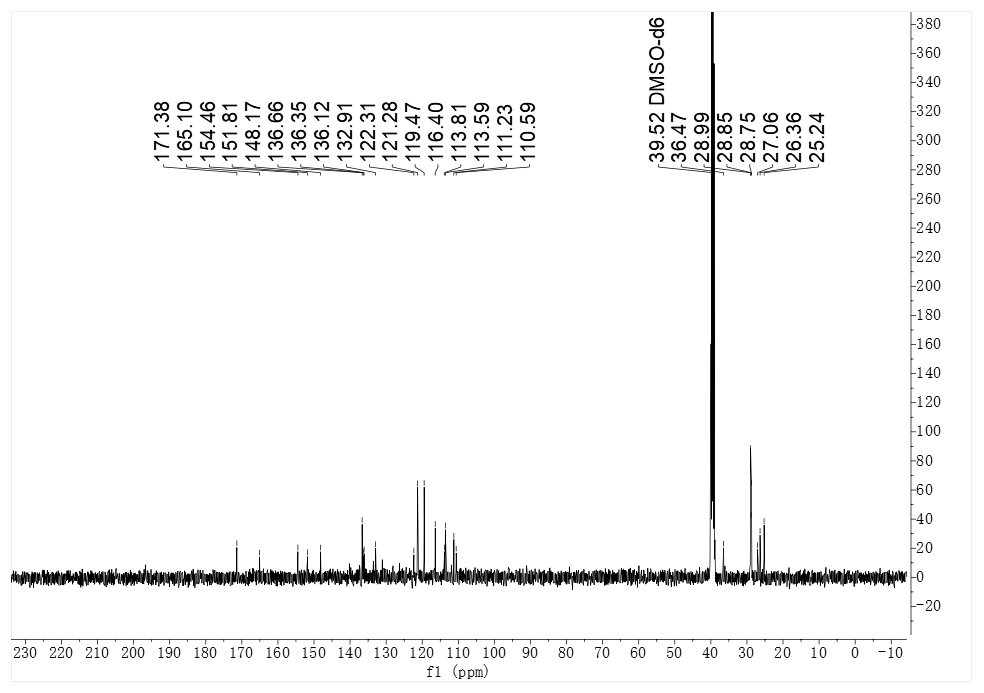


^13^C NMR of compound **II-1** (600MHz, d-DMSO)


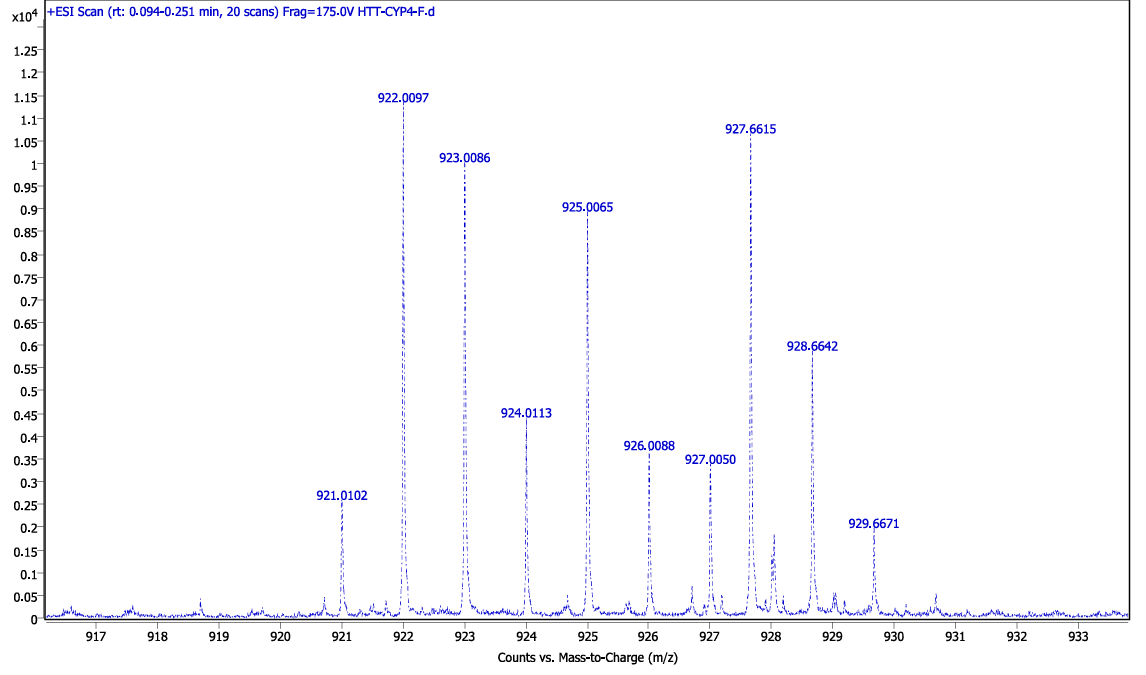


HRMS spectrum of compound **II-1**

**^1^H NMR** (400 MHz, DMSO-*d_6_*) *δ* 8.81 (s, 1H), 7.93 (d, *J* = 8.2 Hz, 1H), 7.88 – 7.76 (m, 2H), 7.67 – 7.54 (m, 6H), 7.46 (dd, *J* = 24.9, 8.3 Hz, 1H), 7.01 (dd, *J* = 27.2, 8.4 Hz, 1H), 4.27 (t, *J* = 6.9 Hz, 2H), 3.68 (q, *J* = 6.5 Hz, 2H), 3.17 (s, 2H), 2.92 (t, *J* = 7.4 Hz, 2H), 2.66 (t, *J* = 7.5 Hz, 2H), 1.74 (t, *J* = 7.3 Hz, 2H), 1.52 (s, 2H), 1.23 (s, 6H).

**^13^C NMR** (101 MHz, DMSO-*d_6_*) *δ* 170.14, 166.60, 165.01, 154.32, 151.72, 148.13, 145.79, 139.90, 136.66, 135.91, 133.53, 132.94, 130.66, 127.47, 126.36, 124.37, 122.37, 121.89, 121.16, 119.38, 116.30, 113.50, 111.94, 111.40, 110.32, 49.14, 35.84, 31.34, 29.65, 29.07, 26.91, 25.74, 25.53, 21.12.


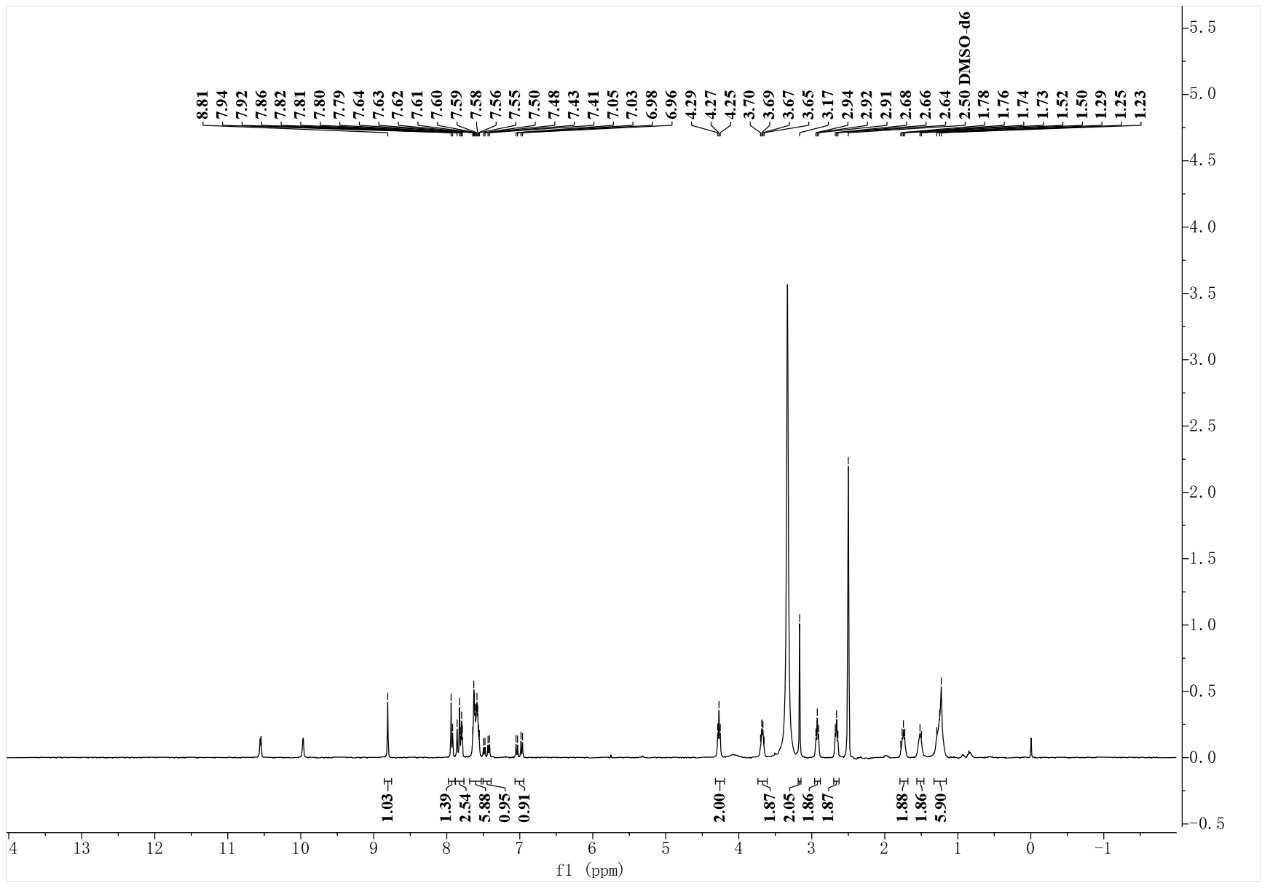


^1^H NMR of compound **III-1** (400 MHz, DMSO-*d_6_*)


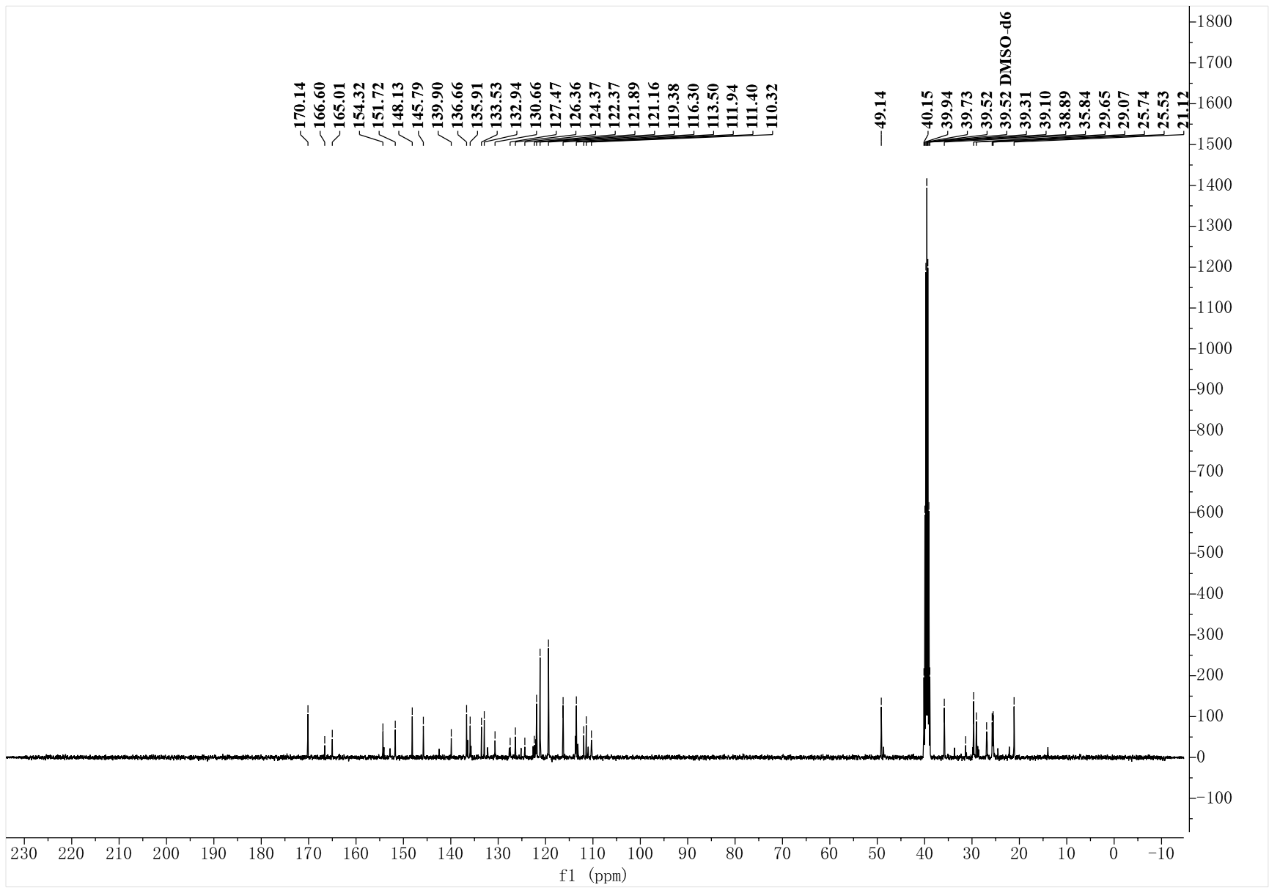


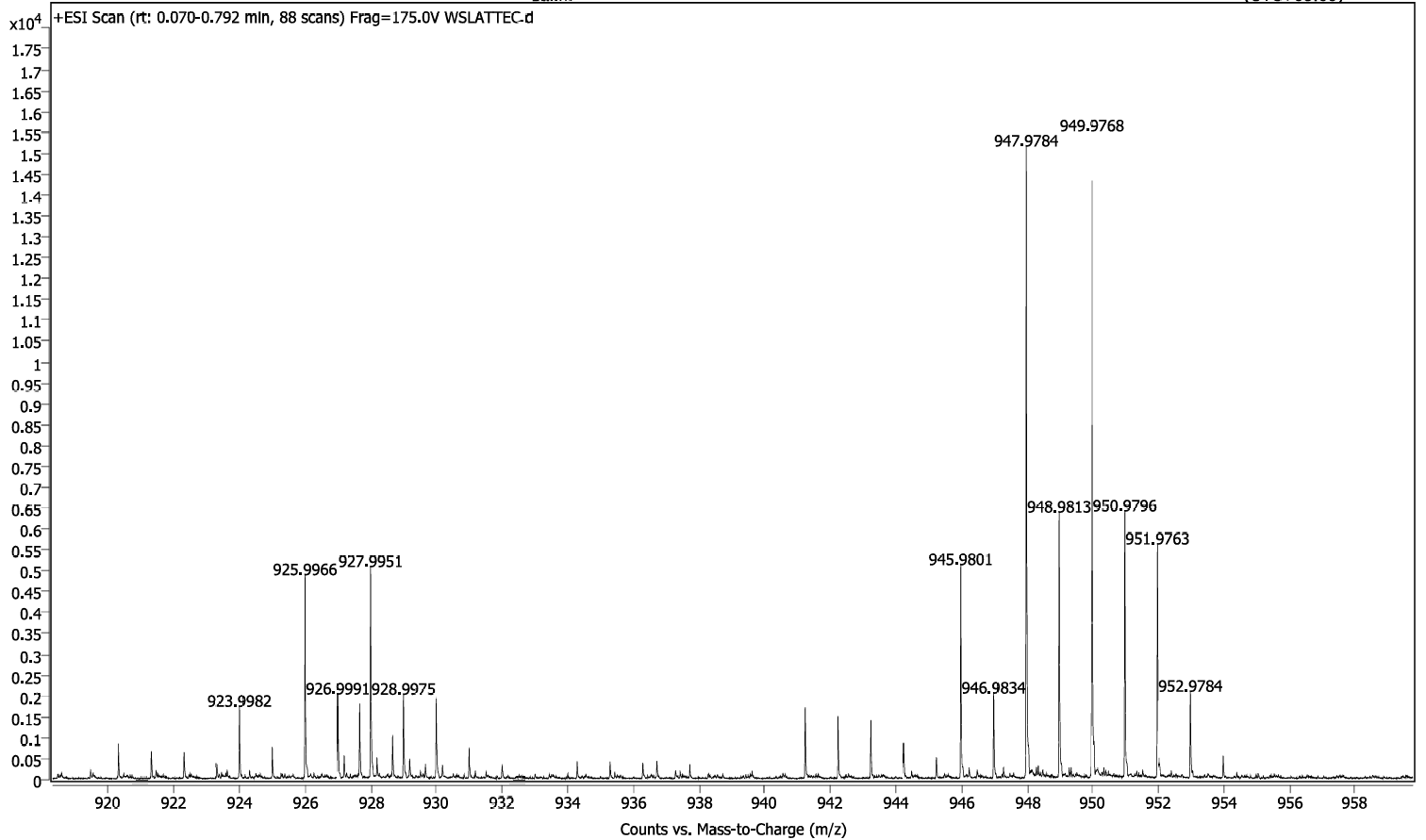
^13^C NMR of compound **III-1** (600MHz, DMSO-*d_6_*)

HRMS spectrum of compound **III-1**

**Supplementary Methods**

**Chemicals and Reagents**

p-phenylenediamine (P108424), potassium fluoride (P164507) and sodium ascorbate (S105024) were purchased from Aladdin. 5-nitrofuran-2-carboxylic acid (BD2571), 4-pentynoic acid (BD2766), 1,2,3,4,6-penta-O-acetyl-α-D-mannopyranose (BD52569), hydrazine acetate (BD134078) and dihydrofuran-2,5-dione (BD152069) were obtained from Bidepharm. Trichloroacetonitrile (M19316), 1,8-diazabicyclo [5.4.0] undec-7-ene (M33021), 2-bromoethanol (M19085), boron trifluoride diethyl etherate (M71534), trimethylsilyl azide (M17163) and cupric sulfate (M33153) were form Meryer. Cytochalasin D (HY-N6682), chlorpromazine (HY-12708), indomethacin (HY-14397), colchicine (HY-16569), wortmannin (HY-10197), methyl-β-cyclodextrin (MβCD, HY-101461), chloroquine (HY-17589A), C170 (HY-138682) and MG-132 (HY-13259) were obtained from MedChemExpress. D-Lin-MC3-DMA (D883836) and 1,2-distearoyl-sn-glycero-3-phosphoethanolamine-N- [folate (polyethylene glycol)-2000] (DSPE-PEG-Folate, D769502) were purchased from Macklin Biochemical Co., Ltd. 1,2-distearoyl-sn-glycero-3-phosphocholine (DSPC, F793317), 1,2-dioleoyl-3-trimethylammonium-propane (DOTAP, D922823) and cholesterol (156754) were from J&K Scientific. DSPE-PEG2000 (BD01530210) was obtained from Bide Pharmatech Ltd. Cy5-cholesterol was purchased from Guangzhou Weihua Biotechnology Co., Ltd. LysoTracker Red DND-99 (L7528) and Lipofectamine RNAiMAX (13778030) were from Invitrogen. Phalloidin (C2201S and C2205S) and DAPI (P0131) were purchased from Beyotime Biotechnology.

**Antibodies**

Antibodies for STING (50494S for western blot, 1:1000, 90947T for immunofluorescence, 1:200), TBK1 (3504S, 1:1000), Phospho-TBK1 (Ser172, 5483T, 1:1000), LC3 (83506T, 1:1000), β-tublin (2128T, 1:1000), GAPDH (2118T, 1:1000) were obtained from Cell Signaling Technology. Antibodies for NF-kB (ab32536, 1:1000), NF-kB (phospho S536, ab76302, 1:1000), IL-1β (ab283818, 1:1000), iNOS (ab3523, 1:1000) were from Abcam. Antibody for F4/80 (sc-377009, 1:200) was from Santa Cruz Biotechnology. Secondary antibodies HRP-conjugated goat anti-rabbit (ZB-2301, 1:5000), HRP-conjugated goat anti-mouse (ZB-2305, 1:5000), goat anti-rabbit (Alexa Fluor 594, ZF-0516, 1:200), goat anti-rabbit (Alexa Fluor 488, ZF-0511, 1:200), goat anti-mouse (Alexa Fluor 488, ZF-0512, 1:200) were Purchased from Zhongshan Golden Bridge Biotechnology.

**Cells**

Bone marrow-derived macrophages (BMDMs) were generated from 6–8-week-old C57BL/6 mice. Bone marrow cells were flushed from femurs and tibiae with phosphate-buffered saline (PBS), passed through a 70 μm cell strainer, and cultured for 7 days in RPMI-1640 medium supplemented with 10% fetal bovine serum (FBS), 1% penicillin-streptomycin, and 20 ng/mL recombinant murine macrophage colony-stimulating factor (M-CSF; PeproTech) to induce differentiation. The culture medium was replaced with fresh medium on days 3 and 5. Adherent BMDMs were harvested on day 7 for subsequent experiments.

Murine macrophage-like RAW264.7 cells were obtained from the American Type Culture Collection (ATCC) and maintained in DMEM supplemented with 10% FBS and 1% penicillin-streptomycin. Cells were cultured at 37°C under 5% CO₂ and subcultured at 70–80% confluence for experimental use.

Human umbilical vein endothelial cells (HUVECs) were cultured in Dulbecco’s Modified Eagle Medium (DMEM) supplemented with 10% FBS and 1% penicillin-streptomycin at 37°C in a humidified 5% CO₂ atmosphere. HUVECs between passages 3 and 6 were utilized for experiments.

Murine fibroblast L929 cells were cultured in DMEM containing 10% FBS and 1% penicillin-streptomycin at 37°C in a humidified incubator with 5% CO₂. L929 cells were passaged every 2–3 days using standard trypsinization and were employed between passages 5 and 15 for experiments.

**Animals**

Male C57BL/6 wild-type (WT) mice and STING conditional knockout mice were obtained from Chongqing Medical University Experimental Animal Center and Shanghai Biomodel Organism Science & Technology Development Co., Ltd., respectively. All mice were maintained under specific pathogen-free (SPF) conditions with a 12 h light/dark cycle and *ad libitum* access to chow and water. Animal experiments were approved by the Institutional Animal Care and Use Committee (IACUC) of Chongqing Medical University (Approval No. 2025-096) and conducted in accordance with the NIH Guide for the Care and Use of Laboratory Animals. Experimental group sizes are detailed in the figure legends.

**Bioinformatic analysis**

Clinicopathological and transcriptomic data from three independent Gene Expression Omnibus (GEO) datasets—sepsis-induced lung injury (GSE270838), periodontitis (GSE223924), and psoriatic skin lesions (GSE205748)—were integrated^1–3^. mRNA expression was quantified using R (v3.5.1), and *TMEM173* expression was visualized via violin plots. Gene expression correlations were analyzed with correlation coefficients plotted using the *corrplot* package. Intersecting differentially expressed genes (DEGs) underwent functional enrichment analysis via DAVID, focusing on Kyoto Encyclopedia of Genes and Genomes (KEGG) pathways to identify *TMEM173*-associated biological processes. In addition, single-cell RNA sequencing data from GSE190856 were analyzed using the Seurat package to characterize immune cell heterogeneity in sepsis^4^. Dimensionality reduction was performed with UMAP, and cell-type annotations were used to quantify immune cell subsets and their relative proportions under septic conditions. TMEM173 expression was further mapped across clusters to pinpoint the predominant cellular sources of STING signaling within the immune microenvironment.

**Human specimens**

This study utilized clinical tissues including: (1) lung samples from deceased acute respiratory distress syndrome (ARDS) patients with sepsis-induced respiratory failure, with matched controls from histologically normal regions distal to tumor sites in lobectomy patients (confirmed infection/inflammation-free); (2) gingival tissues from chronic periodontitis patients undergoing periodontal surgery/tooth extraction, alongside healthy controls from age-matched individuals receiving crown-lengthening procedures without periodontal disease; and (3) cutaneous fistula tissues from congenital preauricular fistulas with recurrent infection/inflammation confirmed histologically, with control skins from anatomically matched sites during cosmetic auricular surgery in patients lacking infection/inflammation/congenital defects history. All samples were collected anonymously or with written informed consent from participants/legal surrogates per Declaration of Helsinki principles. Protocols were approved by Institutional Review Boards of the First Affiliated Hospital (Lung; No.2020-147-2 / Skin; No.2024-324-01), Affiliated Stomatological Hospital (Gingiva; No.2025-097).

**STING function in inflammatory models**

To interrogate the role of STING in inflammatory responses, three murine disease models were established in wild-type (WT) and STING knockout (SKO) mice. Sepsis was induced by intraperitoneal (i.p.) injection of lipopolysaccharide (LPS, 10 mg/kg)^5,6^. Survival was monitored for 48 h by Kaplan–Meier analysis, followed by collection of tissues and blood (groups: WT/Saline, WT/LPS, SKO/Saline, SKO/LPS). Periodontitis was induced by ligation of the maxillary second molar with 5-0 silk sutures for 14 days (groups: WT/Saline, WT/Ligature, SKO/Saline, SKO/Ligature)^7^. Inflammatory skin wounds were generated by 7 mm full-thickness dorsal excisions, followed by topical LPS (200 ng/mL, 20 µL)^8^. Wound healing was digitally monitored for 14 days (groups: WT/Saline, WT/LPS, SKO/Saline, SKO/LPS).

**Immunofluorescence staining**

Human tissues were cryosectioned at 5 μm after OCT embedding, fixed in 4% paraformaldehyde, permeabilized with 0.3% Triton X-100, and blocked with 5% BSA. Sections underwent overnight incubation with primary antibodies at 4°C, followed by secondary antibody staining and DAPI nuclear counterstaining, with imaging performed via confocal microscopy.

Mouse tissues were processed as paraffin sections; after deparaffinization, rehydration, and antigen retrieval in citrate buffer (pH 6.0), sections were permeabilized, blocked, and stained identically with primary/secondary antibodies and DAPI. Images were acquired using a Leica SP8 laser scanning confocal microscope.

**Immunocytochemistry (ICC)**

Cells were cultured on glass coverslips and subjected to designated treatments. After fixation with 4% paraformaldehyde for 15 min, cells were permeabilized using 0.1% Triton X-100 for 10 min and blocked in 5% BSA for 1 h at room temperature. Cells were then incubated with primary antibodies overnight at 4 °C, followed by fluorescent secondary antibodies for 1 h at room temperature. Nuclei were counterstained with DAPI, and images were captured using a laser scanning confocal microscope (Leica SP8).

**siRNA Transfection**

Mouse TSG101-specific siRNA and mouse LC3-specific siRNA were purchased from GenePharma Co., Ltd. and BMDM cells were transfected using Lipofectamine RNAiMAX following the manufacturer’s protocol.

**Western blot**

Tissues or cells were lysed in RIPA buffer supplemented with protease and phosphatase inhibitors. Protein concentration was determined using the BCA assay. Equal amounts of protein were separated by SDS-PAGE and transferred to PVDF membranes. After blocking with 5% non-fat milk, membranes were incubated with primary antibodies overnight at 4 °C, followed by HRP-conjugated secondary antibodies. Signals were visualized using enhanced chemiluminescence (ECL) and imaged with ChemiDoc^TM^ MP Imaging System (Bio-Rad).

**Quantitative Real-Time PCR (qRT-PCR)**

Total RNA was extracted from cells using Trizol reagent (Thermo Fisher Scientific) following the manufacturer’s protocol. RNA concentration and purity were assessed by NanoDrop spectrophotometry. Reverse transcription was performed using a PrimeScript RT reagent kit (Takara) to synthesize cDNA. Quantitative real-time PCR was carried out using TB Green Premix Ex Taq II (Takara) on a ProFlex PCR system (Thermo Fisher). Gene expression levels were normalized to GAPDH and calculated using the 2^–ΔΔCt^ method. Primer sequences are provided in Supplementary Table S1.

**Table S1: Sequence of Primers for q-PCR.**

| Genes | Forward Primer (5’-3’) | Reverse Primer (5’-3’) |
| --- | --- | --- |
| IFNβ | CACAGCCCTCTCCATCA | TCTCCGTCATCTCCATAGG |
| ISG20 | TCACGGACTACAGAACCCA | ACCACCAGCTTGCCTTT |
| IL-1β | TGAATTGGTCATAGCCCGCA | TCTCCTTCCTGTGCAAACTCT |
| iNOS | GGCCACCAAGCTGAACTTGA | GTTCCAGCTTCTGGCACTGA |
| TNFα | CTGAACTTCGGGGTGATCGG | GGCTTGTCACTCGAATTTTGAGA |
| TMEM173 | CAAGAGCCAAGACTCCTCA | CTGTCCCATGCCTCAGAT |
| GAPDH | TGAGGTGACCGCATCTTCTTG | TGGTAACCAGGCGTCCGATA |

**Compound synthesis**

Reactions were monitored by analytical thin-layer chromatography (TLC) on F254 precoated plates of silica gel with spot detection under UV light at 254 nm. Column chromatography was carried out by using silica gel (200-300 mesh) packed in a glass column. 1H NMR and 13C NMR spectra were obtained on an Agilent 400 MHz spectrometer. The chemical shifts were reported in parts per million (ppm), the coupling constants (J) were expressed in hertz (Hz) and signals were described as singlet (s), doublet (d), triplet (t), as well as multiplet (m). The NMR data was analyzed by MestReNova software. High-resolution mass spectra were obtained on Agilent 6546. The detailed synthetic procedure of the ATTEC small molecules is provided in the Supplementary Synthesis and Characterization of STING-ATTEC Variants.

**Nanoparticle preparation**

Lipid nanoparticles (LNPs) were prepared by the ethanol injection method. For standard LNPs, D-Lin-MC3-DMA, DSPC, cholesterol, and DSPE-PEG2000 were mixed at a molar ratio of 50:10:38.5:1.5. For folic acid-modified LNPs (FA-LNPs), DSPE-PEG2000 was replaced with DSPE-PEG-Folate at the same molar proportion. To prepare positively charged FA-LNPs (FA-LNP⁺), DOTAP was introduced into the lipid mixture at different molar ratios, as follows: (1) DOTAP:D-Lin-MC3-DMA: DSPC: cholesterol: DSPE-PEG-Folate = 25:25:10:38.5:1.5, (2) 20:30:10:38.5:1.5, (3) 15:35:10:38.5:1.5, (4) 10:40:10:38.5:1.5 and (5) 5:45:10:38.5:1.5. All lipid components were dissolved in an appropriate volume of anhydrous ethanol according to the specified molar ratios. The ethanol phase was then rapidly injected into prewarmed phosphate-buffered saline (PBS) at 60 °C, with ethanol accounting for 20% of the total volume (v/v). The mixture was stirred for 1 h at 60 °C using a thermostatic magnetic stirrer to allow complete hydration. Finally, ethanol was removed by rotary evaporation under reduced pressure in a 40 °C water bath, yielding the final LNP solution.

**Molecular docking simulations**

Small-molecule ligands were prepared using the LigPrep module of Maestro (release 2015-2, Schrödinger Inc.), with energy minimization performed under the OPLS3 force field. Protonation states were generated with Epik at physiological pH (7.0 ± 2.0). The three-dimensional structures of STING (PDB ID: 6NT5) and LC3 (PDB ID: 6J04) were obtained from the Protein Data Bank (http://www.rcsb.org/pdb). For LC3, chain D was extracted as the monomer. Protein structures were preprocessed with the Protein Preparation Wizard (Schrödinger 2015-2) to remove water molecules, ions, and non-essential ligands, followed by the addition of missing side chains and loops. Protonation states were assigned using Epik (pH 7.0 ± 2.0), hydrogen bonding networks were optimized using PROPKA (pH 7.0), and structures were subsequently minimized with the OPLS3 force field until heavy atoms converged to an RMSD of 0.30 Å.

Protein–protein docking between STING and LC3 was carried out using ZDOCK with default parameters, and the top-ranked complex consistent with previous reports was selected for subsequent modeling. The resulting STING–LC3 complex was further refined with the Protein Preparation Wizard. Flexible molecular docking of ligands into the STING–LC3 complex was then performed using the Induced Fit Docking protocol in Schrödinger, with the docking grid centered on residue Cys91 of STING chain A. Ligand conformational sampling was carried out within an energy window of 2.5 kcal/mol. During the Glide docking stage, van der Waals radii of both ligands and receptor atoms were scaled by a factor of 0.50. Local side chains within 5.0 Å of the ligand poses were minimized using Prime refinement, followed by redocking within a 30.0 kcal/mol energy cutoff relative to the top-scoring conformation. Final docking poses were ranked using the standard precision scoring function.

**In vitro pull-down assay**

Recombinant human LC3B protein with a C-terminal His tag (His-LC3B) and recombinant human STING protein were purchased from Sino Biological and MCE respectively. Ni-NTA agarose beads were obtained from QIAGEN. For each reaction, 2 μg recombinant STING protein and 2 μg His-LC3B were incubated in 500 μL binding buffer (20 mM Tris-HCl, pH 7.4, 150 mM NaCl, 0.2% NP-40, 1 mM DTT, and protease inhibitor cocktail) in the presence of STING-ATTEC (10 μM) or an equal volume of DMSO as control. The mixtures were incubated at 4°C for 1 h with gentle rotation to allow complex formation. Subsequently, 30 μL pre-equilibrated Ni-NTA agarose beads were added to each reaction and incubated for an additional 1 h at 4°C with rotation to capture His-LC3B. The beads were then washed four times with 1 mL binding buffer to remove unbound proteins. Bound proteins were eluted by boiling the beads in 2× SDS sample buffer at 95°C for 5 min and subjected to SDS-PAGE followed by Western blot analysis. Membranes were probed with antibodies against STING and LC3 to detect the presence of STING in the pull-down complex. For input controls, 10% of the reaction mixture was collected before bead incubation and analyzed in parallel by immunoblotting.

**Co-immunoprecipitation assay**

Co-IP was performed to determine the interaction between endogenous STING and LC3B in BMDMs. BMDMs were lysed in ice-cold IP lysis buffer (20 mM Tris-HCl, pH 7.4, 150 mM NaCl, 1% NP-40, 1 mM EDTA) supplemented with protease inhibitor cocktail. The cell lysates were incubated on ice for 30 min and clarified by centrifugation at 12,000 × g for 15 min at 4°C. For immunoprecipitation, equal amounts of total protein (500–800 μg) were incubated overnight at 4°C with 2 μg anti-STING antibody with gentle rotation. Subsequently, 30 μL Protein A/G agarose beads were added and incubated for an additional 2 h at 4°C. The beads were washed four times with cold lysis buffer to remove nonspecific binding proteins. Bound protein complexes were eluted by boiling the beads in 2× SDS loading buffer at 95°C for 5 min. The immunoprecipitated samples were separated by SDS-PAGE and transferred to PVDF membranes for Western blot analysis. Membranes were probed with antibodies against LC3B to detect LC3 associated with STING. For input controls, 5–10% of the total cell lysates were collected prior to immunoprecipitation and analyzed by immunoblotting using antibodies against STING, LC3B, and β-tubulin as a loading control.

**Proteomics analysis**

BMDMs were used for quantitative proteomic profiling under two experimental settings. For the first comparison, cells were pretreated with STING-ATTEC (10 µM) for 6 h, followed by LPS stimulation (100 ng/mL, 6 h); control cells were stimulated with LPS alone (100 ng/mL, 6 h). For the second comparison, cells were treated with FA-LNP⁺ (50 µg/mL, 3 h) or FA-LNP (50 µg/mL, 3 h). After treatment, cells were harvested, snap-frozen in liquid nitrogen, and stored at −80 °C until analysis. Protein samples were submitted to Novogene Co. Ltd (Beijing, China) for data-independent acquisition (DIA)-based quantitative proteomics using the Astral DIA platform. Raw data were processed on the NovoMagic Plus cloud platform, and subsequent bioinformatic analyses, including differential expression, volcano plots, and gene set enrichment analysis (GSEA), were performed using R. Differentially expressed proteins (DEPs) were defined as those with a fold change ≥2 or ≤0.5 and a P value <0.05.

**Surface Plasmon Resonance**

SPR analysis was performed using a Biacore 8k system (Cytiva) with a CM5 sensor chip (lot: 10351489). Recombinant human TMEM173 protein (N-His-SUMO, HY-P70700A, MCE) was immobilized via amine coupling after pH scouting, with pH 4.0 yielding optimal conjugation efficiency. The final immobilization level exceeded 14,000 RU. STING-ATTEC was injected at a series of concentrations (0, 0.39, 0.78, 1.56, 3.125, 6.25, 12.5, 25, 50 μM) with a flow rate of 30 μL/min, an association time of 120 s, and a dissociation time of 180 s. A 50% DMSO solution was used for chip regeneration. Kinetic parameters were calculated using Biacore evaluation software with a standard 1:1 binding model.

**Circular Dichroism (CD) Spectroscopy**

CD spectroscopy was performed using a Bio-Logic MOS-450 spectropolarimeter to assess the secondary structural changes of the STING (TMEM173) protein upon STING-ATTEC binding. The recombinant human STING protein (N-His-SUMO-tagged, 50 μg/mL) was dissolved in PBS buffer (pH 7.4), and measurements were conducted at 37 °C using a 10 mm pathlength quartz cuvette. After baseline recording, STING-ATTEC was added to a final concentration of 50 μM and incubated with the protein for 2 h at 37 °C. Spectra were acquired in the range of 190–260 nm, with a scan speed of 100 nm/min and a bandwidth of 1.0 nm. Buffer baselines were subtracted, and each spectrum was obtained by averaging three scans.

**Transmission Electron Microscopy (TEM)**

TEM was used to assess nanoparticle morphology and cellular ultrastructure. LNP@STING-ATTEC, FA-LNP@STING-ATTEC, and FA-LNP⁺@STING-ATTEC samples were deposited on carbon grids, negatively stained with 2% phosphotungstic acid, and imaged to evaluate particle shape and size. For cellular analysis, BMDMs treated with FA-LNP or FA-LNP⁺ were fixed, embedded, sectioned, and stained with uranyl acetate and lead citrate. Autophagosomes and other subcellular structures were observed under Tecnai G2 20 TWIN (FEI).

**Dynamic Light Scattering (DLS)**

The hydrodynamic size of LNP@STING-ATTEC, FA-LNP@STING-ATTEC, and FA-LNP⁺@STING-ATTEC was measured using dynamic light scattering (DLS) on a Brookhaven 90Plus analyzer. Samples were diluted in deionized water and analyzed at 25 °C. Size distribution and average particle diameter were recorded.

**Flow Cytometry**

To investigate the cellular uptake mechanism of FA-LNPs, BMDMs were pretreated with various endocytic pathway inhibitors for 12 h, including Cytochalasin D, Chlorpromazine, Indomethacin, Colchicine, Wortmannin, and MβCD. After pretreatment, cells were incubated with Cy5-labeled FA-LNPs for 3 h. Control and FA-LNP-only groups were included. Cells were then washed thoroughly with PBS, harvested, and analyzed by CytoFLEX (Beckman Coulter) to quantify Cy5 fluorescence intensity, indicating FA-LNP internalization.

**Therapeutic evaluation of FA-LNP formulations**

To assess therapeutic efficacy, WT mice were subjected to the same models and treated with FA-LNP formulations. In the sepsis model, FA-LNP⁺, FA-LNP⁺@C170, or FA-LNP⁺@STING-ATTEC were administered i.p. at 2 mg/kg, 1 h prior to LPS challenge (groups: Saline, LPS, LPS+FA-LNP⁺, LPS+FA-LNP⁺@C170, LPS+FA-LNP⁺@STING-ATTEC, LPS+Dexamethasone [5 mg/kg]). In the periodontitis model, gingival tissues received local injection of FA-LNP formulations (50 µg in 20 µL PBS) every three days for 14 days (groups: Saline, Ligature+Saline, Ligature+FA-LNP⁺, Ligature+FA-LNP⁺@C170, Ligature+FA-LNP⁺@STING-ATTEC, Ligature+Periocline®). In the inflammatory wound model, wounds were topically treated with FA-LNP formulations (100 µg in 50 µL PBS) once daily for 14 days (groups: Saline, Wound+Saline, Wound+FA-LNP⁺, Wound+FA-LNP⁺@C170, Wound+FA-LNP⁺@STING-ATTEC, Wound+Tegaderm^3M^). Tissues were collected at experimental endpoints for histological and molecular analyses, while wound closure was quantified using ImageJ.

**Micro-CT Analysis**

The harvested alveolar bones were fixed in 4% paraformaldehyde and subsequently scanned using a vivaCT80 micro-CT system (SCANCO Medical AG, Switzerland). The scanning was performed at 70 kV and 112 μA with an isotropic voxel size of 10 μm. A cuboidal region of interest (ROI) encompassing the alveolar bone was defined, extending from the most distal aspect of the upper first molar (M1) root to the most mesial aspect of the upper second molar (M2) root in length, from the most buccal to the most palatal aspect of the M1/M2 roots in width, and from the most apical portion of the root to the alveolar bone crest (ABC) in height. Quantitative parameters including the cementoenamel junction to alveolar bone crest distance (CEJ–ABC), bone volume per tissue volume (BV/TV), and trabecular thickness (Tb. Th) were calculated using SCANCO VivaCT software.

**Hematoxylin and Eosin (H&E) Staining**

Tissues from three disease models—including lung (sepsis), alveolar bone (periodontitis), and skin (inflammatory wound)—were harvested and fixed in 4% paraformaldehyde overnight. After dehydration and paraffin embedding, samples were sectioned at 5 μm thickness and stained with hematoxylin and eosin following standard protocols. Histopathological features were assessed under a light microscope.

**Tartrate-resistant acid phosphatase (TRAP) staining**

The alveolar bones were decalcified, paraffin-embedded, and sectioned at a thickness of 5 μm. TRAP staining was performed using a commercially available kit (Servicebio, # G1050-50T, China) according to the manufacturer’s instructions to visualize osteoclasts. TRAP-positive multinucleated cells located along the alveolar bone surface were counted under a light microscope. Quantification was conducted in defined regions of interest surrounding the upper first molar (M1), and results were expressed as the number of TRAP-positive cells per bone surface area.

**Immunohistochemistry (IHC)**

Alveolar bone specimens were decalcified in 10% EDTA (pH 7.4) at room temperature for 3 weeks, dehydrated, and embedded in paraffin. Serial sections (5 μm) were cut, deparaffinized, and subjected to antigen retrieval using citrate buffer (pH 6.0) in a microwave oven. Endogenous peroxidase activity was quenched with 3% hydrogen peroxide, and non-specific binding was blocked with 5% BSA for 30 min. Sections were incubated with primary antibodies overnight at 4 °C, followed by HRP-conjugated secondary antibodies. DAB substrate was used for color development, and hematoxylin was used for counterstaining. Images were acquired using a bright-field microscope.

**Masson’s Trichrome Staining**

Skin tissue samples from the inflammatory wound model were fixed in 4% paraformaldehyde, embedded in paraffin, and sectioned at 5 μm thickness. Sections were stained using Masson’s trichrome staining kit according to the manufacturer’s instructions, to evaluate collagen deposition and tissue remodeling. Collagen fibers were visualized as blue, muscle and cytoplasm as red, and nuclei as dark brown under light microscopy.

**Statistical Analysis**

All data are presented as mean ± standard deviation (SD). Statistical comparisons between two groups were performed using unpaired two-tailed Student’s *t* test, while comparisons among multiple groups were analyzed by one-way analysis of variance (ANOVA). Statistical analysis was conducted using GraphPad Prism 9.0 (GraphPad Software, USA). A p-value < 0.05 was considered statistically significant.

**Code Availability**

The analysis scripts used in this study, including those for single-cell data processing and downstream analyses, have been deposited in a publicly accessible GitHub repository and are available at: https://github.com/dentistzfy-art/Bioinformatics-Analysis.

**Supplementary Figures**


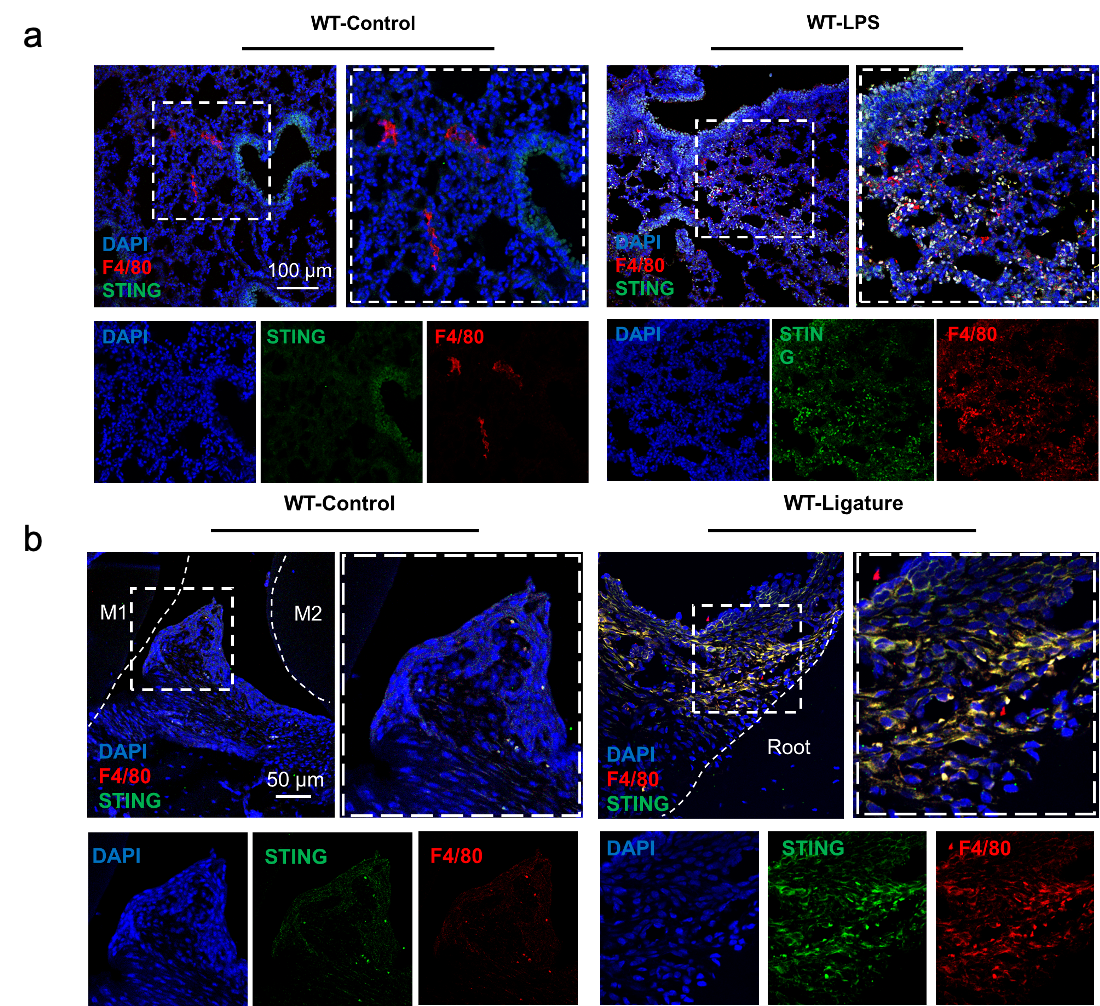


**Supplementary Fig. 1** Representative immunofluorescence staining of mouse tissues. (a) Lung sections from septic mice and (b) alveolar bone sections from periodontitis mice were stained for STING (green), F4/80 (red), and DAPI (blue) to visualize STING expression and macrophage distribution in inflammatory microenvironments.


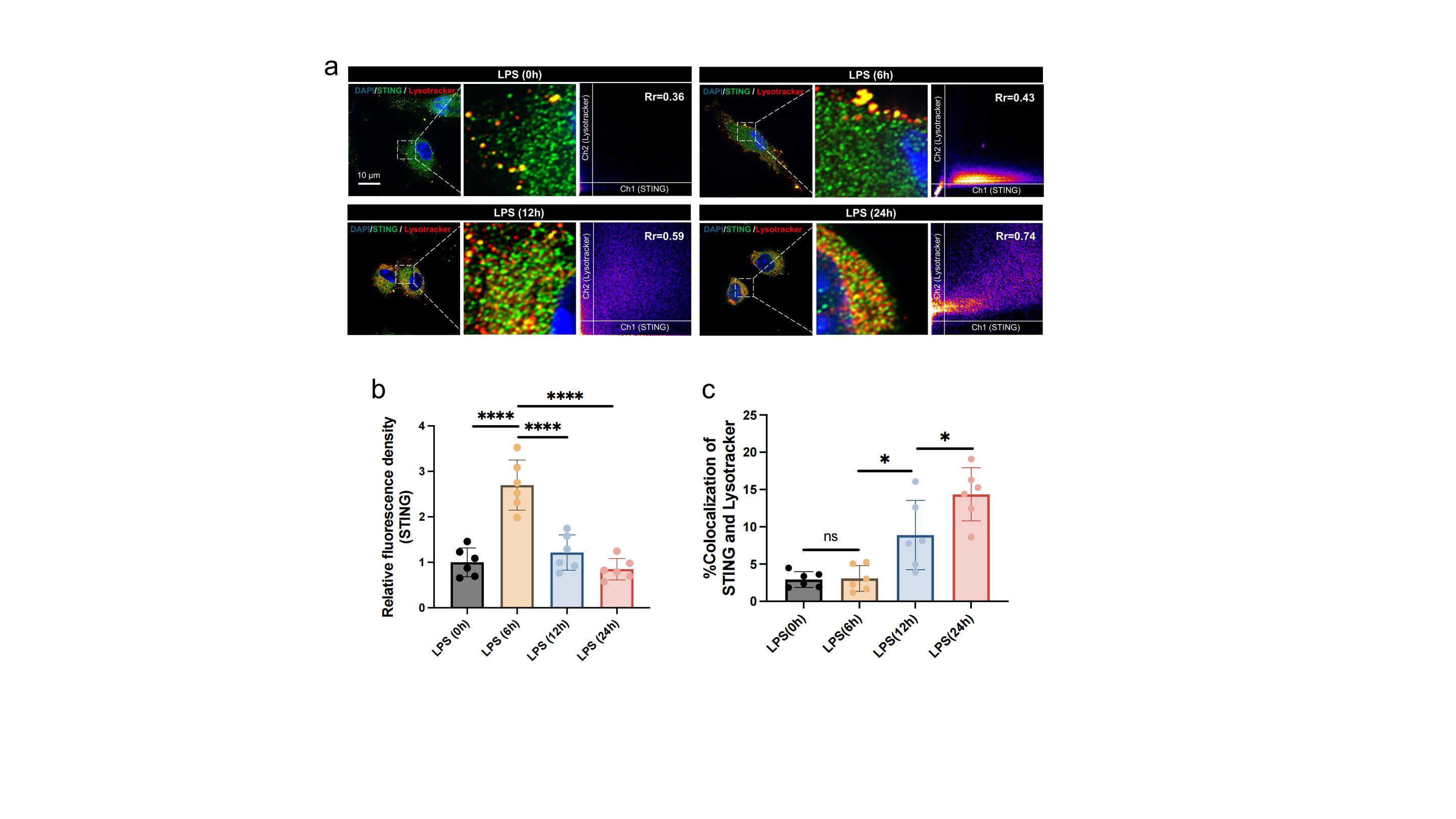


**Supplementary Fig. 2** (a) Representative immunofluorescence images of STING and Lysotracker in BMDMs at given time points following LPS stimulation. (b, c) Semi-quantitative analysis of immunofluorescence images in LPS (0h), LPS (6h), LPS (12h), and LPS (24h) groups, showing (b) relative fluorescence intensity of STING and (c) percentage colocalization of STING with LysoTracker (n=6, per group).


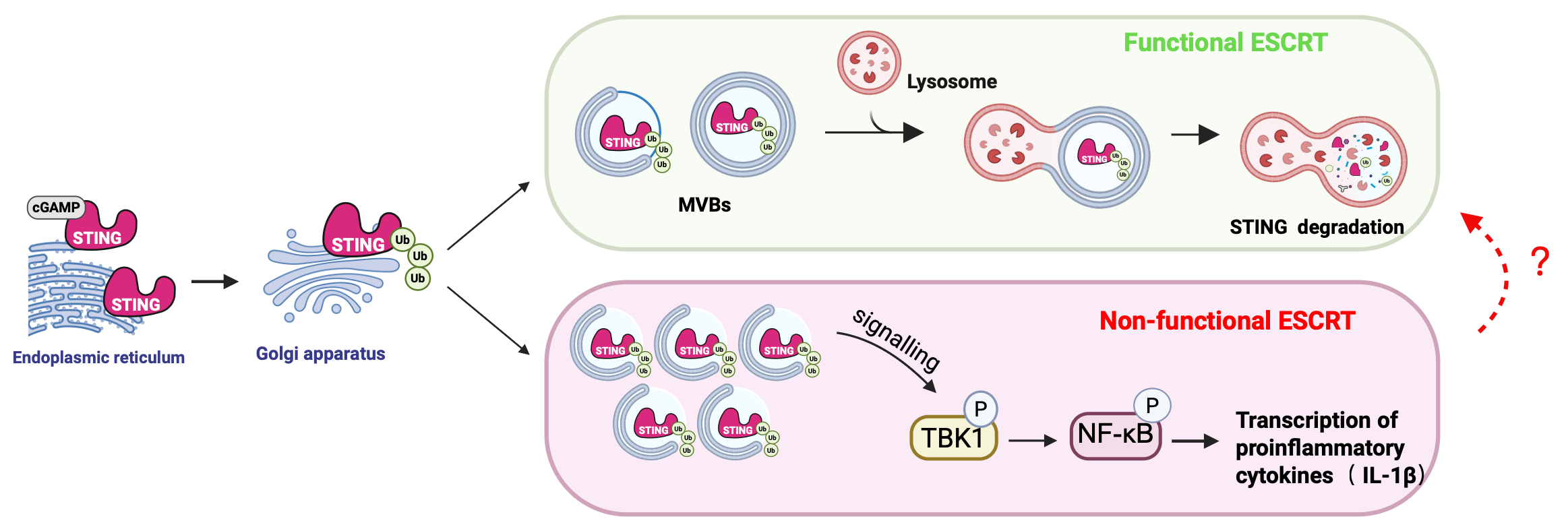


**Supplementary Fig. 3** Schematic illustration of STING protein degradation pathways. STING degradation can proceed through either functional ESCRT, leading to normal lysosomal clearance, or non-functional ESCRT, which fails to degrade STING and instead triggers downstream inflammatory cascades. The potential conversion of non-functional ESCRT into functional ESCRT remains an open question.


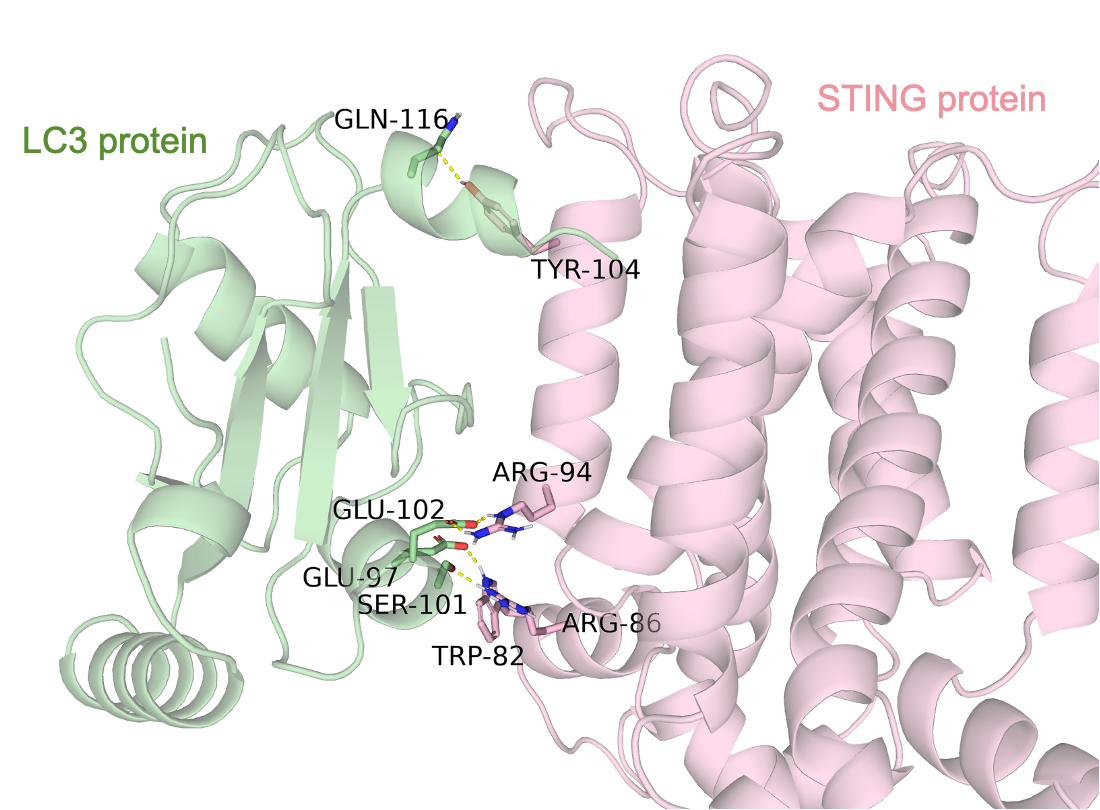


**Supplementary Fig. 4** Docking model of the ternary STING–LC3–STING-ATTEC complex. STING (pink), LC3 (green), and the STING-ATTEC small molecule (stick representation) are shown, with key hydrogen bond interactions highlighted.


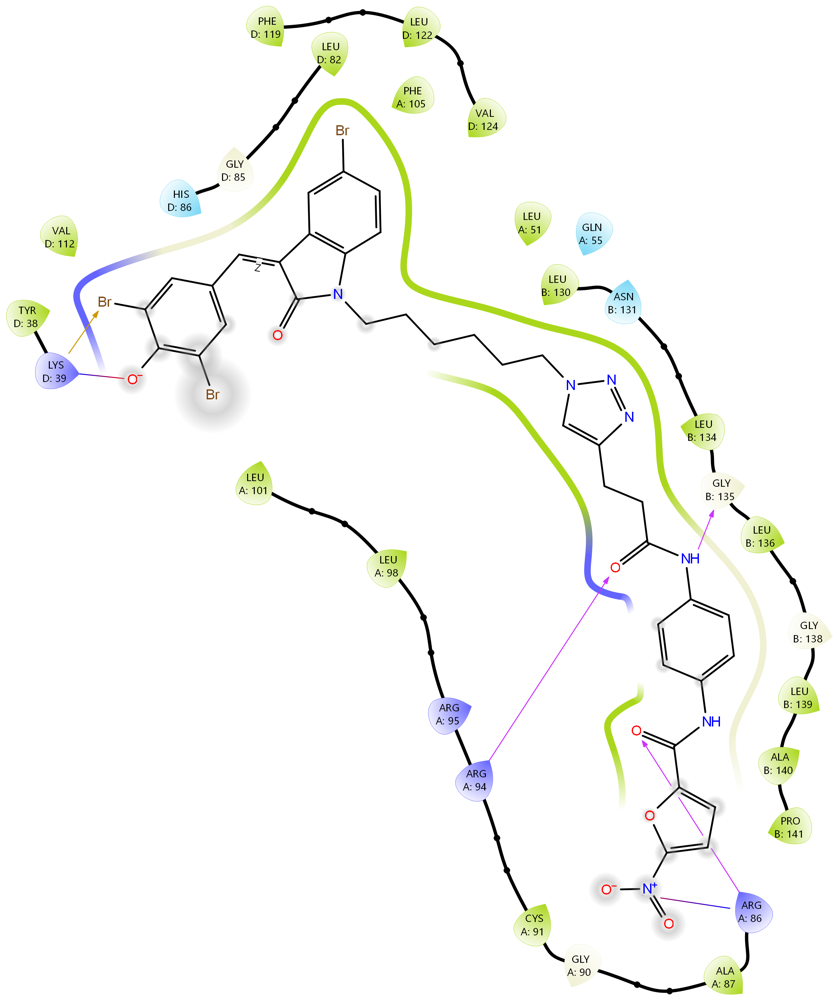


**Supplementary Fig. 5** Two-dimensional molecular docking interaction map of STING-ATTEC within the STING–LC3 complex. Key hydrogen bonds, hydrophobic contacts, and halogen interactions with STING and LC3 residues are shown, illustrating stable dual engagement of both warheads.





**Supplementary Fig. 6** CD measurements of TMEM173 protein mixed with STING-ATTEC.


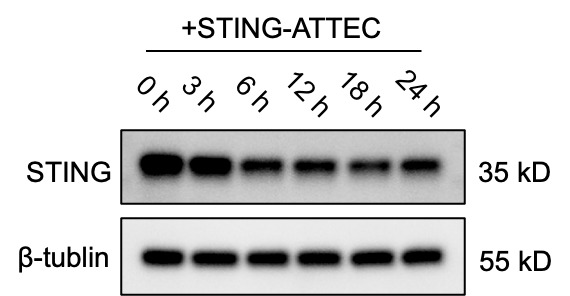


**Supplementary Fig. 7** Time-course analysis of STING degradation after exposure to the STING-ATTEC (0–24 h) in BMDMs.


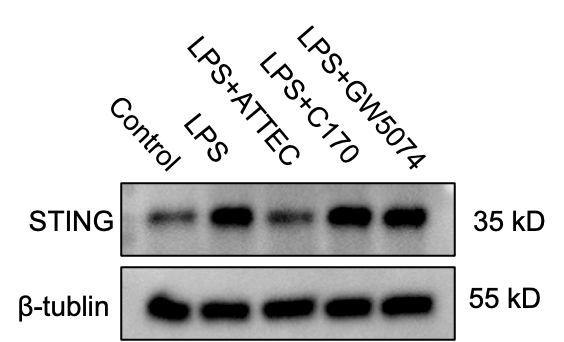


**Supplementary Fig. 8** Western blot analysis of STING protein expression in control, LPS-treated, LPS + ATTEC–treated, LPS + C-170–treated, and LPS + GW5074–treated groups.

**Supplementary Fig. 9** qRT-PCR analysis of TMEM173 mRNA expression in control, LPS-treated, LPS + C-170–treated, and LPS + STING-ATTEC–treated groups (n=3, per group).


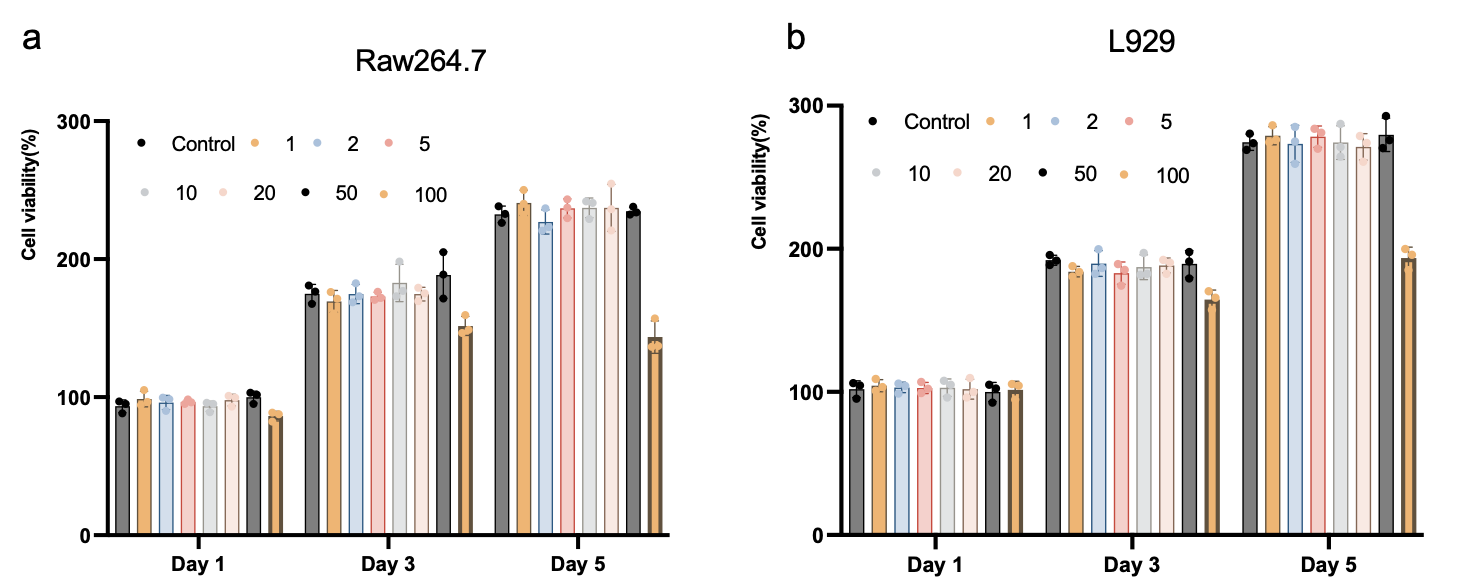


**Supplementary Fig. 10** CCK-8 assay evaluating the viability of RAW264.7 (a) and L929 (b) cells following treatment with STING-ATTEC at concentrations ranging from 1 to 100 µmol, measured at 1, 3, and 5 days to assess biocompatibility (n=3, per group).


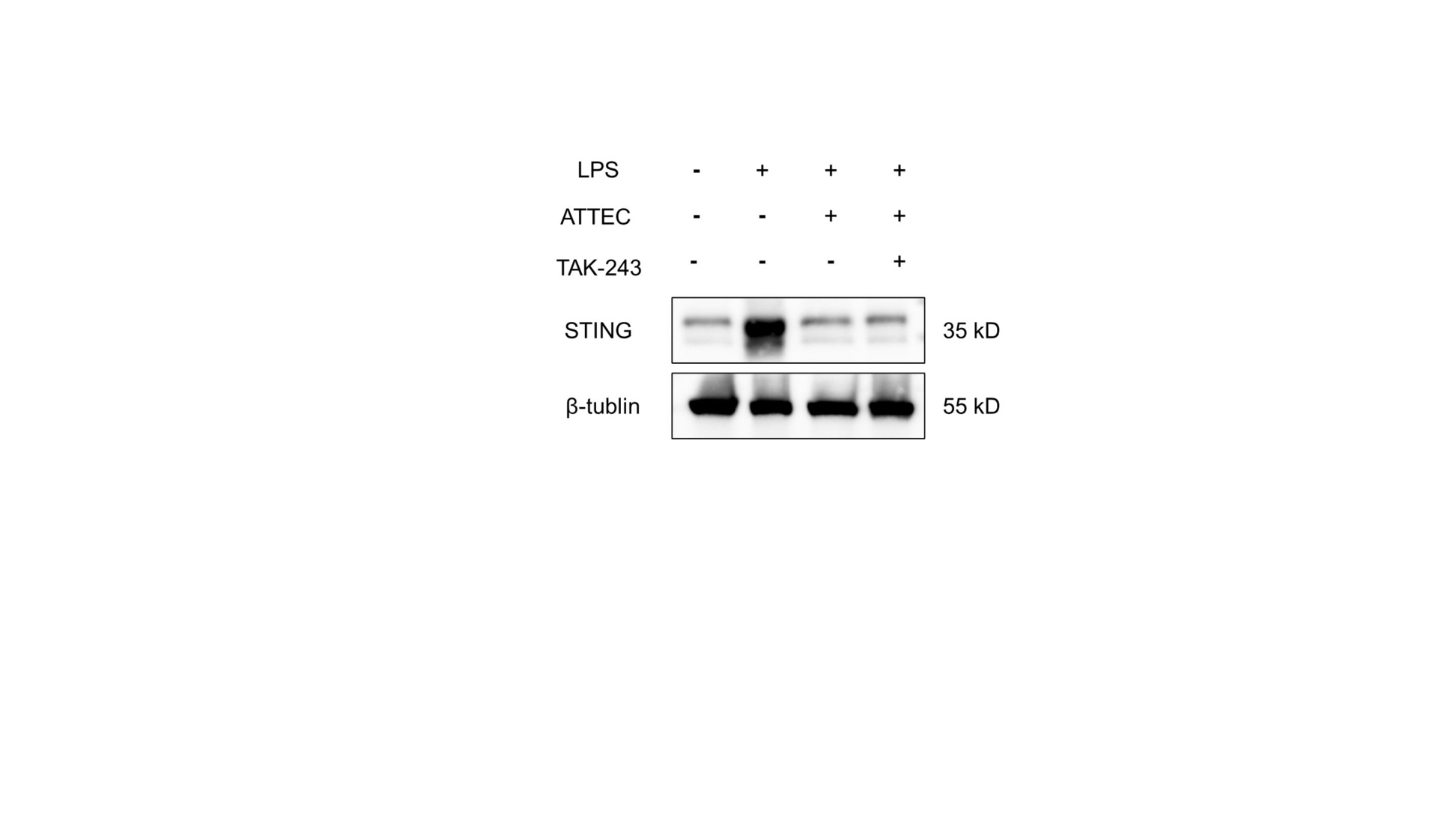


**Supplementary Fig. 11** Western blot analysis of STING degradation in BMDMs treated with TAK-243 and STING-ATTEC.


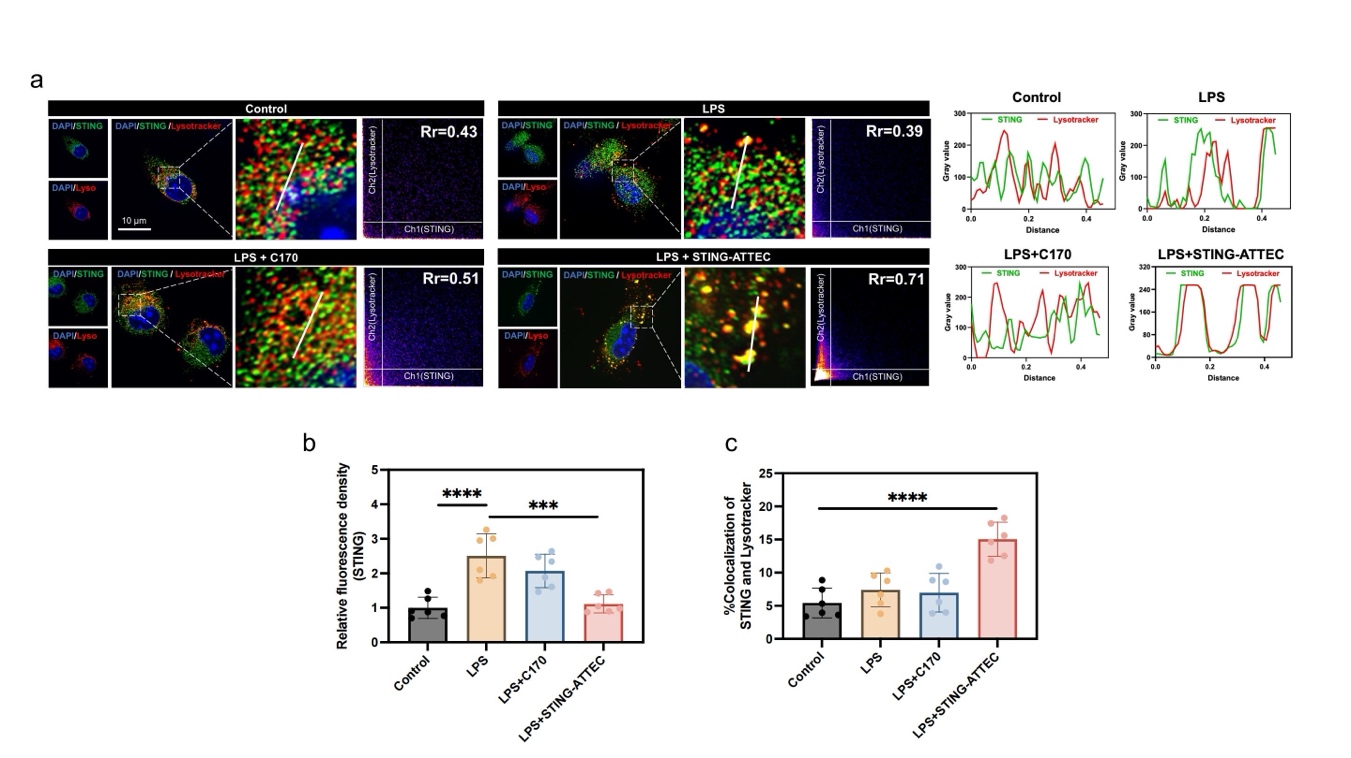


**Supplementary Fig. 12** (a) Immunofluorescence staining of STING and lysosomes in BMDMs under indicated treatments after LPS stimulation for 6 h. (b, c) Semi-quantitative analysis of immunofluorescence images in control, LPS-treated, LPS + C-170–treated, and LPS + STING-ATTEC–treated groups, showing (b) relative fluorescence intensity of STING and (c) percentage colocalization of STING with LysoTracker (n=6, per group).


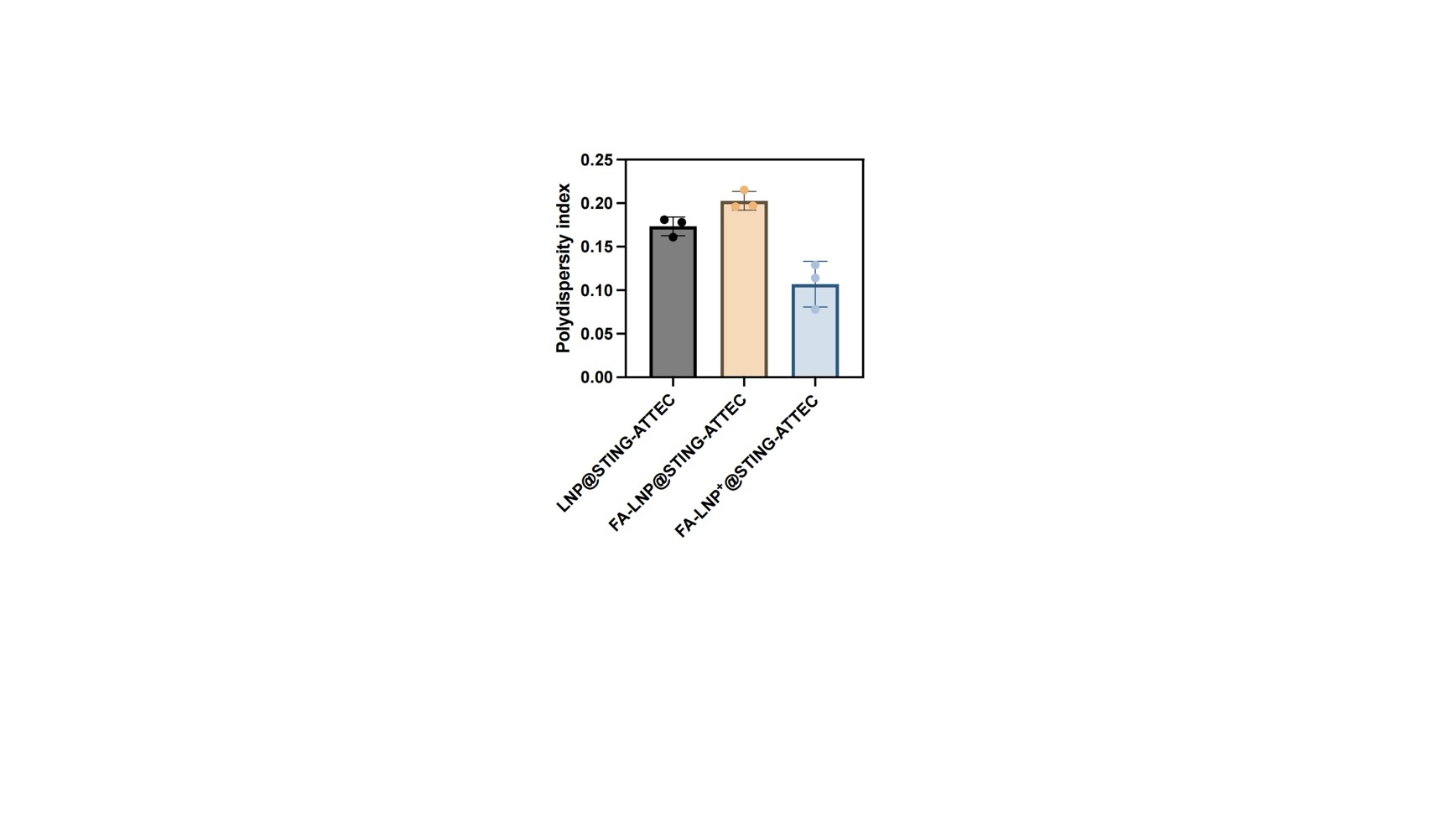


**Supplementary Fig. 13** PDI of nanoparticles measured by dynamic light scattering (n=3, per group).


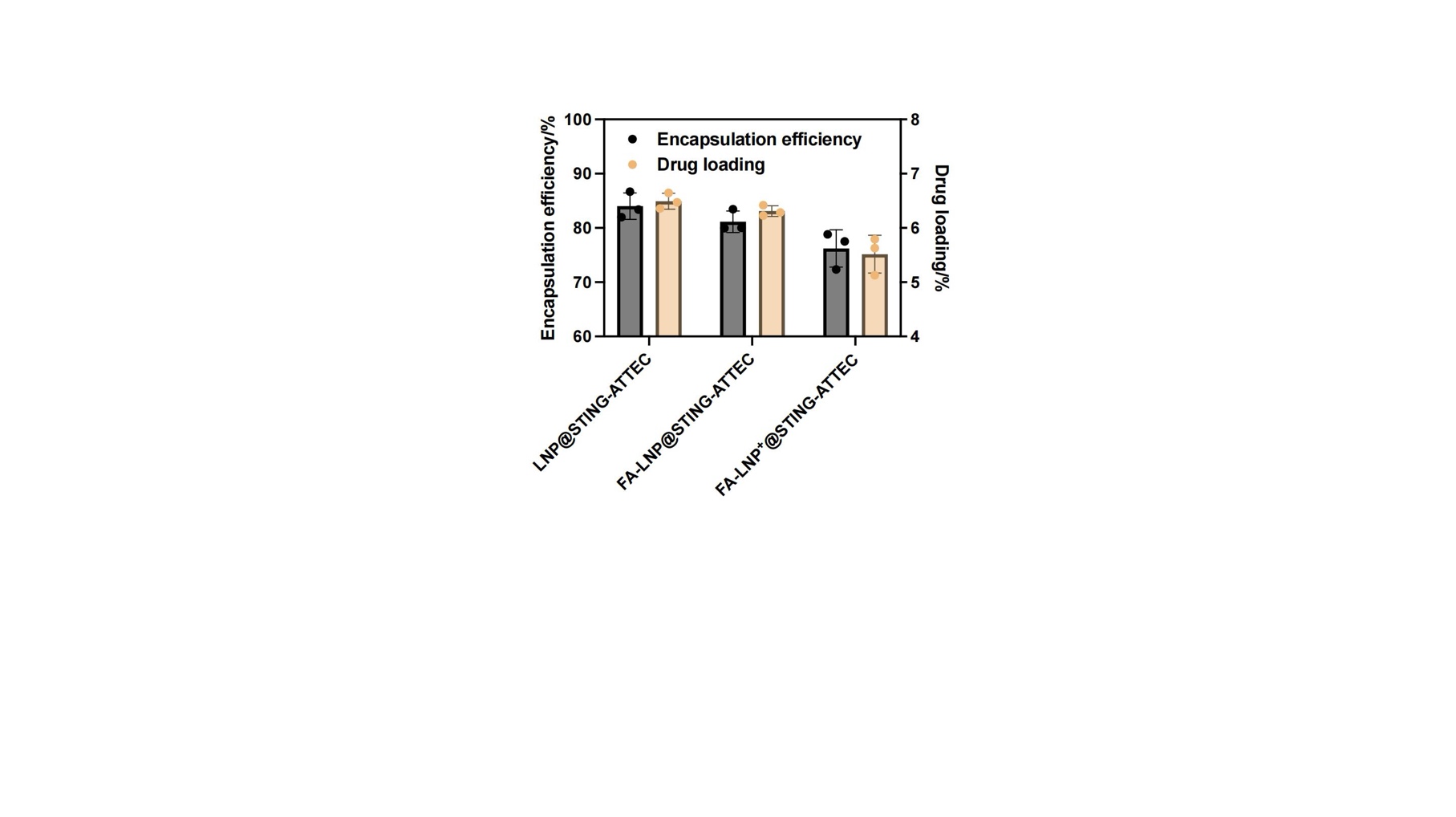


**Supplementary Fig. 14** Encapsulation efficiency and drug loading of different nanoparticles (n=3, per group).


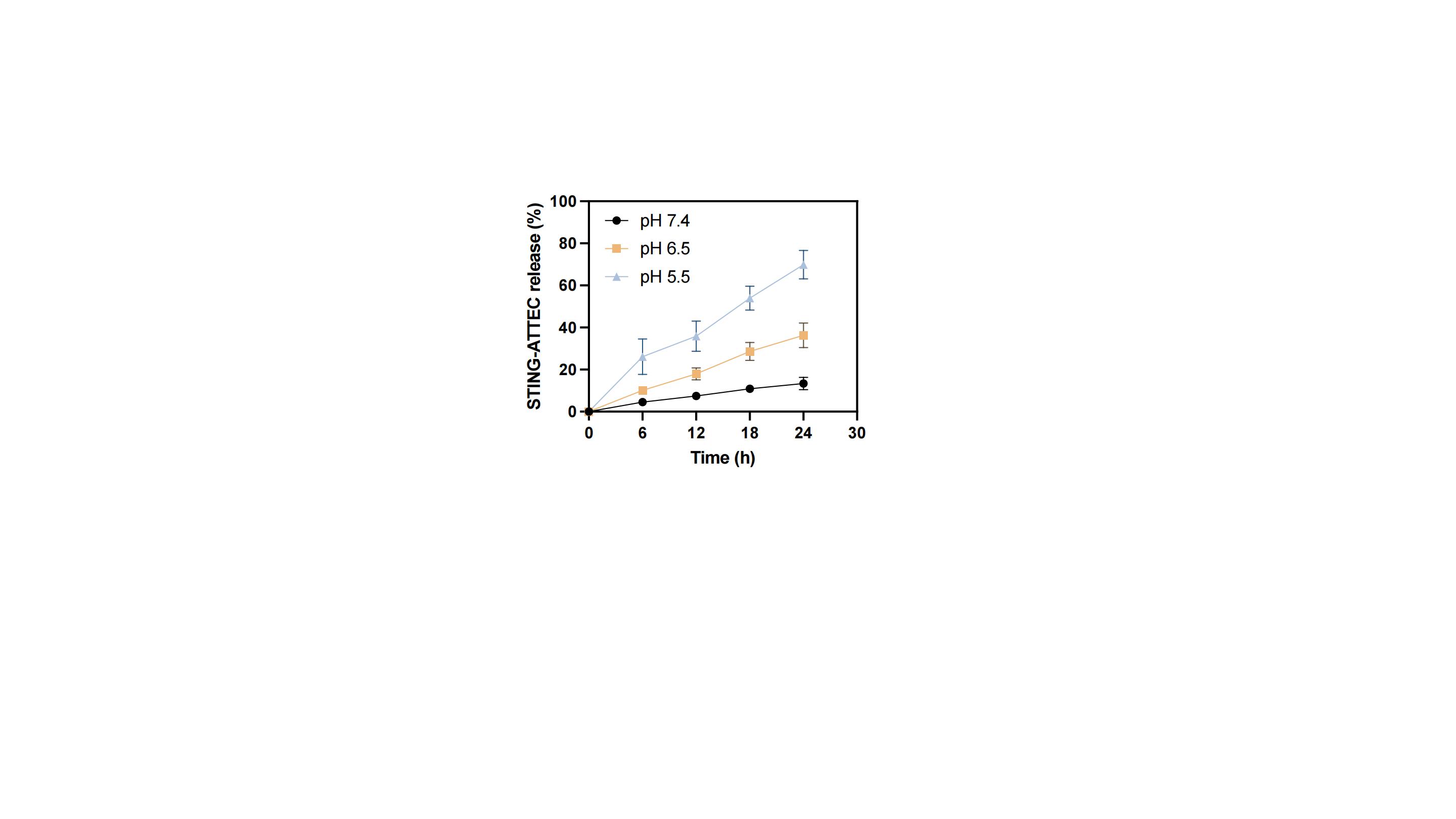


**Supplementary Fig. 15** pH-dependent release profile of FA-LNP⁺@STING-ATTEC (n=3, per group).


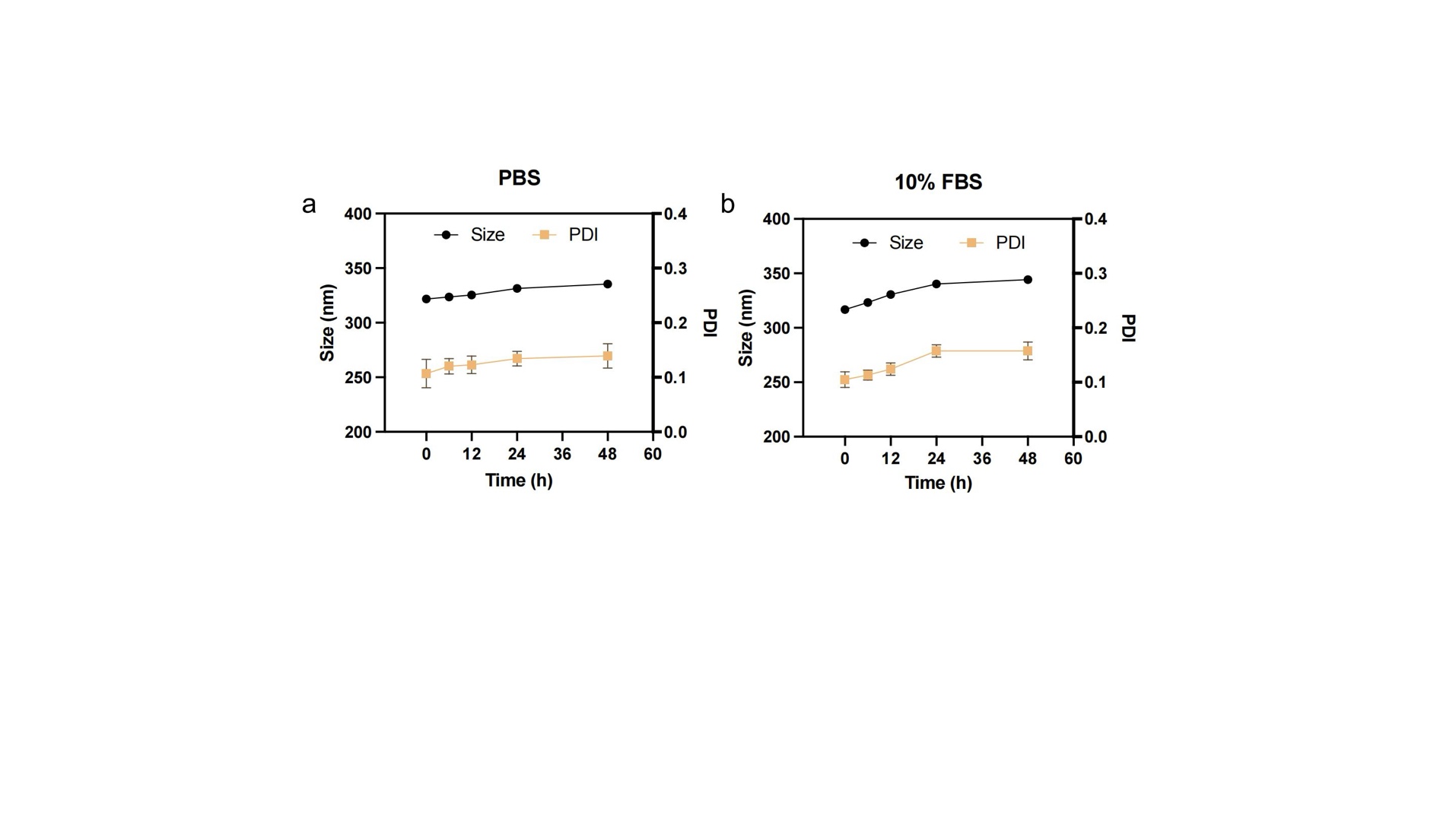


**Supplementary Fig. 16** Colloidal stability of FA-LNP⁺ nanoparticles in PBS (a) and 10% FBS (b) (n=3, per group).


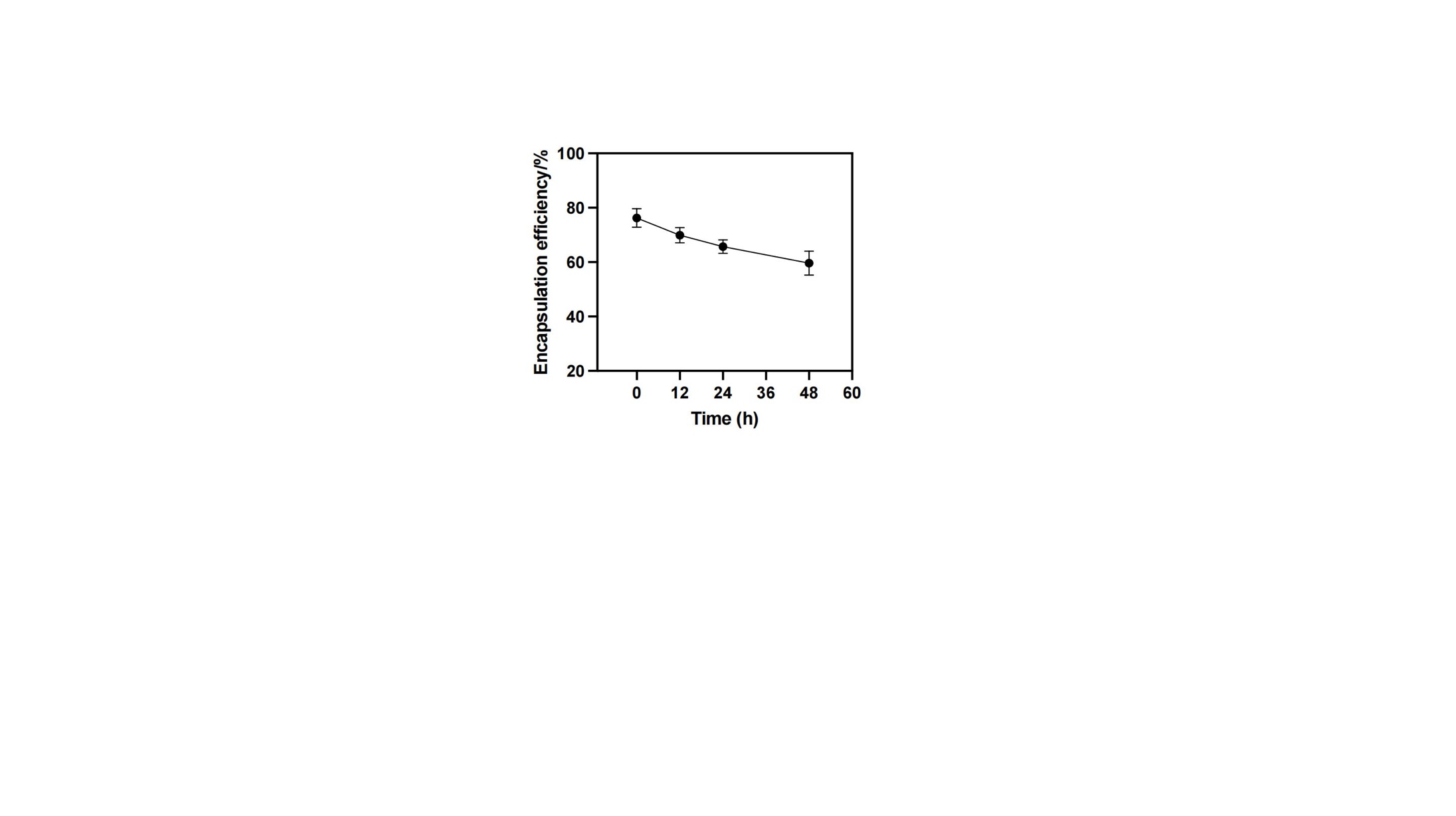


**Supplementary Fig. 17** Time-dependent change of encapsulation efficiency over 48 h (n=3, per group).


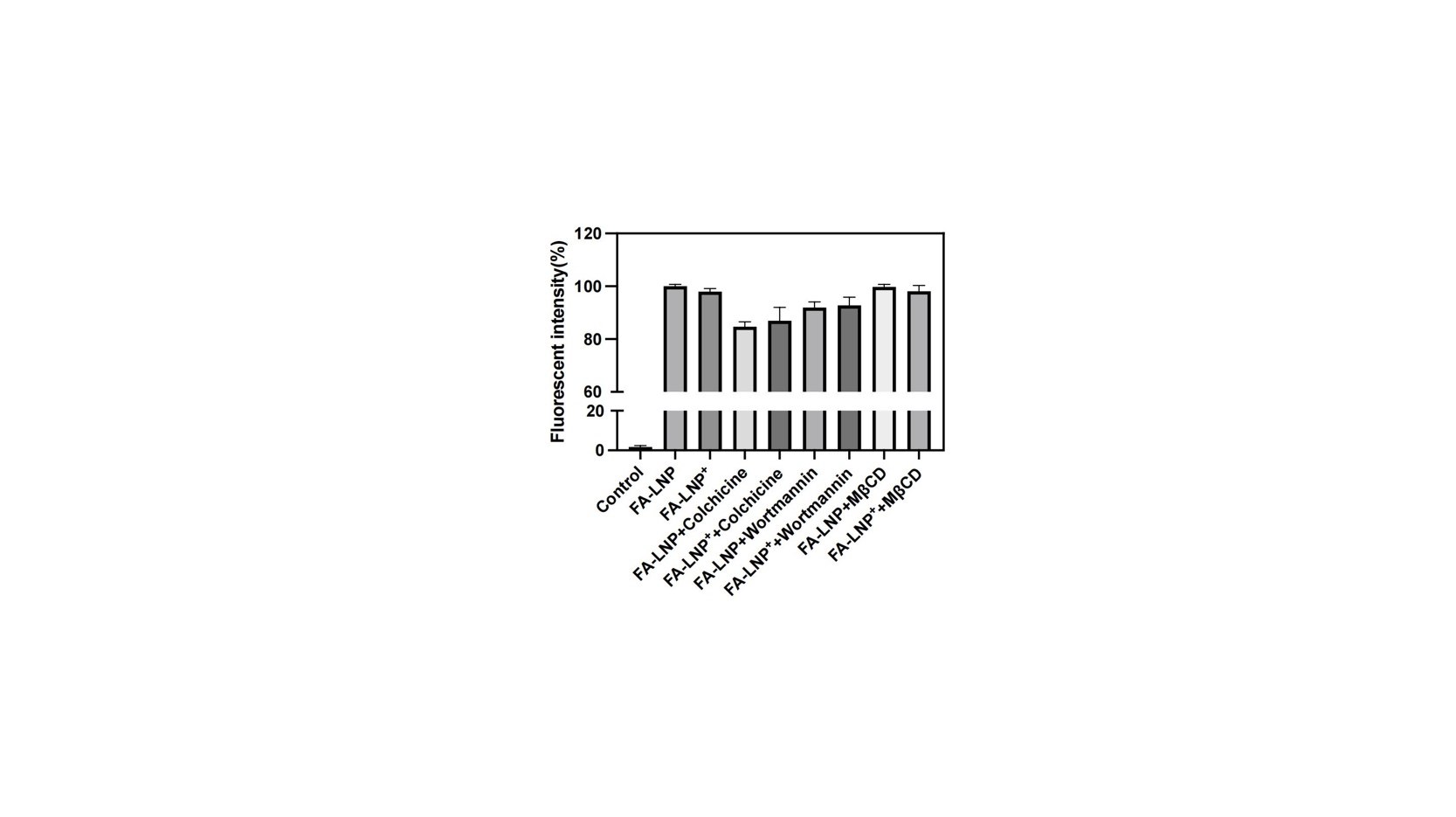


**Supplementary Fig. 18** Flow cytometry quantification of FA-LNP and FA-LNP^+^ uptake in cells treated with different endocytosis inhibitors (n=3, per group).


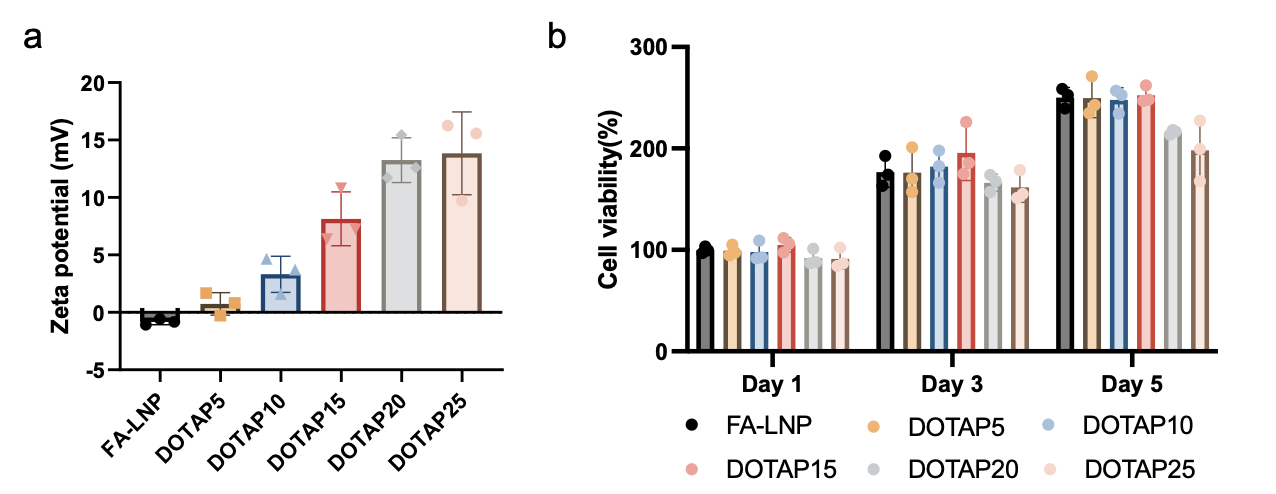


**Supplementary Fig. 19** (a) Zeta potential measurements of FA-LNPs and DOTAP-incorporated FA-LNP formulations. In DOTAP-containing formulations, the molar ratio of DOTAP:D-Lin-MC3-DMA:DSPC:cholesterol:DSPE-PEG-folate was adjusted accordingly (e.g., DOTAP5 = 5:45:10:38.5:1.5, DOTAP10=10:40:10:38.5:1.5) (n=3, per group). (b) Cell viability assessment of FA-LNPs and DOTAP-incorporated FA-LNP formulations by CCK-8 assay (n=3, per group).


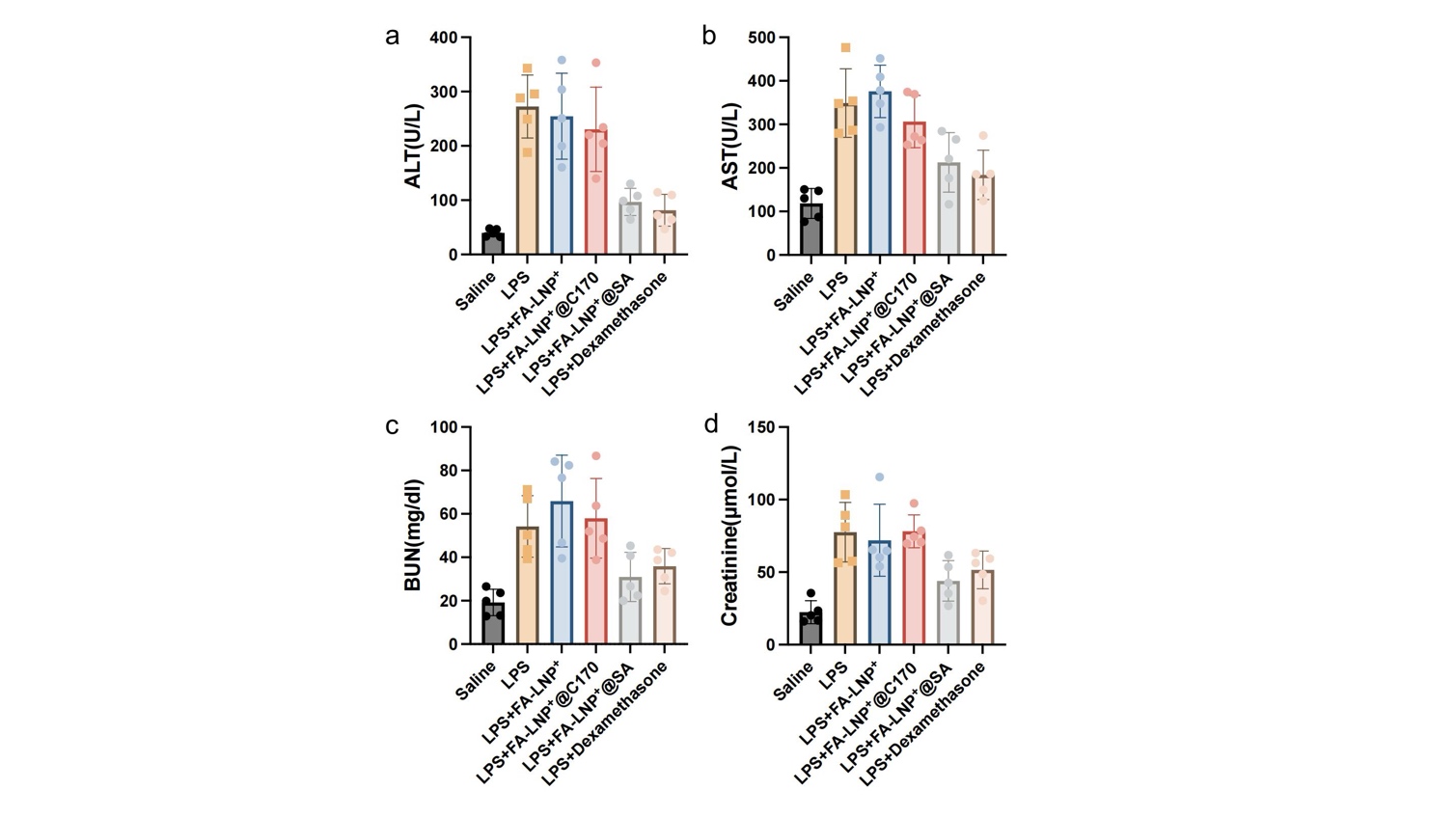


**Supplementary Fig. 20** Serum levels of ALT (a), AST (b), BUN (c), and creatinine (d) in mice from the indicated treatment groups (n=5, per group).


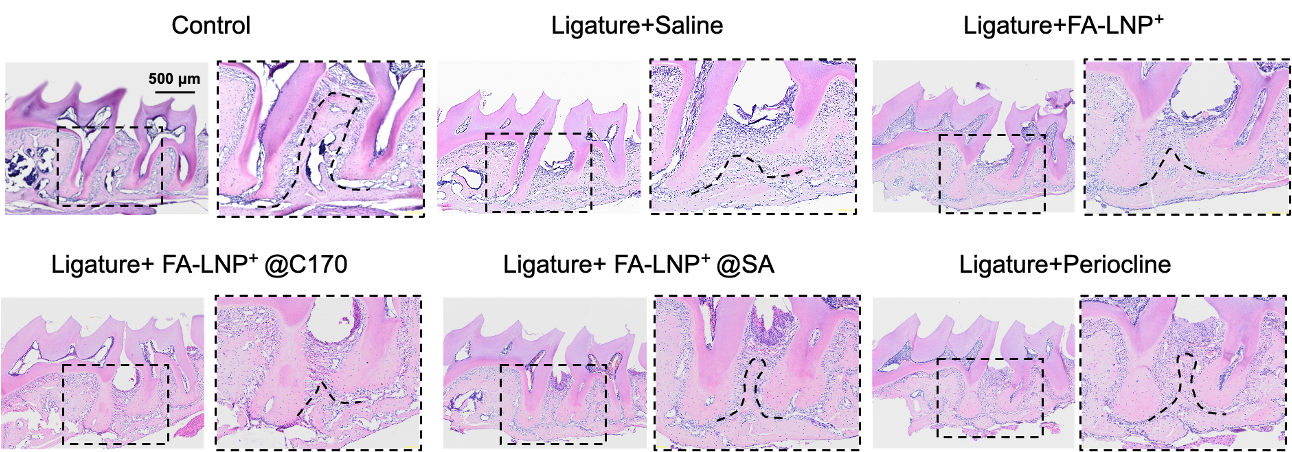


**Supplementary Fig. 21** H&E staining of alveolar bone tissues.

**Supplementary Fig. 22** Measurement of cementoenamel junction to alveolar bone crest (CEJ-ABC) distance (n=5, per group).


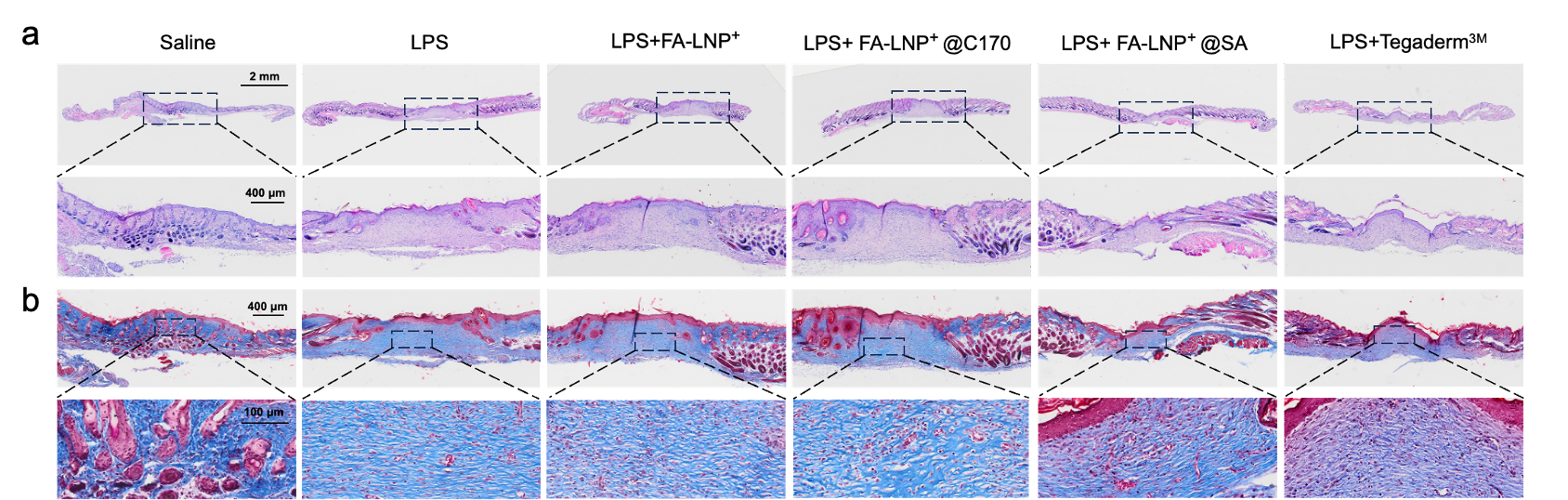


**Supplementary Fig. 23** (a) H&E staining of skin wound healing areas. (b) Masson’s trichrome staining of skin wound healing areas.

**Supplementary Reference**

1. Roh, T. *et al.* Fucosylated haptoglobin promotes inflammation via mincle in sepsis: An observational study. *Nat Commun* **16**, 1342 (2025).

2. Oh, J., Kim, Y., Son, H., Kim, Y. H. & Kim, H. Comparative transcriptome analysis of periodontitis and peri‐implantitis in human subjects. *Journal of Periodontology* **95**, 337–349 (2024).

3. Johnsson, H., Cole, J., Siebert, S., McInnes, I. B. & Graham, G. Cutaneous lesions in psoriatic arthritis are enriched in chemokine transcriptomic pathways. *Arthritis Res Ther* **25**, 73 (2023).

4. Zhang, K. *et al.* TREM2hi resident macrophages protect the septic heart by maintaining cardiomyocyte homeostasis. *Nat Metab* **5**, 129–146 (2023).

5. Cao, Y. *et al.* STING contributes to lipopolysaccharide-induced tubular cell inflammation and pyroptosis by activating endoplasmic reticulum stress in acute kidney injury. *Cell Death Dis.* **15**, 217 (2024).

6. Yang, L. *et al.* Pimpinellin ameliorates macrophage inflammation by promoting RNF146 ‐mediated PARP1 ubiquitination. *Phytother. Res.* ptr.8135 (2024) doi:10.1002/ptr.8135.

7. Xin, L. *et al.* Four-Octyl itaconate ameliorates periodontal destruction via Nrf2-dependent antioxidant system. *Int. J. Oral Sci.* **14**, 27 (2022).

8. Peng, Z. *et al.* Integrated endotoxin-adsorption and antibacterial properties of platelet-membrane-coated copper silicate hollow microspheres for wound healing. *Journal of Nanobiotechnology* **19**, 383 (2021).
